# Direct fluorescent labeling of NF186 and Na_V_1.6 in living primary neurons using bioorthogonal click chemistry

**DOI:** 10.1242/jcs.260600

**Published:** 2023-06-28

**Authors:** Nevena Stajković, Yuanyuan Liu, Aleksandra Arsić, Ning Meng, Hang Lyu, Nan Zhang, Dirk Grimm, Holger Lerche, Ivana Nikić-Spiegel

**Affiliations:** ^1^ Werner Reichardt Centre for Integrative Neuroscience, University of Tübingen, 72076 Tübingen, Germany; ^2^ Graduate Training Centre of Neuroscience, International Max Planck Research School, University of Tübingen, 72076 Tübingen, Germany; ^3^ Department of Neurology and Epileptology, Hertie Institute for Clinical Brain Research, University of Tübingen, 72076 Tübingen, Germany; ^4^ Virus-Host Interaction Group, Department of Infectious Diseases/Virology, Medical Faculty, University of Heidelberg, Cluster of Excellence CellNetworks, BioQuant, 69120 Heidelberg, Germany; ^5^ German Center for Infection Research and German Center for Cardiovascular Research, partner site Heidelberg, 69120 Heidelberg, Germany

**Keywords:** Axon initial segment, Click chemistry, Neurofascin, Microscopy, Unnatural amino acid, Voltage-gated Na^+^ channel

## Abstract

The axon initial segment (AIS) is a highly specialized neuronal compartment that regulates the generation of action potentials and maintenance of neuronal polarity. Live imaging of the AIS is challenging due to the limited number of suitable labeling methods. To overcome this limitation, we established a novel approach for live labeling of the AIS using unnatural amino acids (UAAs) and click chemistry. The small size of UAAs and the possibility of introducing them virtually anywhere into target proteins make this method particularly suitable for labeling of complex and spatially restricted proteins. Using this approach, we labeled two large AIS components, the 186 kDa isoform of neurofascin (NF186; encoded by *Nfasc*) and the 260 kDa voltage-gated Na^+^ channel (Na_V_1.6, encoded by *Scn8a*) in primary neurons and performed conventional and super-resolution microscopy. We also studied the localization of epilepsy-causing Na_V_1.6 variants with a loss-of-function effect. Finally, to improve the efficiency of UAA incorporation, we developed adeno-associated viral (AAV) vectors for click labeling in neurons, an achievement that could be transferred to more complex systems such as organotypic slice cultures, organoids, and animal models.

## Teaser

**Summary:** By using genetically encoded unnatural amino acids and click chemistry, we developed minimally invasive tags for live-cell labeling of large neuronal transmembrane proteins and ion channels.

## INTRODUCTION

The axon initial segment (AIS) is a highly specialized neuronal compartment responsible for the generation of action potentials (Leterrier, 2018). This unique role is mediated by the accumulation of voltage-gated ion channels at high density in the AIS (Kole et al., 2008). Particularly important among these channels is Na_V_1.6 (encoded by *Scn8a*), the most abundant voltage-gated Na^+^ channel isoform in the adult human brain (Sole and Tamkun, 2020). Clustering of Na_V_1.6 occurs at the distal AIS (Hu et al., 2009) through interactions with the membrane domain of ankyrin G (ankG; also known as ANK3) (Leterrier, 2018). AnkG acts as an adaptor that anchors Na_V_1.6 and other important AIS components, such as the 186 kDa neurofascin isoform (NF186; encoded by *Nfasc*), to the underlying cytoskeleton (Leterrier, 2018). As revealed by super-resolution microscopy, AIS components are evenly spaced along the AIS with a periodicity of ∼190 nm (Leterrier et al., 2015; Xu et al., 2013).

Proper neuronal activity requires that Na_V_1.6 and NF186 both function and localize correctly on a subcellular level. Indeed, genetic variations and secondary alterations of Na_V_1.6 have been implicated in neurological disorders, such as epilepsy, autism, intellectual disability, movement disorders and multiple sclerosis (Craner et al., 2004; Johannesen et al., 2022; Meisler et al., 2021), and auto-antibodies against NF186 have been found in patients with multiple sclerosis and chronic inflammatory demyelinating polyradiculoneuropathy (Kira et al., 2019).

**Fig. 1.**
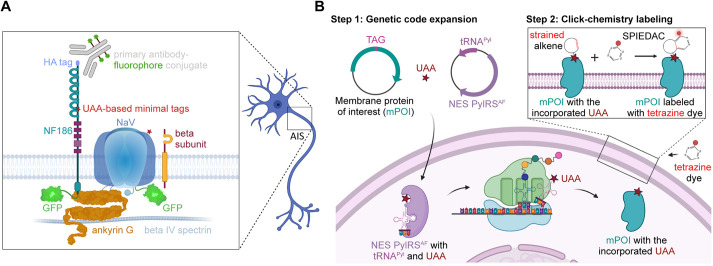
**Overview of the approaches for live-cell labeling of the AIS.** (A) Schematic illustration of different approaches for the live-cell labeling of the AIS – fluorescent protein fusions, antibodies against extracellular epitopes and UAA-based minimal tags. (B) Schematic illustration of genetic code expansion and click-chemistry-based labeling of transmembrane proteins. In step 1, mammalian cells are transfected with plasmids encoding a membrane protein of interest [mPOI; with an in-frame amber (TAG) stop codon] and orthogonal translational machinery (such as NES PylRS^AF^/tRNA^Pyl^ pair). During protein translation, the NES PylRS^AF^/tRNA^Pyl^ pair enables site-specific incorporation of the UAA into the mPOI in response to the TAG stop codon. In step 2, the UAA is coupled to a cell-impermeable fluorescent tetrazine dye in a bioorthogonal SPIEDAC click reaction. The schemes were created with BioRender.com.

To study the trafficking and dynamics of Na_V_1.6 and NF186, several live-cell labeling approaches have been developed (Akin et al., 2015, 2016; Dzhashiashvili et al., 2007; Gasser et al., 2012; Ghosh et al., 2020; Liu et al., 2022; Sole et al., 2019; Susuki et al., 2013; Zhang et al., 1998). The most widely used approach relies on generating genetic fusions with fluorescent proteins (FPs; Fig. 1A) (Akin et al., 2015; Dzhashiashvili et al., 2007; Gasser et al., 2012; Ghosh et al., 2020; Zhang et al., 1998). The main advantages of FP fusions are their high specificity and compatibility with live-cell imaging. However, most fusions are made by placing the FP at either the N- or C-terminus of the target protein. However, those terminal domains of AIS components frequently participate in channel inactivation, targeting and localization, or include binding regions for various regulatory proteins. Therefore, some of these interactions are at risk of being impaired by relatively large FP tags (∼30 kDa). Indeed, it has been reported that the fusion of GFP to the C-terminus of NF186 results in its mislocalization (Dumitrescu et al., 2016; Dzhashiashvili et al., 2007). In addition to FP fusions, other methods for live labeling of Na_V_1.6 and other voltage-gated Na^+^ channel isoforms have been established. One such approach for specifically labeling Na_V_ channels is based on the incorporation of a 17-amino-acid-long biotinylated domain (BAD) into their extracellular domains. The BAD domain, if biotinylated by bacterial biotin ligase, can be labeled with nonpermeable streptavidin-conjugated dyes. This method has been successfully used to label Na_V_1.6 (Akin et al., 2015), Na_V_1.9 (Akin et al., 2019) and Na_V_1.7 (Sizova et al., 2020) in primary neurons or mammalian cell lines. The disadvantages of this approach are the bulkiness and the large size of streptavidin (∼60 kDa). Recently, GFP and self-labeling HaloTags fused to the C-termini of endogenous Na_V_ channels have been utilized for live-cell microscopy studies (Fréal et al., 2022 preprint; Liu et al., 2022). This labeling approach, based on CRISPR/Cas9 genome editing, offers a great opportunity to study trafficking and localization of endogenous Na^+^ channels, albeit with the familiar disadvantage related to the size of these tags (∼30 and 33 kDa, respectively).

As an alternative to genetic fusions, immunostaining with fluorophore-conjugated antibodies can be used to label the AIS in living neurons. These antibodies recognize either the extracellular domains of endogenous AIS proteins or short tags attached to the extracellular domains of recombinant or endogenous AIS components (Fig. 1A) (Dumitrescu et al., 2016; Dzhashiashvili et al., 2007; Evans et al., 2015; Freal et al., 2019; Hedstrom et al., 2008; Liu et al., 2022; Schafer et al., 2009; Torii et al., 2020). However, antibodies, owing to their multivalence, can induce crosslinking, which makes them unsuitable for studying the dynamics of the AIS, such as its plasticity (Dumitrescu et al., 2016). Furthermore, antibodies are not always sufficiently specific to distinguish proteins that are closely related, such as different Na_V_ isoforms. In view of these limitations, it would be beneficial to develop other approaches for direct and minimally invasive live-cell labeling of AIS components.

We and other researchers have previously developed unnatural amino acid (UAA)-based minimal tags for live-cell protein labeling in mammalian cells (Lang et al., 2012a,b; Nikic et al., 2014; Plass et al., 2012; Uttamapinant et al., 2015). Single UAAs are installed site-specifically in a protein and then labeled with a small-molecule fluorescent dye. Under the control of genetic code expansion (Chin, 2017; Nikic-Spiegel, 2020; Wang, 2017), UAAs carrying strained alkene moieties are co-translationally incorporated into a protein of interest in response to an in-frame amber stop codon (Fig. 1B). In a subsequent step, fluorescent dyes are covalently attached to the UAA residue with click chemistry reactions. One such reaction is the bioorthogonal catalyst-free strain-promoted inverse-electron demand Diels–Alder cycloaddition (SPIEDAC) between the alkene and a tetrazine derivative of a fluorescent dye. Owing to its high reaction rates and bioorthogonality, SPIEDAC is particularly useful chemistry for live-cell labeling (Fig. 1B). In recent years, this type of labeling has emerged as one of the most powerful methods for the minimally invasive labeling of both extracellular and intracellular proteins in standard cell lines. We have recently established this in primary neurons by labeling the neuronal cytoskeleton (Arsić et al., 2022), and others have labeled small (34–45 kDa) transmembrane AMPA receptor regulatory proteins (Bessa-Neto et al., 2021).

In this study, we utilized SPIEDAC to label two large transmembrane AIS proteins, NF186 (∼186 kDa) and Na_V_1.6 (∼260 kDa), with small dyes, which allowed us to perform fixed- and live-cell confocal microscopy, and super-resolution microscopy. SPIEDAC labeling also enabled us to investigate the localization of wild-type (WT) and pathogenic Na_V_1.6 variants. Finally, we developed adeno-associated viral (AAV) vectors that delivered with high efficiency the components required for genetic code expansion into primary neurons.

## RESULTS

### Genetic code expansion and click labeling of NF186 in the ND7/23 cell line

**Fig. 2.**
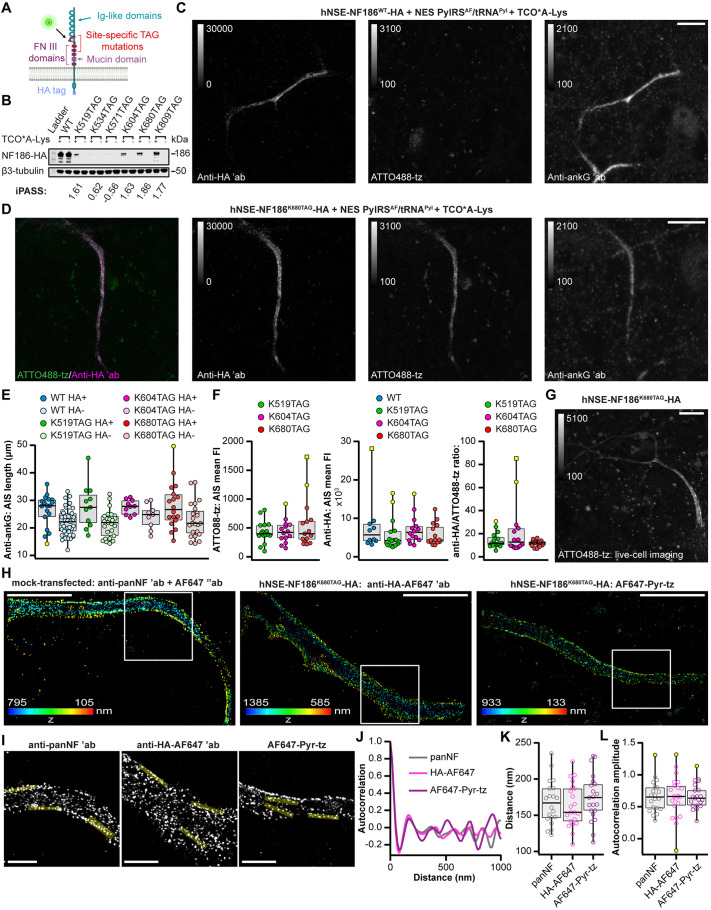
**Genetic code expansion and click labeling of NF186.** (A) Schematic illustration of the NF186 structure with clickable labeling sites (red) and the C-terminal HA tag. The scheme was created with BioRender.com. (B) Western blot analysis of lysates of ND7/23 cells co-expressing NES PylRS^AF^/tRNA^Pyl^ and the CMV-NF186–HA constructs indicated. Recombinant NF186–HA was detected with an anti-HA antibody. β3-tubulin was used as a loading control. (C,D) Confocal images of rat neurons at days *in vitro* (DIV) 11 expressing the plasmids indicated. Before imaging, living neurons were labeled with ATTO488-tz, fixed, and immunostained with anti-HA and anti-ankG antibodies. (E) Distribution of the AIS lengths measured in confocal images of the anti-ankG-immunostained neurons expressing the indicated hNSE-NF186–HA plasmids (HA+) and the neighboring untransfected (HA−) neurons. No significant differences between the groups were detected [*P*>0.05, Kruskal–Wallis test followed by Dunn post-hoc analysis with Bonferroni correction for multiple comparisons; number (*n*) of analyzed neurons: *n*_WT HA+_=18, *n*_WT HA−_=41, *n*_K519TAG HA+_=11, *n*_K519TAG HA−_=25, *n*_K604TAG HA+_=10, *n*_K604TAG HA−_=9, *n*_K680TAG HA+_=18, *n*_K680TAG HA−_=23]. (F) Distribution of the mean ATTO488-tz and anti-HA fluorescence intensities (FI), and anti-HA/ATTO488-tz ratio measured in confocal images of neurons expressing the hNSE-NF186–HA constructs indicated. No significant differences between the groups were detected [*P*>0.05, Kruskal–Wallis test; *n*_WT_=10, *n*_K519TAG_=16, *n*_K604TAG_=14, *n*_K680TAG_=15 neurons]. (G) Representative confocal image of an ATTO488-tz-labeled living neuron at DIV 11 expressing NF186^K680TAG^–HA. (H,I) Representative 3D dSTORM images of rat neurons at DIV 12 expressing the indicated constructs and click labeled with AF647-pyr-tz or immunostained with the antibodies indicated. The *z*-positions are color-coded according to the height maps (bottom left). The height maps show minimal and maximal *z* position values. Boxed regions from H with the representative overlaid line ROIs that were used for autocorrelation analysis are shown in I. (J) The averaged autocorrelation curves from 1-µm-long intensity profiles along AISs expressing the constructs indicated (*n*_panNF_=20, *n*_HA-AF647_=21, *n*_AF647-pyr-tz_=21 individual ROIs measured from *n*_panNF_=8, *n*_HA-AF647_=10, *n*_AF647-pyr-tz_=11 neurons). Mean±s.e.m. spacing values (*s*) are as follows: *s*_panNF_=170.1±23.5, *s*_HA-AF647_=168.8±19.3, *s*_AF647-pyr-tz_=174.3±28.5. (K,L) Distribution of spacing (K) and autocorrelation amplitude (L) values of individual ROIs for the conditions indicated. No significant differences between the groups were detected [*P*>0.05, one-way ANOVA with Tukey's post-hoc analysis (K) or Kruskal–Wallis test (L); *n*_panNF_=20, *n*_HA− AF647_=21, *n*_AF647-pyr-tz_=21 individual ROIs measured from *n*_panNF_=8, *n*_HA-AF647_=10, *n*_AF647-pyr-tz_=11 neurons]. All box plots indicate the median (the black lines inside the box), the 25th and 75th percentiles (the box boundaries), single data points (dots), outliers (yellow dots), and the furthest outliers (yellow squares). Whisker lengths are defined by the minimum and maximum data points. The *Z*-stack confocal images are shown as maximum intensity projections in all panels. The brightness and contrast of the panels were linearly adjusted as indicated by the look-up-table (LUT) intensity scale bar. The LUT intensity scale bars show the minimum and maximum gray values. Dots represent individual neurons (E,F) or axonal ROIs (K,L). Data were collected in two (J, K, L), three (F) or four (E) independent experiments. Details of the statistical analysis are given in Tables S1–S6. Scale bars: 10 µm (C, D, G), 5 µm (H), 1 µm (I). ′ab, primary antibody; ″ab, secondary antibody.

To establish live-cell fluorescent labeling of the AIS using click chemistry, we first focused on one of its smaller components, NF186. To identify the optimal position at which to incorporate the UAA, we tested multiple residues in a plasmid that encodes a C-terminal hemagglutinin (HA) tag fusion of the WT rat NF186 driven by a cytomegalovirus (CMV) promoter (hereafter referred to as ‘CMV-NF186^WT^–HA’). Optimizing the position at which a protein of interest is labeled is necessary if we consider that the amber codon suppression efficiency depends on the surrounding sequence (Bartoschek et al., 2021) and that the efficiency of the click chemistry depends on the UAA being accessible to the tetrazine dye. SWISS-MODEL (Bienert et al., 2017) was used to select six potential positions for the UAA incorporation in the extracellular domain of NF186 (Fig. 2A), based on the crystal structure of a titin fragment (PDB ID: 3B43; von Castelmur et al., 2008). Then, we introduced the corresponding amber (TAG) codons into the rat *Nfasc* gene by site-specifically mutating lysine (K) residues, as indicated in the following constructs: NF186^K519TAG^, NF186^K534TAG^, NF186^K571TAG^, NF186^K604TAG^, NF186^K680TAG^ and NF186^K809TAG^.

We have recently shown that the rodent neuroblastoma ND7/23 cell line is a suitable host for genetic code expansion and click labeling of neuronal proteins (Arsić et al., 2022). We used the same system to identify the optimal construct for NF186 click labeling. We co-transfected ND7/23 cells with CMV-NF186^TAG^–HA constructs and the plasmid for clickable UAA incorporation. The latter encodes the Y306A/Y384F (AF) double mutant of the *Methanosarcina mazei-*derived pyrrolysyl (Pyl) tRNA synthetase fused to a nuclear export signal (NES PylRS^AF^) and its cognate amber codon suppressor tRNA^Pyl^. Immunostaining with an anti-HA antibody, confocal imaging (Figs S1 and S2) and western blot analysis (Fig. 2B) revealed that all six constructs were expressed in the presence of the UAA *trans*-cyclooct-2-en-L-lysine (TCO*A-Lys), whereas no expression was detected in its absence. The variants showed different expression levels (Fig. 2B), which could be correlated to their identification of permissive amber sites for suppression (iPASS) scores. iPASS scores predict the efficiency of UAA incorporation and were calculated using a recently developed tool (Bartoschek et al., 2021). Finally, live-cell click labeling with an ATTO488 tetrazine derivative (ATTO488-tz) showed that all of the constructs can be labeled in ND7/23 cells (Fig. S1). As expected, no click labeling was observed if the UAA was omitted (Fig. S2).

### Genetic code expansion and click labeling of NF186 in living primary neurons

Having established click labeling of NF186^TAG^–HA in the ND7/23 cell line, we aimed to label NF186 in primary rat cortical neurons. Typically, we would consider the best-expressing TAG mutant with the highest labeling efficiency from the ND7/23 screen to be the best candidate for use in primary neurons. However, it is well established that overexpression of certain AIS components, such as ankG and NF186, can lead to their mislocalization or can result in an abnormally elongated AIS (Dumitrescu et al., 2016; Galiano et al., 2012; Hamdan et al., 2020; Jenkins et al., 2015). For those reasons, we considered additional factors, such as the subcellular localization of recombinant NF186. Although we first anticipated that the lower expression of NF186^TAG^–HA mutants compared to NF186^WT^–HA would overcome any mislocalization that might occur, this was not the case. Microscopy analysis of primary neurons showed that both NF186^WT^–HA (Fig. S3A) and NF186^TAG^–HA mutants (Fig. S3B) were ectopically localized along distal axons.

To improve the localization of the NF186–HA constructs, we replaced the CMV promoter with the weaker human neuron-specific enolase promoter (hNSE) in the NF186^WT^–HA and NF186^TAG^–HA constructs (hereafter referred to as ‘hNSE-NF186^WT^–HA’ and ‘hNSE-NF186^TAG^–HA’, respectively). This promoter has been previously used to lower the expression level and optimize localization of recombinant NF186 in neurons (Hamdan et al., 2020). After we confirmed that the hNSE promoter reduced the mislocalization of NF186 (the number of neurons overexpressing NF186 in dendrites and axons decreased from 65.5% to 48.2%), we expressed and labeled hNSE-NF186–HA with ATTO488-tz (Fig. 2C,D; Fig. S4). Immunostaining and confocal microscopy confirmed that only the hNSE-NF186^TAG^–HA and not hNSE-NF186^WT^–HA could be labeled with click chemistry. Furthermore, these experiments revealed that not all the NF186^TAG^ mutants were equally well expressed and labeled with ATTO488-tz. In line with the western blot analysis and their low (<1) iPASS scores (Fig. 2B), we observed fewer neurons expressing NF186^K534TAG^–HA and NF186^K571TAG^–HA, and click labeling was either weak or completely absent (Fig. S4). Therefore, we excluded those two mutants from our further analysis. Although NF186^K809TAG^–HA (Fig. S4) showed bright click labeling, we excluded it from the analysis owing to its predominantly ectopic expression along the distal axon (not quantified).

To identify the most suitable TAG position for NF186 click labeling among the three remaining mutants (NF186^K519TAG^–HA, NF186^K604TAG^–HA and NF186^K680TAG^–HA), we assessed whether the AIS structure was affected by their overexpression. To that end, we used a MATLAB script custom-written for quantitatively measuring AIS length (Grubb and Burrone, 2010) to compare the AIS of neighboring NF186^WT^ or NF186^TAG^–HA-transfected (HA positive) and untransfected (HA negative) neurons (Fig. 2E). Immunostaining with anti-HA allowed us to identify transfected neurons and anti-ankG served as a transfection-independent marker to identify the AIS in both transfected and surrounding untransfected neurons. Quantitative analysis of AIS length showed no significant differences between the neurons that expressed recombinant NF186^WT^ or NF186^TAG^–HA and the untransfected neurons (Fig. 2E).

Considering that none of the three amber mutants analyzed affected AIS length, we used additional parameters to determine the most suitable position for click labeling of NF186 in primary neurons – the intensities of labeling and anti-HA immunostaining and their ratios. These measurements (Fig. 2F) showed no significant difference between the three constructs. Based on its highest iPASS score (1.83), we then selected NF186^K680TAG^–HA for use in subsequent experiments. This allowed us to perform fixed-cell (Fig. 2D) and live-cell (Fig. 2G) confocal imaging of click-labeled NF186 in primary neurons.

Finally, we investigated whether click labeling of NF186 was compatible with direct stochastic optical reconstruction microscopy (dSTORM). After obtaining dSTORM images (Fig. 2H), we compared NF186 periodicity in mock-transfected neurons [immunostained with anti-pan neurofascin (NF) antibody, which labels endogenous neurofascin, as previously described (D'Este et al., 2017)] and neurons transfected with NF186^K680TAG^–HA. To minimize the difference in the size of labeling tags, instead of using primary and secondary antibody complexes, we compared direct immunofluorescence staining with an Alexa Fluor 647 (AF647)-conjugated primary anti-HA antibody to click labeling with AF647-pyrimidyl-tetrazine (AF647-pyr-tz). The autocorrelation analysis (Fig. 2I–L) revealed no significant difference between the spacing (Fig. 2K) and the degree of periodicity (estimated by the amplitude of the autocorrelation curve; Fig. 2L) of the endogenous panNF staining and recombinant NF186 in AF647-pyr-tz channel and HA channels. Altogether, these results demonstrate a successful application of genetic code expansion and click chemistry for the labeling and conventional and super-resolution imaging of the AIS in primary neurons.

### Click labeling of Na_V_1.6 channels in living primary neurons

**Fig. 3.**
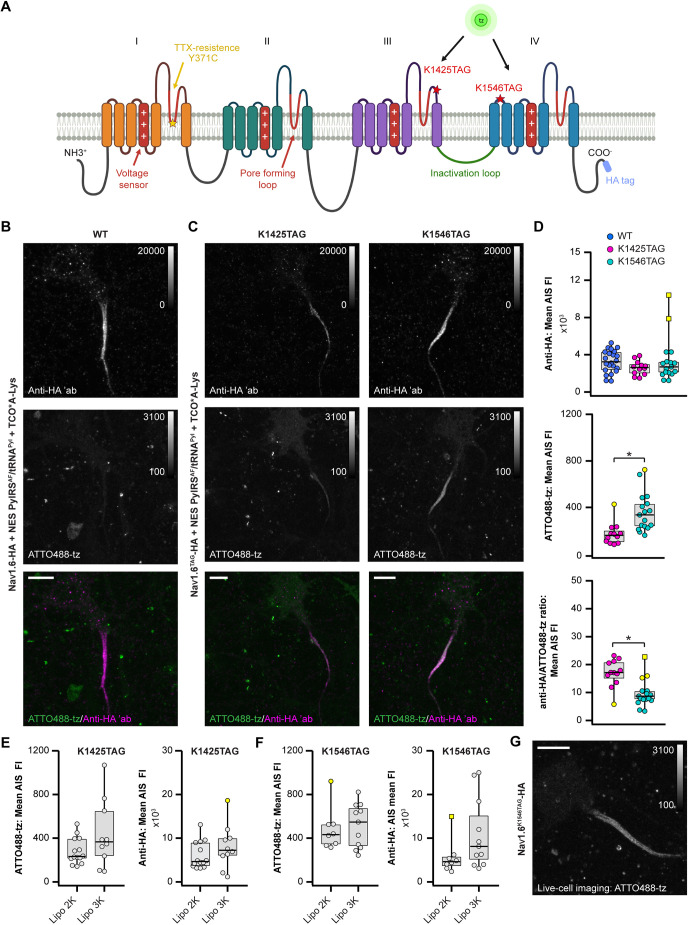
**Genetic code expansion and click labeling of Na_V_1.6 in primary neurons.** (A) Schematic representation of the Na_V_1.6 α subunit with the clickable labeling sites (red stars) and the C-terminal HA tag. The scheme was created with BioRender.com. (B,C) Representative confocal images of rat neurons at DIV 12 expressing the constructs indicated. Prior to imaging, living neurons were labeled with ATTO488-tz, fixed, and immunostained with an anti-HA antibody. (D) Distribution of the mean anti-HA or ATTO488-tz fluorescence intensity (FI), and the anti-HA/ATTO488-tz ratio measured in confocal images of rat neurons expressing the Na_V_1.6–HA constructs indicated. Significant differences are indicated (*P<0.05, Kruskal–Wallis or Mann–Whitney *U* test; *n*_WT_=22, *n*_K1425TAG_=12, *n*_K1546TAG_=17 neurons). (E,F) Distribution of the mean ATTO488-tz or anti-HA FI measured in confocal images of neurons expressing Na_V_1.6^K1425TAG^–HA (E) or Na_V_1.6^K1546TAG^–HA (F) transfected using Lipofectamine 2000 or Lipofectamine 3000. No significant differences between the groups were detected (*P*>0.05, Mann–Whitney *U* test; *n*_K1425TAG-Lipo2K_=14, *n*_K1425TAG-Lipo3K_=10, *n*_K1546TAG-Lipo2K_=8, *n*_K1546TAG-Lipo3K_=11 neurons). All box plots indicate the median (the black lines inside the box), the 25th and 75th percentiles (the box boundaries), the single data points (dots), the outliers (yellow dots) and the furthest outliers (yellow squares). Whisker lengths are defined by the minimum and maximum data points. (G) Representative confocal image of click-labeled living neuron at DIV 12 expressing Na_V_1.6^K1546TAG^–HA. All images were taken as *Z*-stacks and are shown as maximum intensity projections. The brightness and contrast of the panels were linearly adjusted as indicated by the LUT intensity scale bar. The LUT intensity scale bars show the minimum and maximum gray values. Dots represent individual neurons (D–F). Data were collected in three independent experiments. Details of the statistical analysis are given in Tables S7–S13. Scale bars: 10 µm (B,C,G). ′ab, primary antibody.

Once we had established click labeling of NF186, we focused on labeling the alpha subunit of the voltage-gated Na^+^ channel isoform Na_V_1.6. The structure of Na_V_1.6 is highly complex, involving the folding of a single polypeptide chain of ∼2000 amino acids (Fig. 3A) into four homologous domains (I to IV), each of which contains six transmembrane segments (S1 to S6). This complexity adds to the challenges in selecting the position for UAA incorporation. In short, we selected two positions (K1425 and K1546) based on the available literature (see Materials and Methods) (Akin et al., 2015; Meisler et al., 2021; O'Brien and Meisler, 2013). We introduced respective TAG mutations into the corresponding sites of a plasmid encoding WT mouse Na_V_1.6 fused to a C-terminal HA tag (Fig. 3A).

Similar to the experiments with NF186, before using primary neurons, we first wanted to establish the conditions for click labeling Na_V_1.6^TAG^–HA in neuronal cell lines (Fig. S5). We initially tried the ND7/23 cell line, which has been widely used for electrophysiological recordings of Na^+^ currents derived from recombinant tetrodotoxin (TTX)-resistant variants of Na_V_1.6 (Meisler et al., 2021; Sharkey et al., 2009). However, our microscopy experiments revealed that the expression level of Na_V_1.6^WT^–HA (Fig. S5A) and both Na_V_1.6^TAG^–HA mutants (data not shown) on the membrane of ND7/23 cells was low. Given that the transfection efficiency was low and most of the ion channels remained inside the cytoplasm, extracellular labeling with the cell-impermeable ATTO488-tz was unsuccessful (data not shown). In complementary functional experiments, we performed whole-cell patch clamping. First, we rendered the WT and TAG Na_V_1.6–HA constructs TTX resistant by introducing a Y371C mutation (Fig. 3A) (Leffler et al., 2005; Liu et al., 2019). Most of the ND7/23 cells measured exhibited peak Na^+^ current amplitudes of less than 0.5 nA in the presence of TTX (data not shown), confirming the microscopy results.

To obtain higher levels of expression, we then tested click labeling conditions in the murine neuroblastoma N1E-115-1 cell line (Fig. S5B–D). These cells have also been used for electrophysiological studies of voltage-gated Na^+^ channels, including Na_V_1.6 (Hirsh and Quandt, 1996; Vega et al., 2013). Immunostaining with an anti-HA antibody revealed that the expression of Na_V_1.6^WT^–HA on the membranes of N1E-115-1 cells was higher than on ND7/23 cells (Fig. S5A–C). However, click labeling with ATTO488-tz was not successful on either Na_V_1.6^K1425TAG^–HA nor Na_V_1.6^K1546TAG^–HA (Fig. S5D), most likely due to insufficient expression of these constructs.

We therefore reasoned that neuroblastoma cell lines might not be ideal hosts for click labeling and microscopy studies of Na_V_1.6^TAG^, and that a more native environment was required. Thus, we attempted to express and click label Na_V_1.6^TAG^–HA in rat primary neurons (Fig. 3B,C; Fig. S6). As expected, the WT protein was expressed in the presence and in the absence of the UAA, but click labeling was not observed (Fig. 3B; Fig. S6A). Furthermore, we observed that both Na_V_1.6^TAG^–HA mutants were expressed and click-labeled in the AIS of the neurons (Fig. 3C). If the UAA was omitted, expression and click labeling of amber mutants were not detected (Fig. S6A). In addition to the specific AIS labeling, we occasionally observed ATTO488 signal in cell debris and in the somatic region of some transfected neurons (Fig. 3B,C). Quantitative analysis showed that some of the neurons expressing Na_V_1.6 are permeable to ATTO488-tz, even in the absence of the genetic code expansion machinery and UAA (Fig. S6C). Although an important consideration, this has no bearing on our findings – it does not depend on amber codon suppression and, in the neurons that were affected, the intensity of cytosolic ATTO488 was lower than the specific AIS signal.

Further quantitative analysis of the anti-HA and ATTO488 intensities and their ratios revealed that despite having similar expression levels, the click labeling efficiency of Na_V_1.6^K1425TAG^–HA was lower than that of Na_V_1.6^K1546TAG^–HA (Fig. 3D). After changing the transfection reagent from Lipofectamine 2000 to Lipofectamine 3000, we measured a non-significant trend towards higher expression levels and labeling intensities of both constructs (Fig. 3E,F; Fig. S6B). However, the number of neurons transfected with Lipofectamine 3000 was lower than when Lipofectamine 2000 was used (five versus 15 neurons per well of an eight-well Lab-Tek II chambered cover glass); thus we continued to use Lipofectamine 2000. In combination with the high labeling efficiency of Na_V_1.6^K1546TAG^–HA, this facilitated the confocal imaging of the living click-labeled neurons (Fig. 3G). In conclusion, we successfully applied a combination of genetic code expansion and click chemistry for fluorescent labeling and imaging of the voltage-gated Na^+^ channel isoform Na_V_1.6 in living primary neurons.

### Characterization of the localization and functionality of clickable Na_V_1.6^TAG^–HA amber mutants

**Fig. 4.**
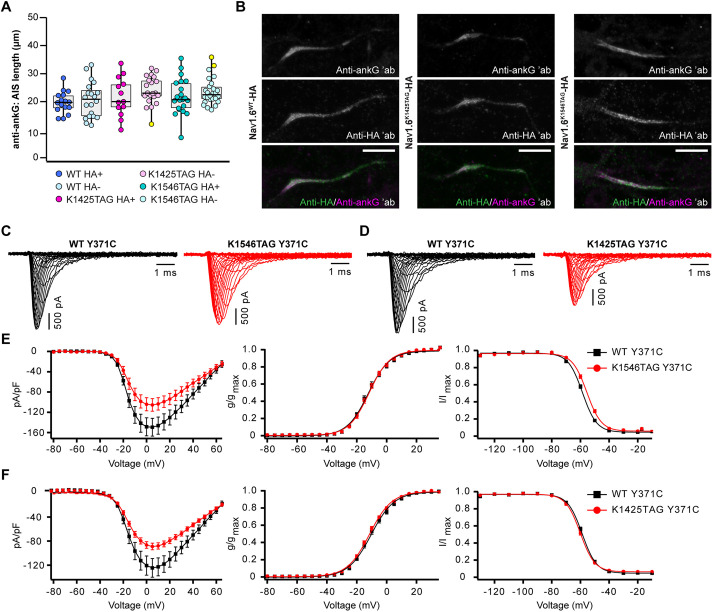
**Characterization of the expression and functionality of Na_V_1.6^TAG^–HA.** (A) Distribution of the AIS lengths measured in confocal images of anti-ankG-immunostained rat neurons expressing the indicated Na_V_1.6–HA constructs (HA+) and the corresponding neighboring untransfected (HA−) neurons. The box plots indicate the median (the black lines inside the box), the 25th and 75th percentiles (the box boundaries), the single data points (dots), and the outliers (yellow dots). Whisker lengths are defined by the minimum and maximum data points. No significant differences between the groups were detected (*P*>0.05, Kruskal–Wallis test; *n*_WT HA+_=16, *n*_WT HA−_=20, *n*_K1425TAG HA+_=13, *n*_K1425TAG HA−_= 21, *n*_K1546TAG HA+_=21, *n*_K1546TAG HA−_=26 neurons). Dots represent individual neurons. Data were collected from three independent experiments. Details of the statistical analysis are given in Table S14. (B) Representative images from three repeats of rat neurons immunostained with anti-ankG and anti-HA antibodies used for the quantitative analysis in A. All images are shown as maximum intensity projections. Scale bars: 10 µm. ′ab, primary antibody. (C) Representative Na^+^ current traces obtained from N1E-115-1 cells expressing either Na_V_1.6^WT,Y371C^–HA (black) or Na_V_1.6^K1546TAG,Y371C^–HA (red). (D) Representative Na^+^ current traces obtained from N1E-115-1^β1β2^ stable cells expressing either Na_V_1.6^WT, Y371C^–P2A–eGFP (black) or Na_V_1.6^K1425TAG,Y371C^–P2A–eGFP (red). (E) Peak Na^+^ current densities as normalized to cell capacitance are plotted versus voltage (left; *n*_WT_=20, *n*_K1546TAG_=18 cells), voltage-dependence of activation (middle; *n*_WT_=20, *n*_K1546TAG_=18 cells) and voltage-dependence of fast inactivation (right; *n*_WT_=20, *n*_K1546TAG_=17 cells) for K1546TAG (red) versus WT comparison (black). Compared to WT channels K1546TAG amber mutation caused a significant (*P*<0.05) depolarizing shift of voltage-dependent fast inactivation. (F) Peak Na^+^ current densities as normalized to cell capacitance are plotted versus voltage (left; *n*_WT_=18, *n*_K1425TAG_=20 cells), voltage-dependence of activation (middle; *n*_WT_=18, *n*_K1425TAG_=20 cells) and voltage-dependence of fast inactivation (right; *n*_WT_=18, *n*_K1425TAG_=20 cells) for K1425TAG (red) versus WT comparison (black). Compared to WT channels, K1425TAG amber mutation caused a significant (*P*<0.05) current density reduction compared to the WT channels. The data shown in E, F were analyzed with an unpaired two-tailed Student's *t*-test or Mann–Whitney *U* test. The lines represent the Boltzmann functions fit to the data points. Shown are mean±s.e.m. (E,F). Details of the statistical analysis are given in Table S15. Data were recorded in six (E) or five (F) independent experiments.

We next investigated whether the AIS structure was affected by overexpression of the clickable Na_V_1.6 variants. As for NF186, we compared the AIS length of the transfected neurons with that of surrounding untransfected neurons. The length of the AIS in neurons expressing recombinant Na_V_1.6^WT^–HA or Na_V_1.6^TAG^–HA (HA positive) did not significantly differ from that of the untransfected (HA negative) neurons (Fig. 4A,B).

Furthermore, we wanted to investigate whether incorporation of TCO*A-Lys into Na_V_1.6 and click labeling had an impact on biophysical properties of the ion channels. To assess this, we performed whole-cell patch clamp recordings of Na^+^ currents in N1E-115-1 cells (Fig. 4C–F; Fig. S7). Cells were co-transfected with TTX-resistant Na_V_1.6^WT,Y371C^–HA or Na_V_1.6^K1546TAG,Y371C^–HA plasmids, NES PylRS^AF^/tRNA^Pyl^, and a multigene plasmid that contained mouse β1 and β2 subunits to ensure full functionality of the Na_V_1.6 channels and GFP to identify transfected cells. The K1546TAG mutation caused a small but significant depolarizing shift (2.8 mV) of the fast inactivation curve, slightly slowed the time course of fast inactivation and accelerated its recovery (Fig. 4C,E; Fig. S7A–E, Table S15). For the Na_V_1.6^K1425TAG,Y371C^–HA variant, the number of transfected cells and the expression level were lower than those of Na_V_1.6^K1546TAG,Y371C^–HA, which corresponded to the reduced Na^+^ currents. To increase the transfection efficiency, we used the commercial PiggyBac transposase system to stably incorporate the mouse β1 and β2 subunits into the genome of the N1E-115-1 cells, reducing the number of plasmids required. To identify the transfected cells, we generated a plasmid containing the genes encoding Na_V_1.6^Y371C^ and enhanced GFP (eGFP), separated by a self-cleaving P2A sequence (Na_V_1.6^Y371C^–P2A–eGFP). Under these conditions, we acquired a sufficient number of transfected cells to record larger Na^+^ currents. The K1425TAG variant had reduced peak current density compared to the WT channel, whereas changes in other gating parameters were not observed (Fig. 4D,F; Fig. S7F–J, Table S15).

### Localization of epilepsy-causing Na_V_1.6 variants with a loss-of-function effect in living primary mouse hippocampal neurons

**Fig. 5.**
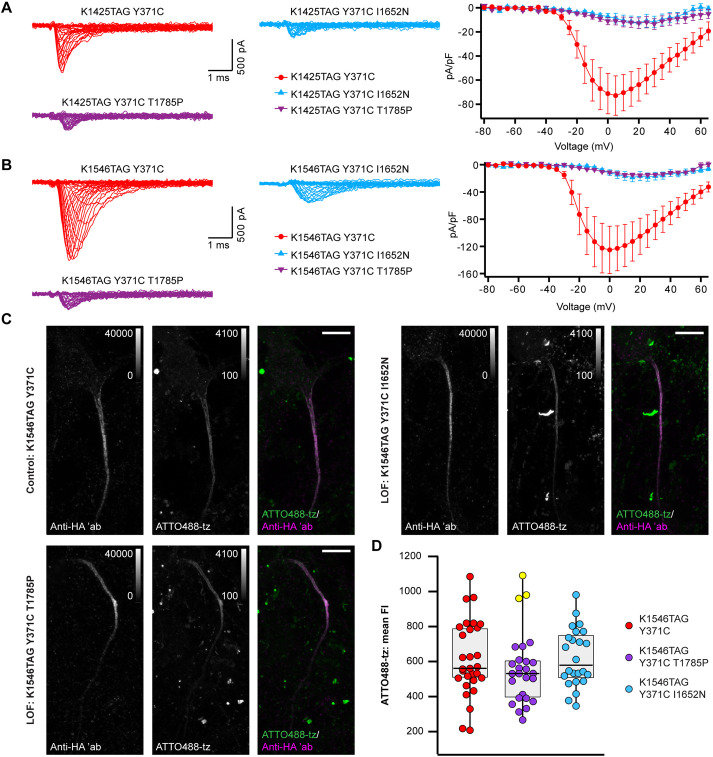
**Click labeling allowed a localization study on the epilepsy-causing Na_V_1.6 variants with loss-of-function effect.** (A,B) Na^+^ current recordings of the indicated clickable mNa_V_1.6 pathogenic variants in N1E-115-1 cells. (A) Representative Na^+^ current traces obtained from N1E-115-1 cells expressing Na_V_1.6^K1425TAG, Y371C^–HA (red), Na_V_1.6 ^K1425TAG,Y371C,I1652N^–HA (blue), or Na_V_1.6 ^K1425TAG,Y371C,T1785P^–HA (violet). Combined with the K1425TAG Y371C mutant, both the I1652N and T1785P variants significantly reduced the peak Na^+^ current density compared to the K1425TAG Y371C mutant alone (Na_V_1.6^K1425TAG, Y371C^–HA: −72.9±16.4 pA/pF, *n*=11; Na_V_1.6^K1425TAG, Y371C, I1652N^–HA: −12.3±5.0 pA/pF, *n*=8, *P*=0.0015; Na_V_1.6 ^K1425TAG,Y371C,T1785P^–HA: −12.7±4.4 pA/pF, *n*=12, *P*=0.0006; ANOVA on ranks with Dunn's post-hoc test). (B) Representative Na^+^ current traces obtained from N1E-115-1 cells expressing Na_V_1.6^K1546TAG,Y371C^–HA (red), Na_V_1.6^K1546TAG, Y371C, I1652N^–HA (blue) or Na_V_1.6^K1546TAG,Y371C,T1785P^–HA (violet). When combined with the K1546TAG Y371C mutant, both the I1652N and T1785P variants significantly reduced the peak Na^+^ current density compared to the K1546TAG Y371C mutant alone (Na_V_1.6^K1546TAG,Y371C^–HA: −125.1±34.8 pA/pF, *n*=13; Na_V_1.6^K1546TAG,Y371C,I1652N^–HA: −18.7±6.3 pA/pF, *n*=8, *P*=0.0018; Na_V_1.6^K1546TAG,Y371C,T1785P^–HA: −15.4±2.0 pA/pF, *n*=8, *P*=0.0014; ANOVA on ranks with Dunn's post-hoc test). Shown are the mean±s.e.m. (C) Representative confocal images of mouse hippocampal neurons at DIV 12 co-expressing NES PylRS^AF^/tRNA^Pyl^ and the mNa_V_1.6 variants indicated. The neurons were labeled with ATTO488-tz, fixed, and immunostained with an anti-HA antibody. The *Z*-stack images are shown as maximum intensity projections. Scale bars: 10 µm. ′ab, primary antibody. (D) Distribution of the mean ATTO488-tz fluorescence intensity (FI) measured in confocal images of click-labeled neurons expressing the indicated mNa_V_1.6 control or LOF variants. The box plots indicate the median (black lines inside the box), the 25th and 75th percentiles (the box boundaries), the single data points (dots), and the outliers (yellow dots). Whisker lengths are defined by the minimum and maximum data points. No significant differences between the groups were detected (*P*>0.05; Kruskal–Wallis test; *n*_K1546TAG Y371C_=29, *n*_K1546TAG Y371C I1652N_=24, and *n*_K1546TAG Y371C T1785P_=26 neurons). Details of the statistical analysis in D are given in Table S16. Data were collected from three (A) or four (B–D) independent experiments.

We next examined whether our click labeling approach in living primary neurons could be used to study the localization of two variants of the human *SCN8A* gene (T1787P and I1654N) that cause a generalized epilepsy. We have recently reported that these two mutations strongly reduced the Na^+^ current density in ND7/23 cells, and reduced firing in mouse hippocampal neuronal cultures compared to the WT channels, indicating a loss-of-function (LOF) effect (Johannesen et al., 2022). However, owing to the lack of suitable labeling approaches, it was unclear whether these mutations affected the function of the Na_V_1.6 channel or its trafficking (thereby leading to reduced Na^+^ current density). To answer these questions, we introduced the corresponding LOF mutations (T1785P and I1652N) into our clickable mouse constructs (mNa_V_1.6^TAG^). When we transfected N1E-115-1 cells with TTX-resistant versions of the mNa_V_1.6^TAG,Y371C^ channels or the mNa_V_1.6^TAG,Y371C^ LOF variants, our results were comparable to those for human (h)Na_V_1.6 (Johannesen et al., 2022), that is, both LOF variants showed strongly reduced Na^+^ currents compared to the mNa_V_1.6^K1425TAG^ or mNa_V_1.6^K1546TAG^ controls (Fig. 5A,B). Hence, we verified that the effect of the hNa_V_1.6 LOF variants was the same for the mNa_V_1.6 that we had generated. These constructs were subsequently transfected into primary neurons, followed by click labeling with cell-impermeable dye to specifically visualize the membrane population of the Na_V_1.6 channels. Because the LOF variants were previously studied in mouse hippocampal neurons (Johannesen et al., 2022), we also used this neuron type for the localization study. We observed that both LOF variants could be labeled extracellularly with click chemistry, suggesting that the channels are expressed in the AIS (Fig. 5C shows representative images for the K1546TAG control and LOF clickable variants). Our quantitative analysis revealed that the AIS fluorescence intensities of both LOF Na_V_1.6 variants did not significantly differ from that of the control (Fig. 5D; Table S16). Therefore, our data suggest that the two mutations causing reduced Na^+^ current density affect the channel conductance or opening probability rather than its trafficking to the membrane.

### AAV-based vectors for delivery of orthogonal translational machinery to primary neurons

The efficiency of transient transfection in terminally differentiated cells such as primary neurons is generally low. This is especially a problem when transfecting multiple plasmids and large genes, such as those used for the engineering of Na_V_1.6 with the UAAs. To overcome this limitation, we developed AAV vectors as tools for delivering NES PylRS^AF^ and tRNA^Pyl^ to primary rat and mouse neurons. Similar AAVs have been previously used in a proof-of-concept study that showed amber codon suppression of a fluorescent reporter protein in mouse neurons (Ernst et al., 2016).

To find a suitable AAV capsid for neuron transduction, we first investigated the engineered variants AAV9A2 and AAV7A2. In previous work, we generated these variants by inserting the small peptide NYSRGVD (called A2) into exposed regions on the capsids of the AAV serotypes AAV7 and AAV9, and found that they efficiently transduced a wide variety of cell types (Borner et al., 2020). In this study, we transduced primary neurons with AAV9A2 or AAV7A2 bearing a fluorescent reporter that consisted of a nuclear localization signal (NLS)–mCherry and GFP^Y39TAG^ fusion. We observed that neurons that had been transduced with the AAV9A2 variant started producing mCherry earlier and at slightly higher levels than those transduced with AAV7A2. We then co-transduced neurons with AAV9A2 that carried the NLS–mCherry–GFP^Y39TAG^ fluorescent reporter and with AAV9A2 carrying different combinations of promoters and genes for UAA incorporation. The promoters and genes tested (Fig. S8A) included multiple copies of tRNA^Pyl^ and the mutant eRF1^E55D^ elongation factor (Schmied et al., 2014), which have been previously used to increase the efficiency of amber codon suppression, as well as conventional and minimal CMV promoters for the expression of NES PylRS^AF^ and eRF1^E55D^. These experiments revealed that a high number of neurons expressed both NLS–mCherry and GFP^Y39TAG^ under all the conditions tested (Fig. S8B), confirming that our AAVs can be used for amber codon suppression of a reporter fluorescent protein.

**Fig. 6.**
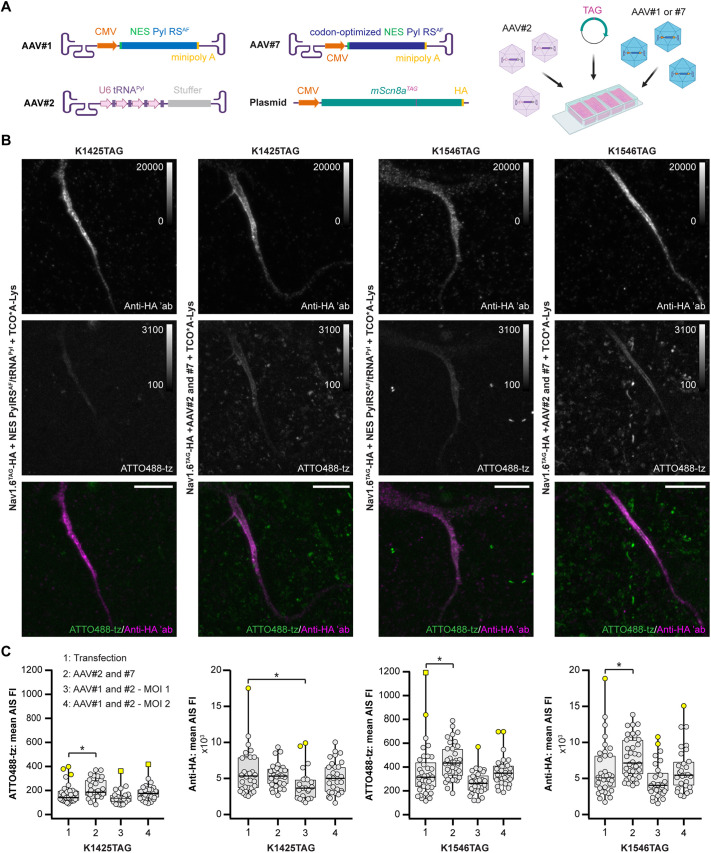
**AAV-based vectors are efficient tools for delivering orthogonal translational machinery for click labeling of Na_V_1.6 in primary neurons.** (A) Schematic representation of AAV9A2 viral vectors and Na_V_1.6–HA plasmid used for genetic code expansion and click labeling of Na_V_1.6. In addition to transfections with Na_V_1.6–HA constructs, neurons were either transfected with NES PylRS^AF^/tRNA^Pyl^ or transduced with orthogonal translational machinery components (AAV#1, AAV#2 and AAV#7). The schemes were created with BioRender.com. (B) Representative confocal images of neurons at DIV 11 expressing the constructs indicated. Prior to imaging, living neurons were labeled with ATTO488-tz, fixed, and immunostained with an anti-HA antibody. The *Z*-stack images are shown as maximum intensity projections. The brightness and contrast of the panels were linearly adjusted as indicated by the LUT intensity scale bar. LUT intensity scale bars show the minimum and maximum gray values. Scale bars: 10 µm. ′ab, primary antibody. (C) Distribution of the mean ATTO488-tz or anti-HA fluorescence intensity (FI) measured in confocal images of transfected or transduced neurons. Significant differences are indicated (*P<0.05, Mann–Whitney *U* test; *n*_K1425TAG-1_=30, *n*_K1546TAG-1_=35, *n*_K1425TAG-2_=28, *n*_K1546TAG-2_=38, *n*_K1425TAG-3_=22, *n*_K1546TAG-3_=28, *n*_K1425TAG-4_=29, *n*_K1546TAG-4_=29 neurons). All box plots indicate the median (black lines inside the box), the 25th and 75th percentiles (box boundaries), the single data points (dots), the outliers (yellow dots) and the furthest outliers (yellow squares). Whisker lengths are defined by the minimum and maximum data points. Dots represent individual neurons. Details of the statistical analysis are given in Tables S17–20. Data were collected from three independent experiments.

We then attempted to use these AAV vectors for click labeling of Na_V_1.6. Given that the AAVs have a limited packaging capacity, it is not possible to pack large mouse *Scn8a* genes into an AAV. Instead, for our purposes, we combined transfection of the Na_V_1.6^TAG^ plasmids and transduction with the AAV9A2 vectors that carried components for genetic code expansion. Similar to the experiments with the mCherry–GFP^Y39TAG^ reporter (Fig. S8), we transduced neurons with combinations of AAV9A2 vectors that carried different orthogonal translational machinery genes. Whereas all of the tested combinations resulted in successful click labeling, a combination of AAVs carrying NES PylRS^AF^ and four copies of tRNA^Pyl^ (AAV#1 and AAV#2, respectively, Fig. 6A) showed the lowest background (data not shown). Our experiments also revealed that the number of neurons that expressed clickable Na_V_1.6 was higher when using transduction compared to only using transfection (50 transduced neurons versus ∼5–15 transfected per well of an eight-well Lab-Tek II chambered cover glass). However, when we repeated these experiments several months later, the potency of AAV#1 dropped and the number of transduced neurons was lower than previously. Thus, we tested higher amount of virus, included an additional AAV variant with codon-optimized NES PylRS^AF^ that we generated meanwhile (AAV#7, Fig. 6A,B), and quantified the average fluorescence intensities of ATTO488 and anti-HA signals. Increasing the amount of AAV#1 increased the efficiency, which is consistent with our hypothesis of its reduced potency. Finally, the use of AAV#7 led to a significant increase in both the expression and the labeling efficiency of Na_V_1.6^K1546TAG^, and had an effect on the level of click labeling for Na_V_1.6^K1425TAG^ (Fig. 6C). In summary, we developed AAV viral vectors that enabled more efficient genetic code expansion and click labeling of Na_V_1.6 in primary neurons.

### dSTORM imaging and ultrastructural analysis of click-labeled Na_V_1.6

**Fig. 7.**
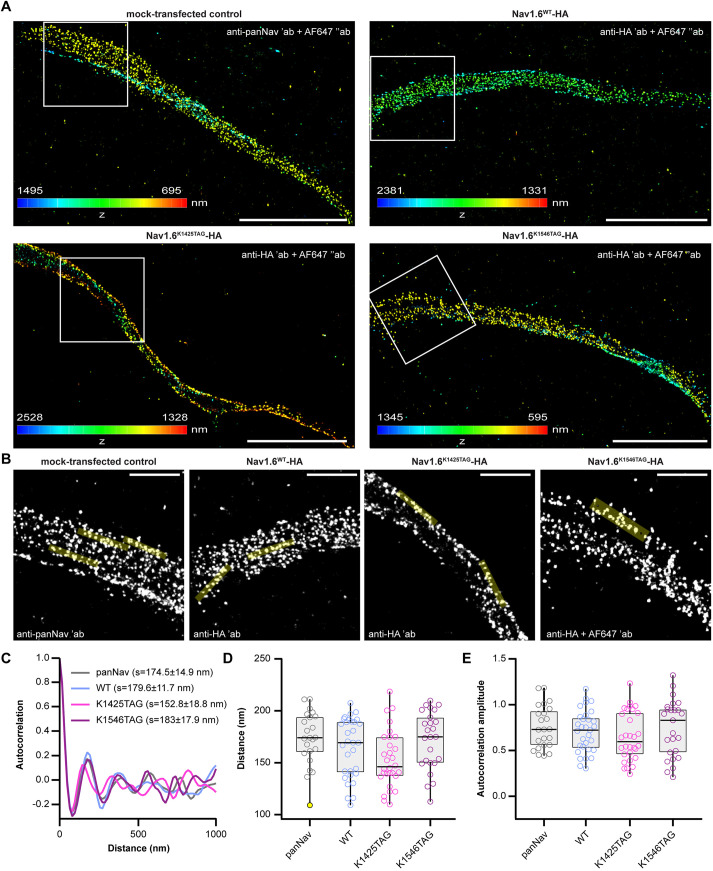
**Quantification of AIS periodicity in neurons expressing Na_V_1.6^WT^–HA** or **Na_V_1.6^TAG^–HA.** (A) Representative 3D dSTORM images of primary rat cortical neurons at DIV 12 expressing the indicated constructs and immunostained with the indicated antibodies. The *z* positions are color-coded according to the height maps (bottom-left). The height maps contain minimal and maximal *z* position values. (B) Boxed regions from A with representative overlaid line ROIs that were used for the autocorrelation analysis. ′ab, primary antibody; ″ab, secondary antibody. (C) The averaged autocorrelation curves from 1-µm-long intensity profiles along axons expressing constructs indicated (*n*_panNav_=31, *n*_WT_=23, *n*_K1425TAG_=30, *n*_K1546TAG_=25 individual ROIs measured from *n*_panNav_=12, *n*_WT_=11, *n*_K1425TAG_=15, *n*_K1546TAG_=11 neurons). Mean±s.e.m. spacing values (*s*) are indicated in the graph. (D,E) Spacing (D) and autocorrelation amplitude (E) values of individual ROIs for indicated constructs. No significant differences between the groups were detected [*P*>0.05, Kruskal–Wallis test followed by Dunn post-hoc analysis with Bonferroni correction for multiple comparisons (D) or one-way ANOVA with Tukey's post-hoc analysis (E); *n*_panNav_=31, *n*_WT_=22, *n*_K1425TAG_=30, *n*_K1546TAG_=26 individual ROIs measured from *n*_panNav_=12, *n*_WT_=11, *n*_K1425TAG_=15, *n*_K1546TAG_=11 neurons]. All box plots indicate the median (black lines inside the box), the 25th and 75th percentiles (box boundaries), the single data points (dots) and outliers (yellow dots). Whisker lengths are defined by the minimum and maximum data points. Dots represent individual axonal regions (line ROIs). Details of the statistical analysis are given in Tables S21, S22. Data were collected from three independent experiments. Scale bars: 5 μm (A), 1 μm (B).

**Fig. 8.**
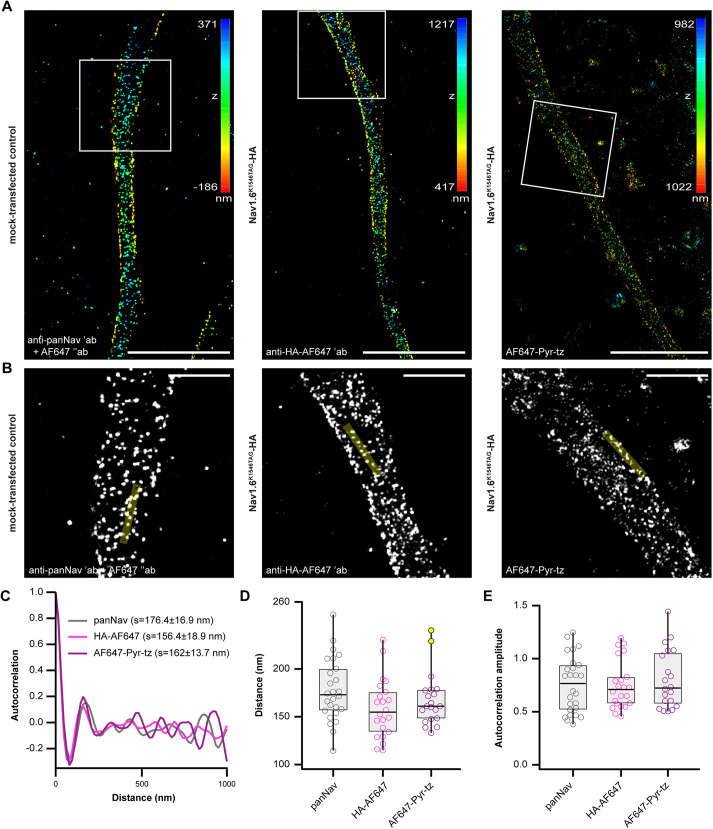
**Quantification of the Na_V_1.6–HA AIS periodicity in click-labeled neurons.** (A) Representative 3D dSTORM images of click-labeled primary rat cortical neurons at DIV 12 expressing Na_V_1.6^K1546TAG^–HA or mock-transfected control. From left to right, images were taken in panNa_V_, AF647-conjugated HA and AF647-pyr-tz channels. The *z* positions are color-coded according to the height maps (right). The height maps contain minimal and maximal *z* position values. (B) Boxed regions from A with representative overlaid line ROIs that were used for the autocorrelation analysis. (C) The averaged autocorrelation curves from 1-μm-long intensity profiles along axons (*n*_panNav_=26, *n*_HA-AF647_=22, *n*_AF647-pyr-tz_=20 ROIs measured from *n*_panNav_=14, *n*_HA-AF647_=12, *n*_AF647-pyr-tz_=11 neurons). Mean±s.e.m. spacing values (*s*) are indicated in the graph. (D,E) Spacing (D) and autocorrelation amplitude (E) values of individual ROIs for indicated constructs. No significant differences between the groups were detected (*P*>0.05, Kruskal–Wallis test; *n*_panNav_=26, *n*_HA-AF647_=22, *n*_AF647-pyr-tz_=20 individual ROIs collected from *n*_panNav_=14, *n*_HA-AF647_=12, *n*_AF647-pyr-tz_=11 neurons). All box plots indicate the median (black lines inside the box), the 25th and 75th percentiles (box boundaries), the single data points (dots) and outliers (yellow dots). Whisker lengths are defined by the minimum and maximum data points. Dots represent individual axonal regions (ROIs). Details of the statistical analysis are given in Tables S23, S24. Data were collected from two independent experiments. Scale bars: 5 μm (A), 1 μm (B).

Next, we performed dSTORM on click-labeled neurons (Figs 7 and 8) to investigate whether the nanoscale organization of the Na_V_1.6 was affected by the expression of recombinant Na_V_1.6 or the click labeling. First, we performed conventional immunofluorescence (Fig. 7A) in order to compare the degree of periodicity and spacing of Nav1.6^WT^ or Nav1.6^TAG^ in primary neurons. For the immunostaining, we used anti-HA primary antibody and the dSTORM-compatible AF647-conjugated secondary antibody. Immunostaining of the HA tag fused to WT or click-labeled Na_V_1.6^TAG^ channels allowed us to directly compare them and investigate the effect of UAA incorporation and click labeling on AIS structure. In addition, we included mock-transfected neurons that were stained with a pan-Na^+^ channel (panNa_v_) antibody. Autocorrelation analysis (Fig. 7B–E) revealed no significant differences between the spacing (Fig. 7D) and the degree of periodicity (Fig. 7E) of endogenous panNa_V_ staining and recombinant Na_V_1.6^WT^–HA and Na_V_1.6^TAG^–HA.

Finally, in analogous experiments to those with NF186 (Fig. 2H–L), we investigated whether click labeling of Na_V_1.6 was compatible with dSTORM (Fig. 8). After we obtained dSTORM images (Fig. 8A), we analyzed Na_V_ periodicity in mock-transfected neurons (immunostained with panNa_V_) and neurons transfected with Na_V_1.6^K1546TAG^–HA. Autocorrelation analysis (Fig. 8B–E) revealed no significant difference between the spacing (Fig. 8D) and the degree of periodicity (Fig. 8E) of the endogenous panNa_V_ staining and Na_V_1.6^K1546TAG^–HA in click and HA channels. Together, this suggests that our click-labeled constructs do not affect the nanoscale organization of the AIS and that they are suitable for super-resolution imaging studies.

## DISCUSSION

The AIS is organized in a highly complex and unique way, and studying its components using live-cell and super-resolution microscopy requires the method of labeling to be chosen with care. In this study, we combined genetic code expansion and bioorthogonal click chemistry to perform fluorescent labeling of two AIS components – the cell adhesion molecule NF186 and the voltage-gated ion channel Na_V_1.6. The main advantage of our labeling approach is that a small fluorescent dye is directly attached to the proteins of interest in living neurons. Importantly, the dye–UAA product formed by click chemistry is much smaller (∼0.5–2 kDa) than fluorescent proteins (∼30 kDa) and antibodies (∼150 kDa). Therefore, the protein of interest is modified in a minimally invasive way. Given that the UAAs are introduced site-specifically into the protein of interest, positions and domains that are important for the function of the target protein can be avoided. This is particularly important for the labeling of Na_V_, in which a single mutation can severely impair the function of the channel (Johannesen et al., 2022; Meisler et al., 2021; Sole and Tamkun, 2020). For these reasons, click chemistry represents a powerful approach for live-cell labeling of AIS components.

Although click labeling has been used for other smaller (35–65 kDa) neuronal proteins and cytoskeleton (Arsić et al., 2022; Bessa-Neto et al., 2021), NF186 and Na_V_1.6 are challenging targets to label owing to their large size and spatially restricted expression at the AIS. For technical reasons, we first established click labeling of the smaller AIS component, NF186 (186 kDa). Labeling of Na_V_1.6 was more challenging owing to its complex structure and larger size (∼260 kDa). We unsuccessfully attempted click labeling of Na_V_1.6 in two cell lines before we were able to establish it in primary neurons. Although counterintuitive at first, using the more complex native environment was crucial for this achievement. For the labeling, we probed two positions in extracellular loops of Na_V_1.6 – K1546 and K1425. Akin et al. (2015) have reported that incorporation of the 17-amino-acid-long BAD domain at position K1546 only minimally affects the function of Na_V_1.6. They showed that the current density measured in rat neurons was reduced, whereas other biophysical parameters measured in ND7/23 cells were unaffected. In the present study, we observed that both clickable variants reduced the current density, although this reduction was statistically significant only in the case of Na_V_1.6^K1425TAG^. The reduction in the current density of Na_V_1.6^TAG^ is unsurprising because suppression of amber codons is not 100% efficient (Arsić et al., 2022; Bartoschek et al., 2021; Nikic-Spiegel, 2020). In addition, we observed a small shift of 2.8 mV in the inactivation curve of the Na_V_1.6^K1546TAG^, whereas we did not observe any changes in the biophysical properties of the Na_V_1.6^K1425TAG^. However, despite our attempts to increase its expression level, the click labeling efficiency of Na_V_1.6^K1425TAG^ was lower than that of Na_V_1.6^K1425TAG^, most likely due to the UAA being less accessible to the tetrazine dye. This is supported by the AlphaFold-predicted 3D structure analysis that became available during the course of the study (Jumper et al., 2021; Varadi et al., 2021). Hence, we continued to use the K1546TAG mutant. The AAV vectors increased the expression and click labeling efficiencies of Na_V_1.6^K1546TAG^ and generally resulted in a higher number of click-labeled neurons. However, it is important to address their limitations. Owing to the limited packaging capacity (∼5 kb) of AAVs, we used multiple AAVs to deliver different components of the orthogonal translational machinery. We are currently attempting to overcome this limitation by testing AAV variants with minimal promoters that allow all the necessary components to be packaged into one AAV. Furthermore, we encountered problems of potency drop with some of our AAV variants. Although we initially even managed to perform dSTORM imaging of the neurons transduced with AAV#1 and AAV#2, in subsequent experiments this combination was less potent. This was most likely caused by suboptimal and prolonged storage and care should be taken to avoid any fluctuations in this regard. Moreover, the large mouse *Scn8a* gene cannot be packed into an AAV. To avoid having to combine plasmid transfection and AAV transduction, baculoviruses that have high packaging capacity could be used instead. Such vectors have been used previously for genetic code expansion of a fluorescent reporter protein in cells and *ex vivo* mouse brain slice cultures (Chatterjee et al., 2013; Zheng et al., 2017).

In summary, we developed a minimally invasive approach for labeling NF186, as well as WT and epilepsy-causing pathogenic Na_V_1.6 channels, with small fluorescent dyes in living neurons. UAA-based minimal tags offer the opportunity to study localization and trafficking of NF186 and Na_V_1.6 in developing, mature, healthy or injured neurons. Thanks to its compatibility with live-cell imaging, this labeling approach will provide new insights into the dynamics and plasticity of these proteins. Furthermore, the combination of two different tetrazine dyes (Arsić et al., 2022) or two different click reactions (Nikic et al., 2014) in a pulse−chase manner could be used to study different populations of NF186 or Na_V_. Moreover, in addition to conventional imaging, the small size of the labeling tag and the variety of available tetrazine dyes make click labeling particularly suitable for super-resolution imaging techniques, such as dSTORM or stimulated emission depletion microscopy (Arsić et al., 2022; Bessa-Neto et al., 2021). In this study, we performed dSTORM of both click-labeled NF186 and Na_V_1.6. In line with the requirements of dSTORM imaging, we focused on imaging the brightest click-labeled neurons. Owing to higher expression and labeling levels of recombinant NF186, dSTORM of click-labeled neurons was slightly more feasible for NF186 than Na_V_1.6. Furthermore, in agreement with the published literature showing less-pronounced periodicity of endogenous immunostained Na_V_ channels and NF186 compared to other AIS components (D'Este et al., 2017; Leterrier et al., 2015; Xu et al., 2013), we noticed patches of periodic patterns and incomplete periodicity. This might also reflect the fact that, in our study, click-labeled NF186 and Na_V_1.6 were overexpressed in the presence of endogenous proteins and thus represented only a subset of proteins at the AIS. Incomplete periodicity can also be a consequence of variable expression levels in individual neurons, maturation stage (days *in vitro*; DIV) of imaged neurons, fixation and immunostaining protocols (Tian et al., 2014). However, importantly, our imaging of the panNF- and panNa_V_-immunostained control neurons showed no difference between the spacing and periodicity of the endogenous and click-labeled proteins under the immunostaining and imaging conditions tested in our work.

We also showed that our labeling approach could be used not only for different AIS components and different imaging approaches but also across different neuronal types (i.e. mouse hippocampal neurons and rat cortical neurons), thereby strengthening the generality of this method. Furthermore, this approach can be easily adjusted for other neurofascin and Na_V_ isoforms, including different disease-associated variants. This method could also be established for labeling other AIS and nodes of Ranvier proteins, including ion channels, for which antibodies, fluorescent protein fusions and other labeling tags cannot be used.

Finally, we developed AAV-based viral vectors to more efficiently deliver the components required for genetic code expansion into primary neurons. Together with the availability of natural or synthetic AAV capsid variants as well as hybrid parvoviral vectors with different cell-type specificities (Fakhiri and Grimm, 2021; Grimm and Zolotukhin, 2015), this will facilitate click-chemistry-based protein engineering of more complex systems, such as organotypic slice cultures, organoids, and animal models.

## MATERIALS AND METHODS

### Plasmids, cloning and mutagenesis, including selection of UAA incorporation sites

For the click labeling of NF186, we used a plasmid that contained a rat *Nfasc* gene with an HA tag at the C terminus (NF186–HA). The C-terminal HA tag allowed us to detect the full-length NF186 protein by immunostaining with an anti-HA antibody. The NF186–HA construct was generated from a plasmid that contained a WT rat *Nfasc* gene expressed from the CMV promoter (Addgene plasmid #31061; deposited by Vann Bennett; Zhang et al., 1998) by moving the HA tag from the N- to the C-terminus. To delete the HA tag from the N-terminus, we used the QuikChange II XL Site-Directed Mutagenesis Kit (Agilent Technologies, cat. no. 200522) according to the manufacturer's instructions. In the resulting construct, the HA tag was added to the C-terminus via PCR-mediated cloning using the ApaI and NotI restriction sites (resulting plasmid: CMV-NF186^WT^–HA). Clickable NF186–HA mutants (CMV-NF186^TAG^–HA) were generated by introducing amber stop codons (TAG) into the *Nfasc* gene of the original Addgene plasmid at positions K534 and K680, or by modifying CMV-NF186^WT^–HA by introducing TAG codons into the *Nfasc* gene at positions K519, K571, K604 or K809. All modifications were introduced by PCR-based site-directed mutagenesis. In the experiments with ND7/23 cells, CMV-NF186^WT^–HA or CMV-NF186^TAG^–HA was used. For the click labeling of NF186 in primary neurons, the CMV promoter was excised from CMV-NF186^WT^–HA and six CMV-NF186^TAG^–HA plasmids and replaced with the hNSE promoter by using AseI and BgIII restriction sites. The hNSE promoter was amplified from the pGL3 NSE plasmid (Addgene plasmid #11606; deposited by Rosalyn Adam; Kim et al., 2004). We used One Shot TOP10 Electrocompetent *Escherichia coli* (Thermo Fisher Scientific, cat. no. C40452) in all the experiments that involved mutagenesis, cloning, and amplification of the NF186 plasmids, except for the experiment involving deletion of the HA tag, for which we used the XL 10-Gold Ultracompetent Cells (Agilent, cat. no. 200315) provided with the mutagenesis kit.

Mouse 654-bp-long *Scn1b* (*mScn1b*, Clone ID: OMu07915D ORF clone, accession no. NM:011322.2) and mouse 558-bp-long *Scn2b* (*mScn2b*, clone ID: Omu42415D ORF clone, accession no. XM:006510629.3 ORF sequence) cDNAs cloned into the pcDNA3.1+/C-(k)-DYK vectors were obtained from GenScript. For the electrophysiological measurement of Na^+^ currents, we made a multigene plasmid that contained monomeric eGFP and the *mScn1b* and *mScn2b* genes using the MultiBacMam system kit (Geneva Biotech). We first cloned *mScn1b* into the pACEMam2 acceptor vector (pACEMam2 β1) and *mScn2b* into the pMDS donor vector (pMDS β2) using NheI and KpnI restriction sites, and then we cloned eGFP into the pMDC donor vector (pMDC eGFP) using BamHI and Xbal restriction sites. The donor and acceptor vectors were components of the MultiBacMam system kit, and cDNA encoding for eGFP was amplified from the eGFP-N1 plasmid (Addgene plasmid #54767; deposited by Michael Davidson). The multigene construct (pACEMam2_mβ1_mβ1_eGFP) was made via CreLox recombination between donor vectors and an acceptor vector, according to the manufacturer's instructions. The donor vectors were propagated in pirHC cells (Geneva Biotech); all other plasmids were propagated in One Shot TOP10 Electrocompetent *E. coli*. For the generation of the N1E-115^β1β2^ stable cell lines, we cloned *mScn1b* or *mScn2b* into the PiggyBac PB-CMV-MCS-EF1α-Puro cDNA/miRNA cloning and expression vector (BioCat, cat. no. PB510B-1-SBI) using NheI and NotI restriction sites (PiggyBac mβ1, PiggyBac mβ2).

Mouse voltage-gated Na^+^ channel 5934-bp genes encoding Na_V_1.6 (*mScn8a*^WT^, *mScn8a*^K1425TAG^, *mScn8a*^K1546TAG^) were synthesized by GenScript (*mScn8a*; transcript variant 1, NCBI Reference Sequence: NM_001077499.2).

Selecting the site at which to incorporate an UAA into Na_V_1.6 presented additional challenges compared to NF186. Firstly, there are many disease-related mutations in the *SCN8A* gene (Johannesen et al., 2022; Meisler et al., 2021), and there are many conserved regions of Na_V_1.6 that are crucial for the function of the channel (e.g. the S4 segments of domains I–IV and pore-forming loops; Fig. 3A) (Catterall et al., 2005). These positions and domains had to be avoided when selecting TAG positions to ensure that the function of the Na_V_1.6 would not be affected. Furthermore, a crystal structure of Na_V_1.6 is unavailable, and the α-subunit of the channel is heavily glycosylated, making it harder to select positions for the TAG codon that would place the UAA in a site accessible to the tetrazine dye. The chosen positions (K1425 and K1546) are located in the extracellular loops and in less-conserved regions of the channel. Furthermore, these residues do not participate in forming the pore, opening the channel or regulating its function, and are not known to be associated with any disease. In addition, although the method is not limited to lysine residues, this can be of advantage because lysine residues share structural similarity with our lysine-derived UAA.

The synthesized *mScn8a* genes were cloned into pcDNA3.1-P2A-eGFP vectors using EcoRI and Xbal restriction sites. The final plasmids contained the *mScn8a^WT^* or *mScn8a^TAG^* genes, followed by the Xbal restriction site, a self-cleaving (22 amino acids) P2A sequence, eGFP and a TAA stop codon (Na_V_1.6–P2A–eGFP). For establishing click labeling of Na_V_1.6, P2A–eGFP was excised and replaced with the HA tag using Apal and XbalI restriction sites (Table S27). In the final plasmids, the open reading frame (ORF) contained the *mScn8a* gene, followed by the HA tag and a TAA stop codon (Na_V_1.6–HA). The HA tag oligonucleotide strand was synthesized by Sigma-Aldrich as two complementary single-stranded oligonucleotides (Table S27). For the electrophysiological measurement of Na^+^ currents, the Na_V_1.6–P2A–eGFP and Na_V_1.6–HA plasmids were rendered TTX resistant by introducing the previously described Y371C point mutation into the *mScn8a*^WT^ and *mScn8a*^TAG^ genes (Leffler et al., 2005; Liu et al., 2019). To study the localization of the LOF mNa_V_1.6–HA variants, we introduced I1652N or T1785P mutations (Johannesen et al., 2022) into the *mScn8a*^WT,Y371C^, *mScn8a*^K1425TAG,Y371C^, and *mScn8a*^K1546TAG,Y371C^ genes. Mutagenesis of *mScn8a* was performed using the QuikChange II XL site-directed mutagenesis kit. In the experiments involving cloning, mutagenesis and amplification of *mScn8a*, we used chemically competent XL-10 Gold Ultracompetent cells. All the steps involving propagation of Na_V_1.6 in bacteria were performed at 27–28°C (Feldman and Lossin, 2014; O'Brien and Meisler, 2013) to avoid the introduction of additional mutations and rearrangements of *mScn8a.*

For the incorporation of TCO*A-Lys into NF186^TAG^–HA and Na_V_1.6^TAG^–HA, we used a recently described pcDNA3.1/Zeo(+) plasmid that contained the codon-optimized NES PylRS^AF^ and its cognate amber codon suppressor tRNA^Pyl^ (Addgene plasmid #182287; Arsić et al., 2022). For electrophysiological measurement of Na^+^ currents, we transfected N1E-115-1 cells with the codon-optimized NES PylRS^AF^/tRNA^Pyl^ pair and WT or K1546TAG Na_V_1.6 plasmids, whereas we transfected the N1E-115-1^β1β2^ cells with NES PylRS^AF^/tRNA^Pyl^ – a gift from Dr Edward Lemke (IMB, Mainz, Germany) – and WT or the K1425TAG Na_V_1.6 plasmids. For mock-transfected conditions, we used an empty pcDNA3.1/Zeo(+) plasmid as a control (gift from Dr Edward Lemke).

All of the modifications introduced into the abovementioned plasmids were confirmed by sequencing. For modified Na_V_1.6, the entire *mScn8a* ORFs were sequenced to confirm that there were no additional mutations or rearrangements prior to transfection. The mutagenesis primers, cloning primers, and oligonucleotide sequences are provided in Table S27.

Plasmids generated in this study are available upon request from the corresponding author or will be made available on Addgene.

### UAAs, tetrazine-dye derivatives and antibodies

We used the UAA *trans*-cyclooct-2-en-L-lysine (TCO*A-Lys; SICHEM, Bremen, Germany, cat. no. SC-8008). A 100 mM stock solution of TCO*A-Lys in 0.2 M NaOH and 15% DMSO was diluted 1:4 in 1 M HEPES (Thermo Fisher Scientific, cat. no. 15630056) and added to the medium at a final concentration of 250 µM. For the click labeling of NF186–HA and Na_V_1.6–HA, we used ATTO488-tetrazine (Jena Bioscience, cat. no. CLK-010-02) and AF647-pyrimidyl-tetrazine (Jena Bioscience, cat. no. CLK-102). Stock solutions of ATTO488-tz and AF647-pyr-tz (500 and 1250 µM, respectively, in DMSO) were diluted in warm culture medium at final concentrations of 1.5, 3 or 5 µM (ATTO488-tz) and 10 µM (AF647-pyr-tz).

The primary antibodies used were as follows: mouse anti-HA tag (2-2.2.14) antibody (1:1000; Thermo Fisher Scientific, cat. no. 26183) was used for click labeling of NF186–HA in ND7/23 cell line; rabbit anti-HA tag (C29F4) monoclonal antibody [1:1000 for click labeling of Na_V_1.6 (except for experiments involving quantification of dSTORM in which it was used at 1:250 dilution) and 1:2000 for click labeling of NF186 in primary neurons; Cell Signaling Technology, cat. no. 3724]; rabbit anti-HA tag (SG77) polyclonal antibody (1:1000 for western blot analysis; Thermo Fisher Scientific, cat. no. 71-5500); mouse anti-ankyrin G antibody (1:50; Santa Cruz Biotechnology, cat. no. 12719); mouse anti-ankyrin G (N106/36) monoclonal antibody (1:100; Neuromab, cat. no. 75-146); rabbit anti-panNF antibody (1:200; Abcam, cat. no. ab31457); mouse anti-panNa_V_ (K58/35) monoclonal antibody (1:100; Sigma-Aldrich, cat. no. S8809); mouse anti-βIII-tubulin monoclonal antibody (1:1000; BioLegend, cat. no. 801202); AF647-conjugated anti-HA tag (C29F4) rabbit antibody (1:50 for Na_V_1.6 and 1:100 for NF186; Cell Signaling Technology, cat. no. 37297). The secondary antibodies used were as follows: goat anti-rabbit-IgG conjugated to AF555 (1:500; Thermo Fisher Scientific, cat. no. A21429); goat anti-rabbit-IgG conjugated to AF647 Plus [1:500; AF(+)647; Thermo Fisher Scientific, cat. no. A32733]; goat anti-mouse-IgG conjugated to AF633 (1:500; Thermo Fisher Scientific, cat. no. A-21052); goat anti-mouse-IgG conjugated to AF(+)647 (1:500; Thermo Fisher Scientific, cat. no. A32728); goat anti-rabbit-IgG conjugated to horseradish peroxidase (HRP; Thermo Fisher Scientific, cat. no. A16104); goat anti-mouse-IgG conjugated to HRP (Thermo Fisher Scientific, cat. no. A16072) polyclonal antibodies.

All antibodies were obtained commercially and validated by the data sheets of the manufacturer or via citations listed on the manufacturer's website.

### Cell culture

Mouse neuroblastoma×rat neuron hybrid ND7/23 cells (ECACC 92090903, Sigma-Aldrich) were grown in high-glucose Dulbecco's modified Eagle's medium (DMEM; Thermo Fisher Scientific, cat. no. 41965062) supplemented with 10% heat-inactivated fetal bovine serum (FBS; Thermo Fisher Scientific, cat. no. 10270106), 1% penicillin–streptomycin (PS; Sigma-Aldrich, cat. no. P0781), 1% sodium pyruvate (Thermo Fisher Scientific, cat. no. 11360039) and 1% L-glutamine (Thermo Fisher Scientific, cat. no. 25030024) at 37°C and 5% CO_2_. FBS was heat-inactivated by incubation at 56°C for 30 min. Mouse neuroblastoma N1E-115-1 cells (ECACC 08062511, Sigma-Aldrich) were grown in high-glucose DMEM supplemented with 10% FBS and 1% PS at 37°C and 5% CO_2_. For maintenance of the PiggyBac N1E-115-1^β1β2^ stable cells, in addition to 10% FBS and 1% PS we supplemented the medium with 3 µg/ml puromycin (Sigma-Aldrich, cat. no. P8833). For experiments involving transfection and electrophysiological recordings, neuronal cells were passaged three times a week and used at passages 3–15. ND7/23 and N1E-115-1 cell lines were authenticated and confirmed negative for mycoplasma contamination by their providers. In addition, ND7/23 and N1E-115-1^β1β2^ stable cells propagated in our laboratory were confirmed negative for mycoplasma contamination (Eurofins Genomics).

For the microscopy experiments, neuroblastoma ND7/23 or N1E-115-1 cells were seeded on four-well Lab-Tek II chambered cover glasses (German #1.5 borosilicate glass; Thermo Fisher Scientific, cat. no. 155382) at the following densities: 50,000 ND7/23 cells per well for click labeling of NF186 and 100,000 ND7/23 cells per well or 60,000 N1E-115-1 cells per well for click labeling of Na_V_1.6. Before cell seeding, chambered cover glasses were pre-coated with 10 µg/ml poly-D-lysine (PDL; Sigma-Aldrich, cat. no. P6407) solution in double-distilled water (ddH_2_O) for at least 4 h at room temperature (RT). The chambered cover glasses were washed three times with ddH_2_O and dried completely before cell seeding. For the electrophysiological recordings, 250,000 N1E-115-1 or 160,000–200,000 PiggyBac N1E-115-1^β1β2^ stable cells were seeded per well of a six-well plate the day prior to transfection.

For the experiments that included genetic code expansion and click labeling of NF186 and Na_V_1.6, Gibco primary rat cortex neurons from Sprague–Dawley embryonic-day-18 rats (Thermo Fisher Scientific, cat. no. A36512) were thawed and cultured based on the supplier's recommendations. The neurons were maintained in the B-27 Plus Neuronal Culture System (Thermo Fisher Scientific, cat. no. A3653401), which contained 2% B27 Plus and 1% PS. For the microscopy experiments, rat neurons were seeded in eight-well Lab-Tek II chambered cover glasses (German #1.5 borosilicate glass; Thermo Fisher Scientific, cat. no. 155409) at a density of 100,000–120,000 neurons per well. Prior to neuron seeding, the chambered cover glasses were pre-coated with a 20 µg/ml solution of PDL in ddH_2_O (Sigma-Aldrich, cat. no. P6407 or Thermo Fisher Scientific, cat. no. A3890401) for 2 h at RT. Afterwards, they were washed three times with ddH_2_O, dried completely and pre-incubated with 250 µl of warm culture medium for at least 30 min at 37°C, 5% CO_2_. After seeding, half of the culture medium was replaced with fresh medium every 3–4 days.

For the click labeling of LOF Na_V_1.6 variants, we used mouse hippocampal neurons. Our animal protocols for the mouse hippocampal neuronal culture preparation were approved by the local Animal Care and Use Committee (Regierungspraesidium Tübingen, Tübingen, Germany). Neurons were isolated from embryonic-day-18 C57BL/6NCrl mouse pups of mixed sex. The pregnant mice were cervically dislocated after asphyxiation with CO_2_, then the embryos were taken out and decapitated immediately. The brains were stored in cold Mg^2+^- and Ca^2+^-free Hanks' balanced salt solution (HBSS; Thermo Fisher Scientific, cat. no. 14175053) and the hippocampi within the whole brains were identified under a dissecting microscope (Olympus SZ 61, Shinjuku, Tokyo, Japan) and isolated using fine forceps. After the hippocampi were washed three times with cold HBSS solution, the hippocampal tissue was incubated for 14 min in 2.5% trypsin (Thermo Fisher Scientific, cat. no. 15090046) at 37°C and then washed in DMEM containing FBS (PAN Biotech, cat. no. 3306-P131004) to block further enzyme digestion. Dissociated neurons were obtained by gentle mechanical trituration and plated on four-well Lab-Tek II chambered cover glasses at a density of 120,000 neurons per well. The cover glasses were pre-coated with 0.1 mg/ml of PDL solution in ddH_2_O for ∼2 h prior to embryo preparation. After 6 h, during which the neurons settled on the chambered coverslips in a humidified 5% CO_2_ atmosphere at 37°C, the culture medium was replaced by Neurobasal culture medium (Thermo Fisher Scientific, cat. no. 21103049) supplemented with B27 (Thermo Fisher Scientific, cat. no. 17504044), L-glutamine (Thermo Fisher Scientific, cat. no. 25030024) and PS (Thermo Fisher Scientific, cat. no. 15140122). Half of the neuronal culture medium was exchanged for fresh medium every 3–4 days. All experiments involving animals were done according to the local legislations and were approved by the local Animal Care and Use Committee (Regierungspraesidium Tübingen, Tübingen, Germany).

### Generation of stable cell lines

For the generation of N1E-115-1^β1β2^ stable cells, we used the PiggyBac Transposon system (BioCat). Before starting, we made a dose–response curve and assessed that the appropriate puromycin concentration for the selection of stable clones was 3 µg/ml. N1E-115-1 cells were seeded on a six-well plate at a density of 200,000 cells per well the day prior to transfection. Cells were transfected with 0.5 µg PiggyBac mβ1 plasmid, 0.5 µg PiggyBac mβ2 plasmid, and 0.4 µg Super PiggyBac Transposase Expression vector (BioCat, cat. no. PB210PA-1-SBI). We used the JetPrime (Polyplus-transfection, cat. no. 114-15) transfection reagent according to the manufacturer's instructions (2.8 µl of JetPrime transfection reagent per 1.4 µg of DNA). Cells were incubated with the transfection mixture for 4.5 h, after which time the medium was replaced. The day after transfection, selection was initiated by the addition of 3 µg/ml puromycin. To ensure that all the untransfected cells were eliminated, we increased the puromycin concentration for selection from 3 to 6 µg/ml and added it to the cells the day after transfection. At 72 h after transfection, the cells were transferred from the six-well plates to p10 Petri dishes (Greiner Bio-one, cat. no. 664160). The cells were further propagated in p10 Petri dishes and p20 Petri dishes (Greiner Bio-one, cat. no. 639160) until single cell-derived clones of genetically engineered cells formed. Afterwards, we selected single cell-derived clones and propagated them further in p10 dishes. Stocks of various clones were frozen for later analysis. To confirm that *mScn1b* and *mScn2b* were stably incorporated into the genomes of the N1E-115-1 cells, we extracted genomic DNA (gDNA) from different clones using the PureLink gDNA kit (Thermo Fisher Scientific, cat. no. K1820-01) and PCR-amplified *mScn1b* and *mScn2b* genes from the gDNA. The clones with correct patterns on the gel were used in the experiments. The primers used for amplification of gDNA are given in Table S27.

### Transfections

For the click labeling of NF186, ND7/23 cells were transfected using the Lipofectamine 2000 transfection reagent (Thermo Fisher Scientific, cat. no. 11668027) 1 day after seeding, as previously described by us in detail (Arsić et al., 2022 and see protocol at https://doi.org/10.21203/rs.3.pex-1691/v1). In brief, cells were seeded into four-well Lab-Tek II chambered cover glasses and transfected with a total amount of DNA of 1 µg per well (0.5 µg WT or TAG plasmid and 0.5 µg NES PylRS^AF^/tRNA^Pyl^ plasmid) at a DNA/Lipofectamine 2000 ratio of 1 µg/2.4 µl. After the addition of the transfection mixture, TCO*A-Lys in 1 M HEPES was added to the medium at a final concentration of 250 µM. After 6 h, the medium was replaced, TCO*A-Lys was again added at 250 µM, and the cells were incubated overnight. The following day, the cells were click-labeled and immunostained.

For western blot analysis of NF186, 250,000 ND7/23 cells per well were seeded on six-well plates 1 day prior to transfection. Cells were transfected and incubated with TCO*A-Lys as described above with a total amount of DNA of 5 µg per well (2.5 µg CMV-NF186^WT^–HA or CMV-NF186^TAG^–HA and 2.5 µg codon-optimized NES PylRS^AF^/tRNA^Pyl^ plasmids) at a DNA/Lipofectamine 2000 ratio of 1 µg/2.4 µl.

For click labeling of Na_V_1.6, ND7/23 or N1E-115-1 cells were transfected using the Lipofectamine 3000 transfection reagent (Thermo Fisher Scientific, cat. no. L3000015) 1 day after seeding. We used a DNA/Lipofectamine 3000 ratio of 1 µg/1.5 µl and a DNA/P3000 ratio of 1 µg/2 µl. For the microscopy experiments, ND7/23 or N1E-115-1 cells were seeded into four-well Lab-Tek II chambered cover glasses and transfected using a total of 1.8 µg of DNA per well (0.8 µg Na_V_1.6 WT or TAG, 0.8 µg NES PylRS^AF^/tRNA^Pyl^, 0.1 µg pACEMam mβ1 and 0.1 µg pMDC mβ2). For the electrophysiological experiments, N1E-115-1 cells were seeded onto six-well plates and transfected with a total amount of 8.4 µg DNA per well (4 µg Na_V_1.6, 4 µg NES PylRS^AF^/tRNA^Pyl^, and 0.4 µg pACEMam2_mβ1_mβ2_eGFP). N1E-115-1^β1β2^ cells were seeded onto six-well plates and transfected with a total amount of 5 µg of DNA per well (2.5 µg Na_V_1.6–P2A–eGFP and 2.5 µg NES PylRS^AF^/tRNA^Pyl^). After the transfection mixture was added, TCO*A-Lys in 1 M HEPES was added to the medium at a final concentration of 250 µM. After 6 h, the medium was replaced, TCO*A-Lys was again added at 250 µM, and the cells were incubated overnight. After incubation for 2 days at 37°C and 5% CO_2_, the ND7/23 or N1E-115-1 cells were click-labeled for the microscopy experiments. For the whole-cell patch clamp recordings, the cells were assessed for a fluorescent signal, counted and reseeded in 35 mm Petri dishes (Greiner Bio-one, cat. no. 627160) at a density of ∼180,000 cells per dish without further addition of TCO*A-Lys. The cells were incubated at 37°C and 5% CO_2_ for 2–4 h until the electrophysiological recordings were performed (always on the same day).

Primary Sprague–Dawley rat cortical neurons were transfected with Lipofectamine 2000 as described previously (Arsić et al., 2022 and see protocol at https://doi.org/10.21203/rs.3.pex-1691/v1) or with Lipofectamine 3000 according to the manufacturer's instructions. For click labeling of NF186, neurons were transfected with Lipofectamine 2000 on DIV 7 or DIV 8. For click labeling of Na_V_1.6, neurons were transfected with Lipofectamine 2000 or 3000 on DIV 8. Neurons were seeded into eight-well Lab-Tek II chambered cover glasses, and a total amount of 1 µg DNA per well was used for the transfection with Lipofectamine 2000 [0.5 µg of Na_V_1.6^WT^, Na_V_1.6^TAG^, NF186^WT^, NF186^TAG^ or pcDNA3.1/Zeo(+) and 0.5 µg NES PylRS^AF^/tRNA^Pyl^]. For the quantification of the cytosolic click signal, neurons were transfected in the same way, with an additional condition (single plasmid transfection with 0.5 µg Na_V_1.6^WT^ per well). For transfections with Lipofectamine 3000, we used a total amount of 0.5 µg DNA per well (at the DNA/Lipofectamine 3000 ratio of 1 µg/1.5 µl and a DNA/P3000 ratio of 1 µg/2 µl). The transfection mixture was prepared in 25 µl Opti-MEM Reduced Serum Medium (Thermo Fisher Scientific, cat. no. 31985062) without the addition of antibiotics. Then, 250 µl of the medium from each well was removed and saved for later at 37°C and 5% CO_2_ (conditioned medium). The entire transfection mixture was added to the well that contained the remaining 250 µl of the medium. The neurons that were transfected with Lipofectamine 2000 or 3000 were incubated with the transfection mixture for at least 6 h. Afterwards, the transfection mixture was removed, 250 µl of the conditioned medium was returned to the cells and 250 µl of fresh medium was added to each well. TCO*A-Lys in 1 M HEPES was added to a final concentration of 250 µM, and the cells were incubated at 37°C and 5% CO_2_. After incubation for 3 days, medium containing TCO*A-Lys was completely removed and replaced with one-half of the conditioned medium collected from untransfected neurons or neurons propagated without UAA, and one-half fresh culturing medium. In the controls that did not contain TCO*A-Lys, one-half of the medium was replaced with fresh culture medium. Click labeling was performed on the following day. For localization of the LOF Na_V_1.6 variants, mouse hippocampal neurons were transfected with Lipofectamine 2000 on DIV 7–8 as described above. The only difference was that the neurons were seeded in four-well Lab-Tek II chambered cover glasses. Therefore, the amounts of the DNA and the Lipofectamine 2000 transfection reagent were scaled up to correspond to the size of the four-well Lab-Tek II chambered cover glasses (twofold DNA and Lipofectamine 2000 were used).

### Click labeling of NF186 and Na_V_1.6

Click labeling of ND7/23 cells expressing NF186^WT^–HA or NF186^TAG^–HA was performed the day after transfection. The cells were labeled with 1.5 µM ATTO488-tz diluted in warm culture medium at 37°C and 5% CO_2_. After incubation for 10 min, the dye solution was removed, and the cells were washed once or twice with 0.01 M phosphate-buffered saline (PBS; 137 mM NaCl, 10 mM Na_2_HPO_4_, 1.8 mM KH_2_PO_4_, 2.7 mM KCl, pH 7.4), fixed with 4% paraformaldehyde (PFA; Sigma-Aldrich, cat. no. 158127) in 0.1 M phosphate buffer (PB) for 15 min at RT, and then washed three times with PBS. Click labeling of ND7/23 or N1E-115-1 cells expressing Na_V_1.6^WT^–HA or Na_V_1.6^TAG^–HA was performed using the procedure described above, except that 3 µM ATTO488-tz was used 40–46 h after transfection.

Click labeling of living rat cortical neurons or mouse hippocampal neurons was performed 3–4 days after transfection. The medium containing TCO*A-Lys was removed 1 day prior to labeling. For the click labeling of NF186–HA, rat neurons were labeled with 5 µM ATTO488-tz or 10 µM AF647-pyr-tz diluted in warm culture medium for 10 min at 37°C and 5% CO_2_. Then, the medium was removed, and the neurons were fixed with 4% electron-microscopy-grade PFA (Electron Microscopy Sciences, cat. no. 15710) diluted in a cytoskeleton-stabilizing buffer (PEM; 80 mM PIPES, 5 mM EGTA, 2 mM MgCl_2_, pH 6.8) for 15 min at RT and washed three times in PBS (5 min per wash). For the live-cell imaging of NF186, after click labeling, the cells were washed two or three times with culture medium, placed in a Hibernate E low fluorescence medium (Brain bits, cat. no. HELF) that contained 2% B27 Plus and 1% PS, and incubated (37°C, 5% CO_2_) until imaging. Click labeling of rat cortical neurons or mouse hippocampal neurons expressing Na_V_1.6–HA was performed using the same procedure, except that prior to labeling, the neurons were washed three times with Tyrode's solution (100 mM NaCl, 5 mM KCl, 5 mM MgCl_2_, 2 mM CaCl_2_, 15 mM D-glucose, 10 mM HEPES, pH 7.4, osmolarity 243–247 mOsm) and incubated for 3 min in 1% BSA in Tyrode's solution (Bessa-Neto et al., 2021). After incubation for 10 min with 5 µM ATTO488-tz or 10 µM AF647-pyr-tz, the neurons were washed four times with Tyrode's solution, fixed as described above and washed three times in PBS (5 min per wash). For the live-cell imaging of Na_V_1.6^TAG^, after the click labeling, the rat neurons were placed in the Hibernate E low fluorescence medium that contained 2% B27 Plus and 1% PS and incubated (37°C, 5% CO_2_) until imaging. For the dSTORM imaging of click-labeled neurons expressing NF186–HA or Na_V_1.6–HA (Figs 2H, 8), fixation was performed with 4% electron-microscopy-grade PFA in PEM for 6–7 min. For the dSTORM imaging of immunostained neurons expressing Na_V_1.6–HA (Fig. 7), neurons were fixed with 0.5% PFA in PEM for 10 min, followed by incubation with ice-cold methanol at −20°C for 10 min.

### Immunostaining

For immunostaining of neuroblastoma cells and primary neurons, all the blocking steps were performed at RT for 1 h. Incubation with primary antibodies was performed overnight at 4°C. The exceptions were the experiments involving dSTORM microscopy of the Na_V_1.6, for which the primary antibodies were first incubated ∼2 h at RT, followed by overnight incubation at 4°C. Incubation with secondary antibodies was performed at RT for 1 h. After incubation with the primary and secondary antibodies, the cells were washed three times with PBS (5 min per wash) and then kept in PBS at 4°C until imaging.

For immunostaining of ND7/23 cells expressing NF186–HA, the cells were permeabilized with 0.2% Tween 20 in PBS for 30 min. Afterwards, the cells were blocked with 5% FBS. Mouse anti-HA primary (1:1000) and goat anti-mouse-IgG AF(+)647 secondary antibodies were diluted in a blocking buffer. For immunostaining of rat neurons expressing NF186–HA, the neurons were permeabilized and blocked in a buffer containing 0.2% Triton X-100 (Sigma-Aldrich, cat. no. X100), 10% goat serum (Thermo Fisher Scientific, cat. no. 16210072) and 3% BSA in PBS. Rabbit anti-HA and mouse-anti-ankG (1:50; Santa Cruz Biotechnology) primary antibodies were diluted in the blocking buffer. Goat anti-rabbit-IgG AF555 and goat anti-mouse-IgG AF633 secondary antibodies were diluted in 3% BSA in PBS. For dSTORM microscopy imaging, rat cortical neurons were immunostained as described above, with a few differences. AF647-pyr-tz-labeled neurons expressing NF186^K680TAG^–HA were immunostained with rabbit anti-HA primary (1:2000) and goat anti-rabbit-IgG AF555 secondary antibodies. ATTO488-tz-labeled neurons were immunostained with AF647-conjugated anti-HA antibody (HA-AF647; 1:100) for 3 h at RT. The HA–AF647 antibody was diluted in 3% BSA in PBS. Neurons transfected with pcDNA3.1/Zeo(+) control plasmid were immunostained with the rabbit anti-panNF primary and goat anti-rabbit AF(+)647 secondary antibodies.

For immunostaining of Na_V_1.6–HA, neuroblastoma cells or primary neurons were permeabilized with 0.1% Triton X-100 in PBS for 10 min and blocked in a buffer containing 10% goat serum and 3% BSA. Rabbit anti-HA primary antibody (1:1000) was diluted in the blocking buffer. Goat anti-rabbit-IgG AF555 secondary antibody was diluted in 3% BSA in PBS. For the quantification of the mean fluorescence and AIS length in primary neurons expressing Na_V_1.6–HA, neurons were immunostained, in addition to the anti-HA primary antibody, with mouse anti-ankG primary (1:100; Neuromab) and goat anti-mouse-IgG AF633 secondary antibodies. For the dSTORM imaging, neurons were immunostained as described above, with a few differences. AF647-pyr-tz-labeled neurons expressing Na_V_1.6^K1546TAG^–HA were immunostained with rabbit anti-HA primary (1:250) and goat anti-rabbit-IgG AF555 secondary antibodies. ATTO488-tz-labeled neurons expressing Na_V_1.6^K1546TAG^–HA were immunostained with AF647-conjugated anti-HA antibody (1:50) for 3 h at RT. The antibody was diluted in 3% BSA in PBS. The ATTO488-tz-labeled neurons expressing Na_V_1.6^WT^ or Na_v_1.6^TAG^–HA were immunostained with rabbit anti-HA primary (1:250) and goat anti-rabbit-IgG AF(+)647 secondary antibodies. Neurons transfected with the pcDNA3.1/Zeo(+) control plasmid were immunostained with mouse anti-panNa_V_ primary and goat anti-mouse-IgG AF(+)647 secondary antibodies.

### Western blot analysis

At 1 day after transfection (as described in the section ‘Transfections’), cells were collected from six-well plates and lysed in cold RIPA buffer [12.5 mM Trizma hydrochloride (Sigma-Aldrich, cat. no. T5941), 37 mM NaCl (Sigma-Aldrich, cat. no. S7653), 3 mM sodium deoxycholate (Sigma-Aldrich, cat. no. D6750), pH 8] containing 1:50 protease inhibitor cocktail (PIC; Sigma-Aldrich, P8340), 1 mM phenylmethanesulfonyl fluoride (PMSF; Sigma–Aldrich, cat. no. P7626), and 50 mM NaF (Sigma-Aldrich, cat. no. S7920). Lysis was performed by incubation on ice for 30 min, followed by centrifugation (15 min, 18,000 ***g***). After centrifugation, supernatants were collected and the total protein concentration of the lysates was measured by the Bradford method (Bradford reagent, Sigma-Aldrich, cat. no. B6916).

For SDS-PAGE analysis, proteins were prepared in 4× Laemmli buffer (Bio-Rad, cat. no. 1610747) containing β-mercaptoethanol (βME; Sigma-Aldrich, cat. no. M3148) and denatured by incubation at 95°C for 5 min. Equal amounts of protein (10 µg per well) were separated according to molecular mass on NuPAGE 4–12% Bis-Tris protein gels (Thermo Fisher Scientific, cat. no. NP0329) alongside 5 µl of Precision Plus Protein WesternC ladder (Bio-Rad, cat. no. 610376). Electrophoresis was performed in 1× NuPAGE MOPS SDS running buffer (Thermo Fisher Scientific, cat. no. NP0001) for 40 min at 200 V. Then, proteins were transferred onto 0.2 μm nitrocellulose membrane (Bio-Rad, cat. no. 1704158) by semi-dry transfer using a Trans-Blot Turbo Transfer System (Bio-Rad, cat. no. 1704150) for 7 min at 25 V and 1.3 A. Total proteins were visualized by staining in Ponceau S solution [0.1% (w/v) Ponceau S in 5% (v/v) acetic acid]. The membrane was washed three times (10 min per wash) in ddH_2_O then blocked in 10% (w/v) skim milk (Carl Roth, cat. no. T145.1) in Tris-buffered saline (TBS; 20 mM Tris-HCl 150 mM, NaCl, pH 7.6) containing 0.05% Tween 20 (TBST; Sigma-Aldrich, cat. no. P7949) for 1 h. The membrane was cut in half and incubated with the rabbit anti-HA tag (SG77) polyclonal antibody (1:1000) or anti-β3-tubulin (1:1000) primary antibodies overnight at 4°C. Incubations with goat-anti-rabbit-IgG or goat anti-mouse-IgG HRP secondary antibodies (1:5000) were carried out for 2 h at RT. To avoid potential interaction between antibodies, the membrane was cut in half and incubated separately with the antibodies. Primary and secondary antibodies were diluted in 3% BSA in TBST. Membranes were washed three times in TBST (10 min per wash) after incubation with primary and secondary antibodies.

Immunoblots were visualized using Clarity Western ECL substrate (Bio-Rad, cat. no. 1705060). Chemiluminescence was visualized using an Azure 600 imager (Azure Biosystems). Full blot images are shown in Fig. S9.

### AAV production, purification and titration

A fluorescent reporter (NLS–mCherry–GFP^Y39TAG^) and the components necessary for genetic code expansion were separately cloned into a self-complementary AAV vector plasmid (encoding transgenes flanked by inverted terminal repeats) backbone (Fig. 6A and Fig. S8A). We cloned the following genetic code expansion components – NES PylRS^AF^, tRNA^Pyl^ (single copy), and eRF1^E55D^ (gifts from Dr Edward Lemke), codon-optimized NES PylRS^AF^ (Arsić et al., 2022), and a cassette containing four copies of tRNA^Pyl^ (a gift from Jason Chin, MRC Laboratory of Molecular Biology, Cambridge, UK). AAV vectors were produced by triple transfection of the HEK293T cells (ATCC CRL-3216) with the AAV vector plasmid, the AAV helper plasmid (encoding the AAV rep and cap genes; AAV7A2 and AAV9A2 caps were used in this study), and adenoviral helper plasmid at a 1:1:1 molar ratio using polyethylenimine (PEI MAX; Polysciences, Warrington, PA, USA, cat. no. 24765-1) as the transfection reagent. Ten 15 cm dishes were used to produce each AAV vector. In brief, 4×10^6^ HEK293T cells per dish were seeded 2 days before transfection. A DNA mixture (44 µg plasmids in 790 µl H_2_O and 790 µl of 300 mM NaCl per dish) and a solution of PEI (352 µl PEI, 438 µl H_2_O, and 790 µl of 300 mM NaCl per dish) were mixed together, vortexed thoroughly, incubated at RT for 10 min and then added to the cells dropwise. At 3 days after transfection, the cells were harvested with a cell scraper and centrifuged at 800 ***g*** for 15 min. The cell pellets were resuspended in a 5 ml Benzonase buffer (150 mM NaCl, 50 mM Tris-HCl, 2 mM MgCl_2_, pH 8.5) and lysed by subjecting them to five freeze–thaw cycles. Lysates were sonicated for 80 s and incubated with 75 U/ml Benzonase (Merck, Darmstadt, Germany, cat. no. 1.01695.0001) at 37°C for 1 h. Then, the lysates were centrifuged twice at 4000 ***g*** for 15 min to remove cell debris.

The AAV vectors in the abovementioned supernatant were purified with an iodixanol gradient [OptiPrep (iodixanol); Progen, Heidelberg, Germany, cat. no. 1114542]. Each vector was loaded into ultracentrifugation tubes (Seton Scientific, Petulama, CA, USA) through a Pasteur pipette, followed by 2 ml of 15%, 25%, 40% and 60% iodixanol solution. The tubes were sealed and centrifuged at 50,000 rpm at 4°C for 2 h in an OptimaTM L-90 K ultracentrifuge using the 70.1Ti rotor (Beckman Coulter, Brea, CA, USA). After centrifugation, 1 ml of the solution at the interface between the 40% and 60% phases was collected and stored at −80°C.

The AAV titers were measured using droplet digital PCR (ddPCR). The AAV samples were diluted 1:10^6^. Reaction solutions (20 µl) containing 900 nM primers, 25 nM probe, 10 µl of the 2× ddPCR Supermix for probes (no dUTP; Bio-Rad, Hercules, USA, cat. no. 1863024), and 5 µl of the diluted AAV sample, were prepared for generating droplets. The droplets were generated using a QX200 Droplet Generator (Bio-Rad, Hercules, USA), and then transferred to a 96-well PCR plate. The PCR was performed in a C1000 Touch Thermal Cycler (Bio-Rad, Hercules, USA), and the results were read by a QX200 Droplet Reader (Bio-Rad, Hercules, USA). The primers used for ddPCR are given in Table S27.

### Transduction with AAVs

For transduction of the primary neurons with AAVs, we adjusted the desired multiplicity of infection (MOI) by diluting AAV stocks in warm fresh culture medium supplemented with 2% B27 Plus and 1% PS. Prior to transduction, an equal amount of conditioned medium was added to the prewarmed fresh medium containing the AAV.

Primary neurons were transduced at DIV 5 with AAV9A2 or AAV7A2 encoding the NLS–mCherry–GFP^Y39TAG^ expressed from the CMV promoter without the addition of TCO*A-Lys. An MOI of 50,000 was used. For the genetic code expansion of GFP^Y39TAG^, we co-transduced rat primary cortex neurons at DIV 8 with AAV9A2 vectors (Fig. S8) encoding the CMV-NLS–mCherry–GFP^Y39TAG^ (AAV#6) and the following genetic code expansion components: (1) CMV-NES PylRS^AF^ (AAV#1) and four copies of tRNA^Pyl^ (AAV#2); (2) AAV#1, AAV#2, and mutant eukaryotic release factor eRF1^E55D^ expressed from the CMV promoter (AAV#3); (3) AAV9A2 carrying minimal (min) CMV-NES PylRS^AF^ and one copy of tRNA^Pyl^ (AAV#4); (4) AAV#4 and AAV#3; (5) AAV#1 and AAV9A2 carrying minCMV-eRF1^E55D^ and one copy of tRNA^Pyl^ (AAV#5); (6) AAV#4 and AAV#5. An MOI of 15,000 was used for each AAV. TCO*A-Lys in 1 M HEPES was added immediately upon transduction at a final concentration of 250 µM. The neurons were assessed daily for their fluorescence signal and viability. At 3 days after transduction, the neurons were fixed with 4% PFA in PEM buffer for 15 min at RT, washed three times in PBS (5 min per wash), and kept at 4°C until imaging.

To compare the efficiencies of transfection with transduction (Fig. 6), we transfected primary rat cortical neurons at DIV 8 with 0.5 µg Na_V_1.6^TAG^–HA using Lipofectamine 2000, as described in the section ‘Transfections’. After 6 h, we removed the transfection mixture and added 250 µl of the conditioned medium that was saved previously and 250 µl of fresh medium containing the following combinations of AAV92A – AAV#1 (MOI 15,000) and AAV#2 (MOI 15,000 or 25,000) or codon-optimized CMV-NES PylRS^AF^ (AAV#7; MOI 15,000) and AAV#2 (MOI 15,000 or 25,000). TCO*A-Lys in 1 M HEPES was added immediately upon transduction at a final concentration of 250 µM. The neurons were assessed daily for viability. At 3 days after transfection–transduction, we click-labeled the neurons with 5 µM ATTO488-tz, as described in the section ‘Click labeling of NF186 and Na_V_1.6’, and fixed the neurons immediately with 4% PFA in PEM buffer for 15 min at RT.

For dSTORM imaging of Nav1.6-expressing neurons (Figs 7 and 8), we transfected neurons at DIV 8 with 0.5 µg of Na_V_1.6–HA or pcDNA3.1/Zeo(+) plasmid using Lipofectamine 2000 as described in the ‘Transfections’ section. Then, the neurons were co-transduced with AAV#1 (MOI of 15,000) and AAV#2 (MOI of 15,000), as described above. Please note that these experiments were performed before we had the problem with AAV#1 potency drop. After incubation with TCO*A-Lys for 4 days, neurons were click-labeled, fixed and immunostained as described in the corresponding sections above.

### Fixed- and live-cell imaging

Confocal imaging was performed on an LSM 710 confocal scanning microscope (Carl Zeiss, Oberkochen, Germany) equipped with the following laser lines (nm): 405, 440, 458, 488, 514, 561 and 633. Images were acquired using an oil Plan-Apochromat 63× objective (NA 1.4) with the following settings: 1024×1024 pixel frame size, 16-bit image depth, 2× line averaging, 6.30 μs pixel dwell time and 0.132 μm pixel size. We used a 488 nm laser line to excite ATTO488-tz, a 561 nm laser line to excite AF555, and a 633 nm laser line to excite AF(+)647 or AF633. The pinhole was set at 1 Airy unit in all channels, and the emission light was collected sequentially. Images were acquired in two channels (488 and 561 nm, or 488 and 633 nm) or three channels (488, 561 and 633 nm), either as single planes or as *Z*-stacks with a step size of 0.42 μm.

For live-cell confocal imaging, we used a temperature module and a heating insert (PeCon, Erbach, Germany) that was warmed to 37°C. Live rat cortical neurons were imaged in Hibernate E medium supplemented with 2% B27 Plus and 1% PS.

Widefield epifluorescence and 3D dSTORM imaging were performed on an Inverted Nikon Eclipse Ti2-E microscope (Nikon Instruments) equipped with an *XY* motorized stage; a Perfect Focus System; oil-immersion objectives (Apo 60×, NA 1.4, oil and HP Apo TIRF 100×H, NA 1.49, oil); an N-STORM module; the following filter cubes: 488 (AHF; EX 482/18; DM R488; BA 525/45), 561 (AHF; EX 561/14; DM R561; BA 609/54), 647 (AHF; EX 628/40; DM660; BA 692/40), and Nikon Continuous STORM (405/488/561/647 nm Quad Band set); the following laser lines (nm): 405, 488, 561 and 647; and ORCA-Flash 4.0 sCMOS camera (Hamamatsu Photonics). The setup was controlled in NIS-Elements AR software (Nikon Instruments).

To compare the efficiencies of transfection with transduction, and the efficiency of the CMV and hNSE promoters, images of neurons were acquired in the widefield mode with an Apo 60× oil objective, 30 ms exposure time, 1024×1024 pixel frame size, 16-bit image depth, and 0.27 µm pixel size. For the widefield imaging, a fluorescent lamp (Lumencor Sola SE II) was used as a light source with the intensity set at 20%.

### Image processing

All the images shown in the main body and supplementary figures were processed in ImageJ/Fiji software (Schindelin et al., 2012) or Nikon NIS Elements. Raw confocal single planes or *Z*-stacks and widefield single-plane images were imported in Fiji. The brightness and contrast of the 16-bit images were linearly adjusted. The *Z*-stacks were converted into maximum intensity projections prior to the linear adjustment of the brightness and contrast. For presentation purposes, all images were converted to 8-bit depth, exported as .tiff files, and arranged into figures using Adobe Illustrator.

The schemes presented in the manuscript were made in BioRender.com.

### 3D dSTORM imaging, image processing and quantitative analysis of AIS periodicity

3D dSTORM imaging was performed on the N-STORM module of the Inverted Nikon Eclipse Ti2-E microscope. 3D dSTORM images of rat cortical neurons were acquired with an HP Apo TIRF 100×H objective, a 647 nm laser line (LU-NV Series Laser Unit) and a Nikon Continuous STORM filter cube (405/488/561/647 nm Quad Band set). The emitted light was imaged with an ORCA-Flash 4.0 sCMOS camera that contained a cylindrical lens introduced into the light path (Huang et al., 2008). Prior to imaging, the AIS of interest was identified using widefield microscopy. Next, an image of the same AIS was captured with a 30 ms exposure time and 647 nm laser (panNF, panNa_V_, HA or click channel) set at 1% of the power, followed by 3D dSTORM. 3D dSTORM imaging was performed in the total internal reflection fluorescence (TIRF) or highly inclined and laminated sheet microscopy (HILO) mode with continuous 647 nm laser illumination set at full power (100%). The frame size was 128×128 pixels, and the image depth 16 bit. For each 3D dSTORM image, 16,000–20,000 frames were acquired at 33.3Hz. During acquisition, 405 nm laser illumination set at 5% was used. The microscope was calibrated using TetraSpeck Microspheres (Thermo Fisher Scientific, cat. no. T7279) according to the instructions in NIS-Elements. Imaging was performed in the GLOX βME buffer, which was freshly prepared by mixing 7 µl βME, 7 μl GLOX solution (14 mg glucose oxidase; Sigma-Aldrich, cat. no. G2133), 50 μl catalase (17 mg/ml; Sigma-Aldrich, cat. no. C3155), and 200 μl Buffer A (10 mM Tris-HCl, 50 mM NaCl, pH 8) with 690 μl Buffer B (50 mM Tris-HCl, 10 mM NaCl, pH 8) containing 20% (w/v) glucose (Sigma-Aldrich, cat. no. D9559).

The 3D dSTORM images were processed in NIS-Elements AR software. Before analysis, each movie was checked and, if necessary, initial frames in which the blinking was incomplete were removed. The default molecule identification settings were used for the analysis: minimum width 200 nm, maximum width 700 nm, initial fit width 300 nm, maximum axial ratio 2.5, and maximum displacement 1. The minimum height for the peak detection was set to automatic or adjusted manually to 200–350 for each image. The localization analysis was performed with overlapping peak algorithms and the default drift correction (based on the autocorrelation) in NIS Elements.

For the quantification of AIS periodicity, the resulting lists that contained localized molecules were exported as .txt files (*Z*-rejected molecules had been filtered before exporting the molecule lists). These lists were imported into Fiji and translated into ThunderSTORM files using a previously described custom-written macro set (https://github.com/cleterrier/ChriSTORM; Leterrier et al., 2015; Ovesný et al., 2014). Next, we carried out post-processing in Fiji using the ThunderSTORM plugin (Ovesný et al., 2014). This included removal of duplicate molecules and the merging of molecules that appear in multiple frames. The final dSTORM images were reconstructed with a pixel size of 16 nm and by using the average shifted histogram visualization algorithm. The periodicity at the AIS was determined by using autocorrelation analysis (He et al., 2016; Vassilopoulos et al., 2019; Zhong et al., 2014). Briefly, it was measured in the reconstructed images using a previously described autocorrelation plugin (https://github.com/cleterrier/Process_Profiles). We measured the autocorrelation coefficient in 1–4 line regions of interest (ROIs) per AIS. Next, we either analyzed average autocorrelation curves or those of individual ROIs. The first non-zero positive peak of the autocorrelation curve was fitted in IgorPro9 (Wavemetrics, Portland, OR, USA) to determine its position and provide the spacing between peaks (periodic spacing). To determine the degree of periodicity, the autocorrelation amplitude was calculated by subtracting the height of the autocorrelation curve at the first valley from its height at the first non-zero positive peak. The measurements were statistically analyzed in SPSS Statistics 28.01.0 (IBM, Armonk, NY, USA).

For the analysis of photon counts and localization precision (Table S26), molecule lists (.txt files) were imported in Excel (Microsoft Corporation, Redmond, WA, USA) or SPSS to calculate the average values for each dSTORM image.

For presentational purposes in figures, the 3D dSTORM images were reconstructed with a Gaussian rendering size of 12 nm in NIS Elements, and the final 3D dSTORM images (with *Z* position height maps) were exported as .tiff files. The *Z*-rejected molecules were excluded from the final images. The .tiff images were imported and arranged into figures using Adobe Illustrator. The scale bars for dSTORM images were added in NIS-Elements or Fiji.

The dSTORM images of immunolabeled neurons (Fig. 7) were acquired in three experiments, whereas the dSTORM images of click-labeled neurons (Figs 2H and 8) were acquired in two experiments. Images with extensive drift and with a low number of identified molecules were excluded from the final analysis. The identity of the images or molecule lists was not known to the researchers performing the analyses.

### AIS counting and quantification

To compare the efficiencies between hNSE and CMV promoters, neurons co-expressing NF186^WT^–HA and NES PylRS^AF^/tRNA^Pyl^, with or without TCO*A-Lys treatment were imaged with widefield microscopy. For the analysis, images of all the neurons expressing NF186 in a given well of an eight-well Lab-Tek II chambered cover glasses were acquired. Images were collected from three independent experiments. Next, neurons were classified into two groups (group A: neurons with NF186–HA signal in the AIS; group B: neurons with mislocalized signal present in all the neuronal processes).

To compare the efficiencies between transduction and transfection, we acquired images of transfected and transduced neurons with widefield microscopy and used these to count the number of Na_V_1.6–HA-positive neurons. For each condition, images from two or three wells of an eight-well Lab-Tek II chambered cover glasses were acquired (in three independent experiments).

To quantitatively measure the length of the AIS in rat primary neurons expressing NF186 or Na_V_1.6, we used the previously described MATLAB ais.m script (https://www.mathworks.com/matlabcentral/fileexchange/28181-ais-quantification; Grubb and Burrone, 2010). Images were acquired on an LSM 710 confocal scanning microscope, as described in the section ‘Fixed- and live-cell imaging’. We processed the images according to the instructions of the ais.m script. In brief, the confocal images used to measure AIS length (shown in Figs 2E and 4A) in the neurons expressing NF186 and Na_V_1.6 immunostained with anti-ankG were processed in Fiji software. Raw confocal single planes or *Z*-stacks were imported, color channels were split, the brightness and contrast of the 16-bit images were linearly adjusted, and the different color channels were exported as separate 16-bit .tiff files. The *Z*-stacks were converted into maximum intensity projections prior to the linear adjustment of brightness and contrast. The processed images were imported into MATLAB and analyzed with the ais.m script (https://www.mathworks.com/matlabcentral/fileexchange/28181-ais-quantification; Grubb and Burrone, 2010). AIS length (measured in the ankG channel) was calculated automatically in MATLAB using the ais.m script. The images for the quantitative analysis of the AIS length were collected from four (NF186) or three (Na_V_1.6) independent experiments.

The fluorescence intensities at the AIS of click-labeled rat primary neurons expressing NF186 (Fig. 2F) or Na_V_1.6 (Figs 3D,E,F and 6C), or mouse primary hippocampal neurons expressing WT or LOF Na_V_1.6 variants (Fig. 5D) were measured in Fiji with a previously described macro set (https://github.com/cleterrier/Measure_ROIs). Images were acquired on an LSM 710 confocal scanning microscope as described in the section ‘Fixed- and live-cell imaging’. We processed images in Fiji using a previously described macro set (https://github.com/cleterrier/Process_Images) and measured the fluorescence intensity along the AIS in the click and HA channels. In brief, the processed images were imported in Fiji, and line tracings along the AIS were generated semi-automatically using the NeuronJ plugin (Meijering et al., 2004). The line tracings were generated independently in the click and HA channels. The line tracings were converted into Fiji ROIs, and saved as .zip files. Next, raw 16-bit images were imported in Fiji together with the corresponding previously generated ROIs. The fluorescence intensity was automatically measured in the click and HA channels, and the results were exported as .csv files. For the statistical analysis, we used a mean intensity measured along the ROI (along the AIS line tracing) corrected by subtracting the background (the ‘CorrMean’). The line width of the ROI for each AIS was individually adjusted according to the width of the AIS. The HA/click ratio was calculated by dividing the CorrMean measured in HA with that measured in the click channel for each AIS. Data were collected from three independent experiments, except for data from LOF variants (four).

The identity of the images was not known to the researchers performing the analyses. We did not image/analyze neurons overexpressing NF186 that had a clearly mislocalized HA signal present in all the processes. Owing to the lower expression level of the recombinant Na_V_1.6, we did not observe any neurons overexpressing Na_V_1.6 if plasmids were delivered via transfection. Therefore, we imaged all the transfected cells showing click labeling. However, in neurons transduced with the AAVs, we occasionally observed a few overexpressing neurons with a clearly mislocalized HA signal. Such neurons were excluded from the analysis. Furthermore, we did not analyze neurons that showed cell debris in the click channel, given that this impedes drawing line tracings along the AIS.

### Quantification of the somatic signal

The intensity of the somatic ATTO488 signal in the click-labeled neurons expressing Na_V_1.6 (Fig. S6C,D) was measured in Fiji using a previously described macro set (https://github.com/cleterrier/Measure_ROIs). Images were acquired on an LSM 710 confocal scanning microscope as described in the section ‘Fixed- and live-cell imaging’. After acquisition, we prepared confocal images for the analysis using a previously described macro set (https://github.com/cleterrier/Process_Images). In brief, this macro set reformats multichannel *Z*-stacks from .lsm to .tiff, splits channels, generates maximum intensity projections and combines these into single-channel (i.e. click, HA, and ankG) stacks. Next, these stacks were imported in Fiji, and 1–5 rectangular ROIs of the same size were drawn in the somatic region of the neurons. Depending on the size of the neuron, we drew fewer or more ROIs per neuronal soma to cover its surface. The ROIs were drawn in the ankG channel given that the immunostaining with this AIS marker allowed all neurons (transfected and untransfected) to be identified. The HA channel was used to identify transfected neurons expressing Na_V_1.6–HA, whereas the ATTO488-tz channel revealed the click-labeled neurons. The fluorescence intensity in the click channel was measured within the ROIs. A mean intensity corrected by subtracting the background (the CorrMean) was used for the statistical analysis. We calculated the average CorrMean by averaging all the measured ROIs for a given neuron. For this analysis, images were acquired in two independent experiments. The identity of the images was not known to the researchers performing the analyses.

### Electrophysiological recordings

Standard whole-cell voltage clamp recordings were performed in ND7/23, N1E-115-1 and N1E-115-1^β1β2^ cells in the presence of 500 nM TTX using an Axopatch 200B amplifier, a Digitata 1440A digitizer, and Clampex 10.2 data acquisition software (Axon Instruments, Union City, CA). The cells were held at −100 mV. Currents were filtered at 5 kHz and digitized at 20 kHz. Borosilicate glass pipettes had a final tip resistance of 1.8–2.5 MΩ when filled with the internal recording solution. The pipette solution contained 10 mM NaCl, 1 mM EGTA, 10 mM HEPES and 140 mM CsF. The pH was adjusted to 7.3 with CsOH, and the osmolarity to 290 mOsm/kg with mannitol. The bath solution contained 140 mM NaCl, 3 mM KCl, 1 mM MgCl_2_, 1 mM CaCl_2_, 10 mM HEPES, 20 mM tetraethylammonium chloride, 5 mM CsCl and 0.1 mM CdCl_2_. The pH was adjusted to 7.3 with CsOH, and the osmolarity to 320 mOsm/kg with mannitol.

The electrophysiology data were analyzed as previously described (Liu et al., 2019). In brief, the activation curve (conductance–voltage relationship) was derived from the current–voltage relationship. The latter was obtained by measuring the peak current at various step depolarizations from the holding potential of −100 mV according to the following formula:

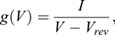
where *g* is the conductance, *I* is the recorded peak current at the test potential *V*, and *V*_rev_ represents the apparent observed Na^+^ reversal potential.

A standard Boltzmann function was fit to the activation curves according to the following formula:

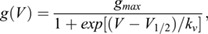
where *g*_max_ is the maximal conductance, *V*_1/2_ is the voltage of half-maximal activation, and *k*_V_ is a slope factor.

Steady-state inactivation was determined using 100 ms conditioning pulses to various potentials followed by the test pulse to −10 mV, at which the peak current reflected the percentage of non-inactivated channels. A standard Boltzmann function was fit to the inactivation curves according to the following formula:

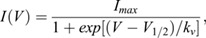
where *I* is the recorded current amplitude at the conditioning potential *V* and *I*_max_ is the maximal current amplitude.

All the data were analyzed using the Clampfit software of pClamp 10.6 (Axon Instruments), Excel or Igor Pro. For electrophysiological recordings of Na_V_1.6–HA, the data were acquired in five (K1425TAG) or six (K1546TAG) independent experiments. For the LOF experiments, the data were acquired in three (K1546TAG) or four (K1425TAG) independent experiments. All the cell recordings were performed blind per experimental group.

### Statistical analyses

Statistical analyses (Shapiro–Wilk normality test, Levene's test for equality of variances, one-way ANOVA and the non-parametric Mann–Whitney *U* or Kruskal–Wallis tests) for the quantitative analyses of all the data, except for electrophysiological recordings (see below), were performed in SPSS Statistics 28.01.0. A Shapiro–Wilk normality test indicated whether the data followed a normal distribution. Data that were normally distributed were further tested for the homogeneity of variances. The data that met both assumptions (normality and homogeneity of variances) and did not have any significant outliers were further analyzed with one-way ANOVA followed by Tukey's Honest Significant Difference (HSD) post-hoc test. The data that followed a normal distribution, but did not meet the assumption of the homogeneity of variance and/or had significant outliers were analyzed with the non-parametric Mann–Whitney *U* test (to compare two groups) or the non-parametric Kruskal–Wallis test (to compare three or more groups) with Dunn post-hoc analysis with Bonferroni correction for multiple comparisons (if required). Details are provided in Tables S1–14 and S16–S25. The corresponding graphs were created in IgorPro.

The statistical analyses of the electrophysiological recordings were performed using GraphPad Prism (GraphPad Software, San Diego, CA, USA). D'Agostino–Pearson normality tests indicated whether the data were normally distributed. To compare the groups, an unpaired two-tailed *t*-test was used for the data that followed a normal distribution, whereas the non-parametric Mann–Whitney *U* test was used for data that did not. To compare three groups of data, ANOVA on ranks with Dunnett's post-hoc test was used for data not normally distributed. The significance levels compared to the controls are indicated for all statistical tests in Table S15. The corresponding electrophysiology graphs were created in IgorPro.

The cells and neurons were allocated to experimental groups randomly. The number of experiments performed is indicated in the corresponding methods sections and figure legends. No statistical method was used to predetermine the sample size. Based on published literature and our previous similar study (Arsić et al., 2022), we aimed to collect at least 10 images or record at least 10 cells/neurons per experiment. The exclusion and anonymization criteria for image acquisition and analysis are described in the sections explaining quantitative analyses.

## Supplementary Material

10.1242/joces.260600_sup1 Supplementary information
