# Supplementary material for: Direct fluorescent labeling of NF186 and NaV1.6 in living primary neurons using bioorthogonal click chemistry
Source: J Cell Sci. 2023 Jun 28;136(12):jcs260600. doi: 10.1242/jcs.260600 (PMC10323244; doi:10.1242/jcs.260600)
Supplement: Supplementary information [file joces-136-260600-s1.pdf]

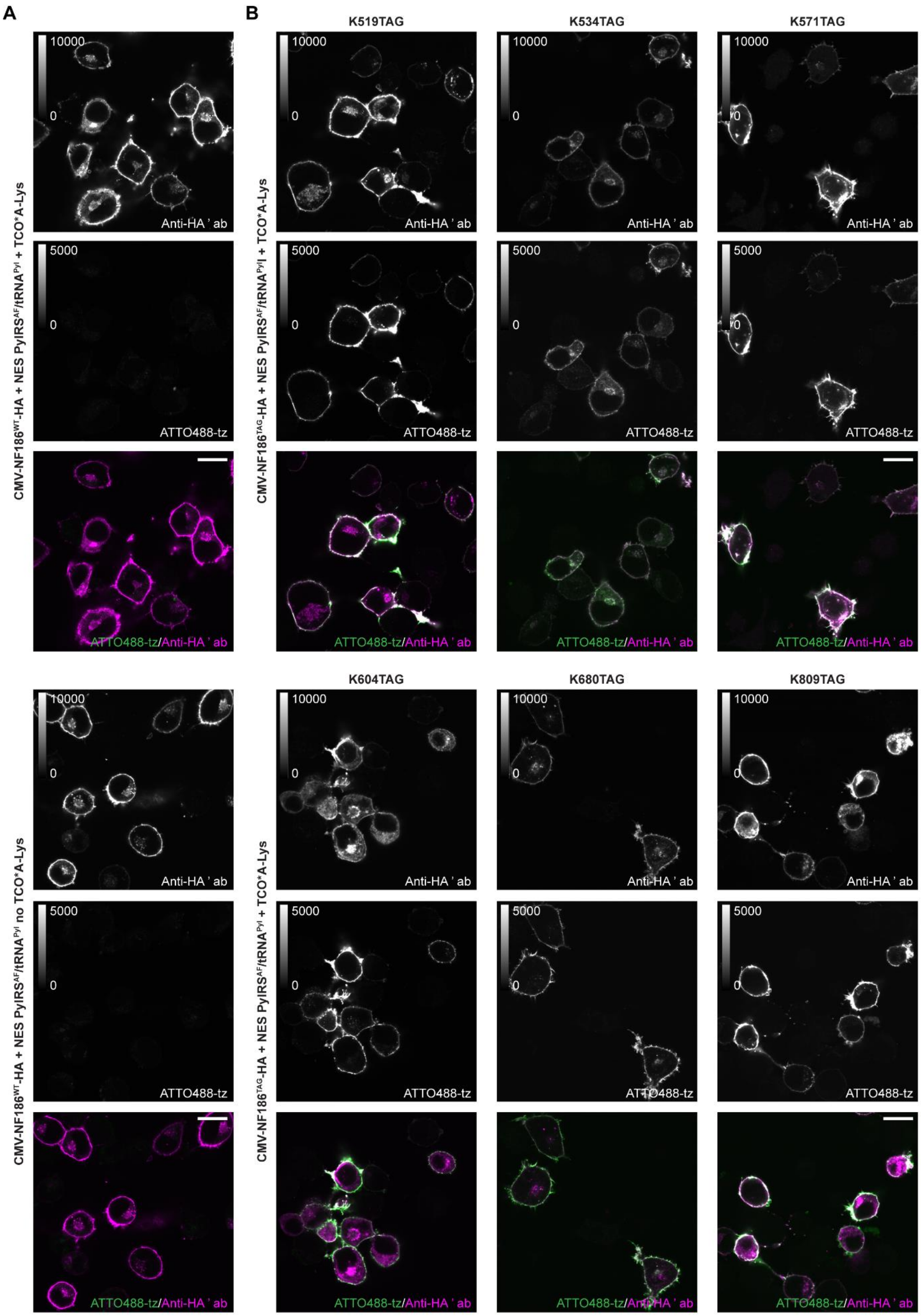

Journal of Cell Science • Supplementary information

Fig. S1. Genetic code expansion and click labeling of NF186-HA in living ND7/23 cells. (A, B) Representative images of ND7/23 cells co-expressing NES PylRS^{AF}/tRNA^{Pyl} and (A) CMV-NF186^{WT}-HA in the presence or absence of TCO*A-Lys or (B) an CMV-NF186^{TAG}-HA amber mutant (K519TAG, K534TAG, K571TAG, K604TAG, K680TAG, K809TAG) in the presence of TCO*A-Lys. One day after transfection, the cells were labeled with ATTO488-tetrazine (ATTO488-tz), fixed, and immunostained with anti-HA antibody. Single-plane images were acquired with a confocal scanning microscope. The experiment was repeated at least three times. The brightness and contrast of the panels were linearly adjusted as indicated by the lookup table (LUT) intensity scale bar. The LUT intensity scale bars show the minimum and maximum gray values. Scale bars: 20 µm. ´ab, primary antibody.

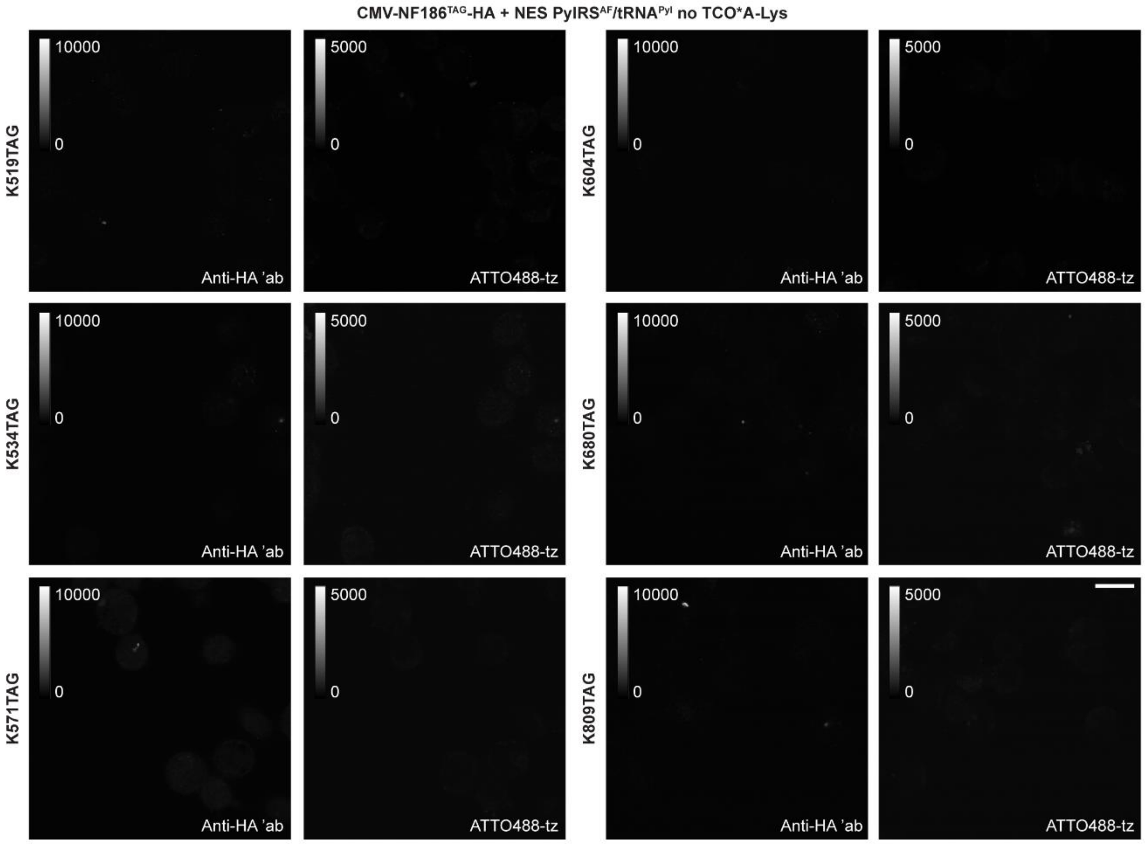

Fig. S2. Expression and click labeling of NF186^{TAG}-HA is not detected in the absence of the unnatural amino acid in ND7/23 cells. ND7/23 cells were co-transfected with the indicated constructs in the absence of TCO*A-Lys. One day after transfection, the cells were labeled with ATTO488-tz, fixed, and immunostained with anti-HA antibody. Single-plane images were acquired with a confocal scanning microscope. The experiment was repeated at least three times. The brightness and contrast of the panels were linearly adjusted as indicated by the LUT intensity scale bar. The LUT intensity scale bars show the minimum and maximum gray values. Scale bar: 20 µm. ´ab, primary antibody.

Journal of Cell Science • Supplementary information

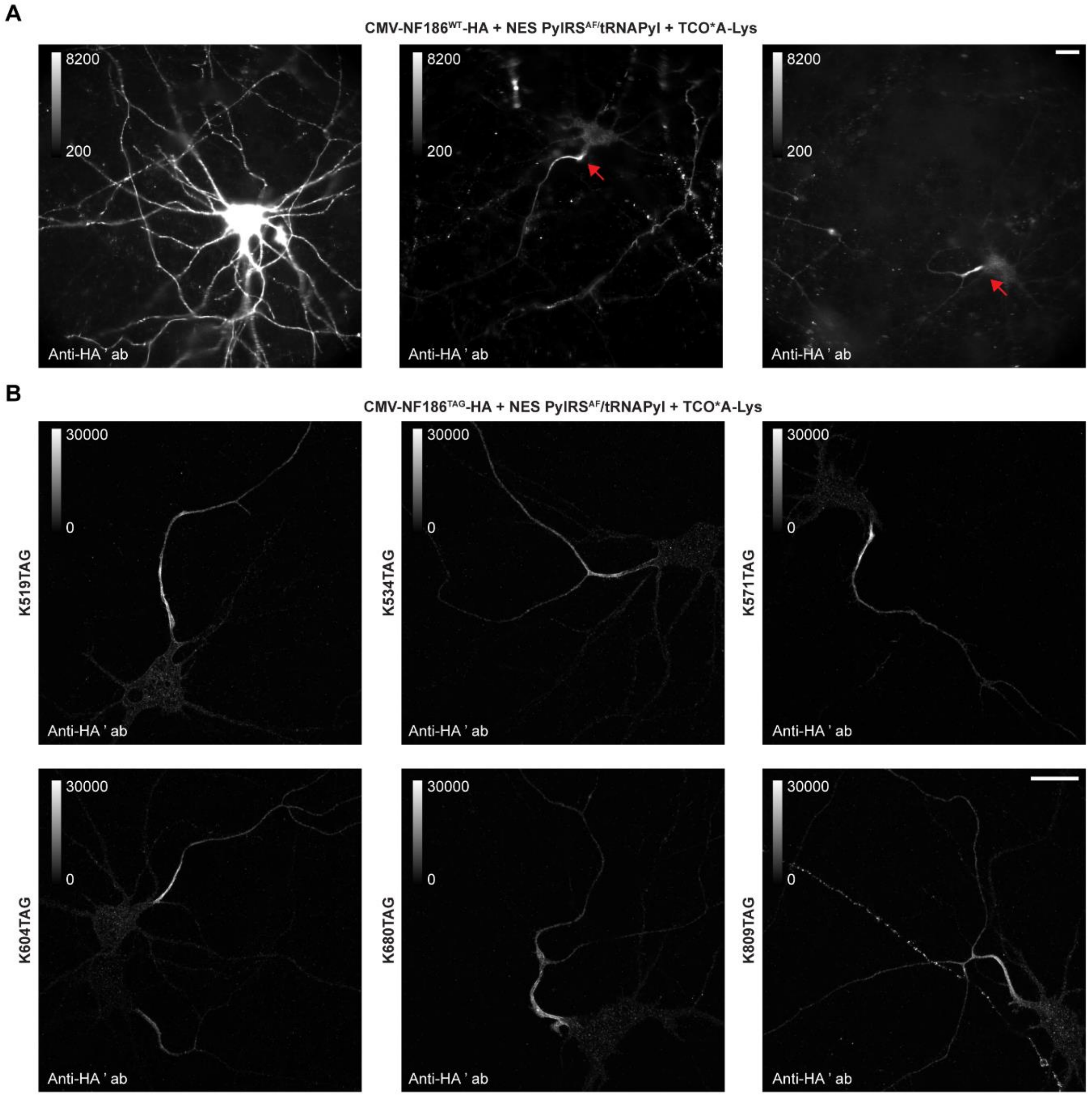

Fig. S3. NF186 expression pattern in neurons transfected with CMV-NF186^{WT}-HA or CMV-NF186^{TAG}-HA. Representative images of primary rat cortical neurons at DIV 11 co-expressing the indicated constructs in the presence of TCO*A-Lys. Four days after the transfection, the neurons were labeled with ATTO488-tz, fixed and immunostained with anti-HA antibody.

(A) Three examples of CMV-NF186^{WT}-HA expression pattern in neurons. Most of the transfected neurons showed NF186 signal in axons and dendrites (see the example image, left). The red arrows indicate axon initial segments in neurons with a moderate overexpression pattern (middle) or the expected expression pattern (right). (B) In neurons expressing CMV-NF186^{TAG}-HA constructs, the NF186 signal was frequently observed along distal axons.

Neurons that expressed NF186^{WT}-HA were imaged with widefield microscopy (A), whereas amber mutants were imaged with a confocal scanning microscope (B). The experiment was repeated at least three times.

The *Z*-stack images are shown as maximum intensity projections. The brightness and contrast of the panels were linearly adjusted as indicated by the LUT intensity scale bar. The LUT intensity scale bars show the minimum and maximum gray values. Scale bars: 20 µm. ´ab, primary antibody.

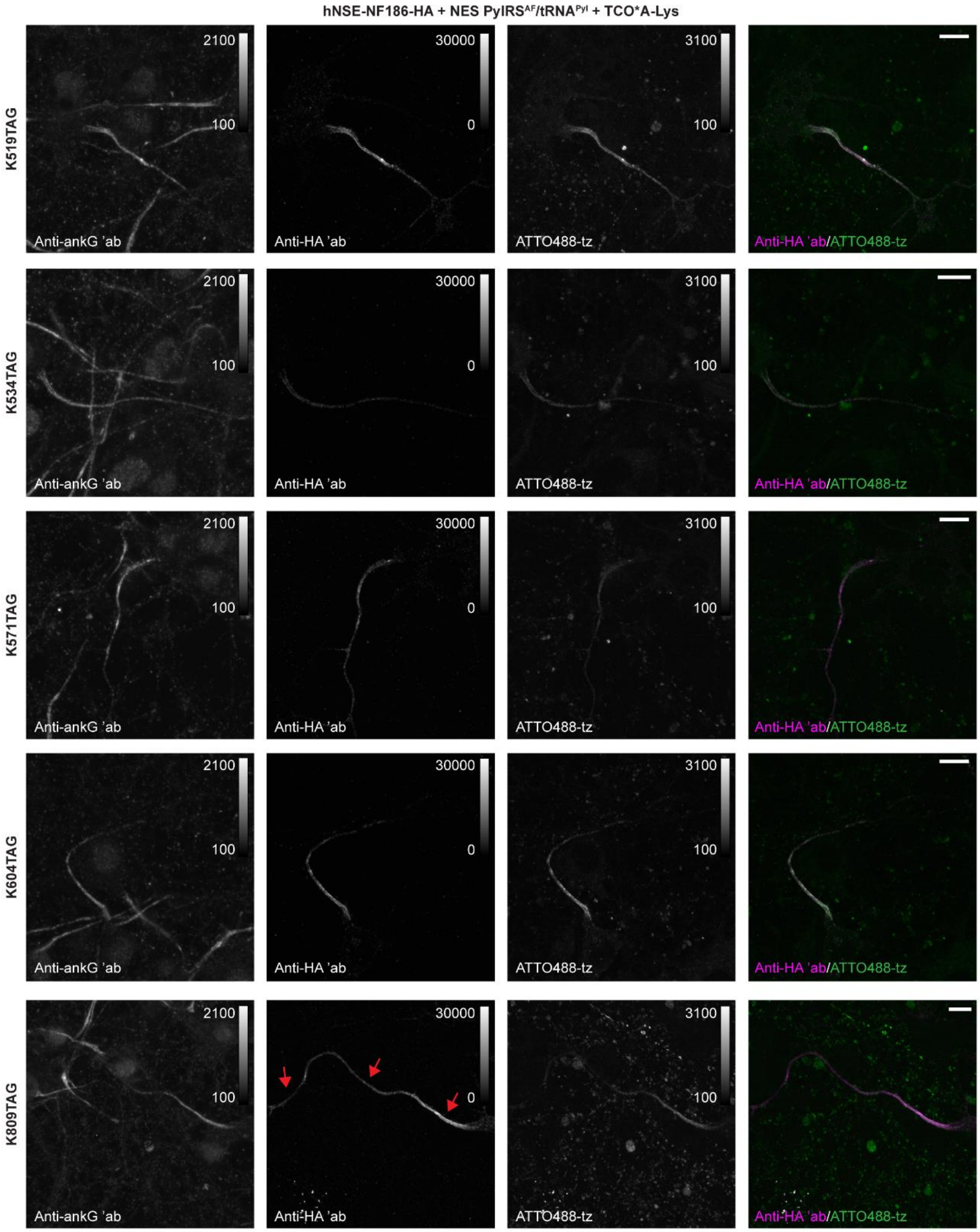

Fig. S4. Click labeling of hNSE-NF186^{TAG}-HA mutants in living primary neurons. Representative confocal images of rat cortical neurons at DIV 11 co-expressing the indicated constructs in the presence of TCO*A-Lys. Before imaging, the neurons were labeled with ATTO488-tz, fixed and immunostained with anti-HA and anti-ankyrin G (ankG) antibodies. The red arrows indicate NF186-HA signal along the axon initial segment and distal axon. The experiment was repeated at least three times. The brightness and contrast of the panels were linearly adjusted as indicated by the LUT intensity scale bar. The LUT intensity scale bars show the minimum and maximum gray values. The Z-stack images are shown as maximum intensity projections. Scale bars: 10 µm. ′ab, primary antibody.

Journal of Cell Science • Supplementary information

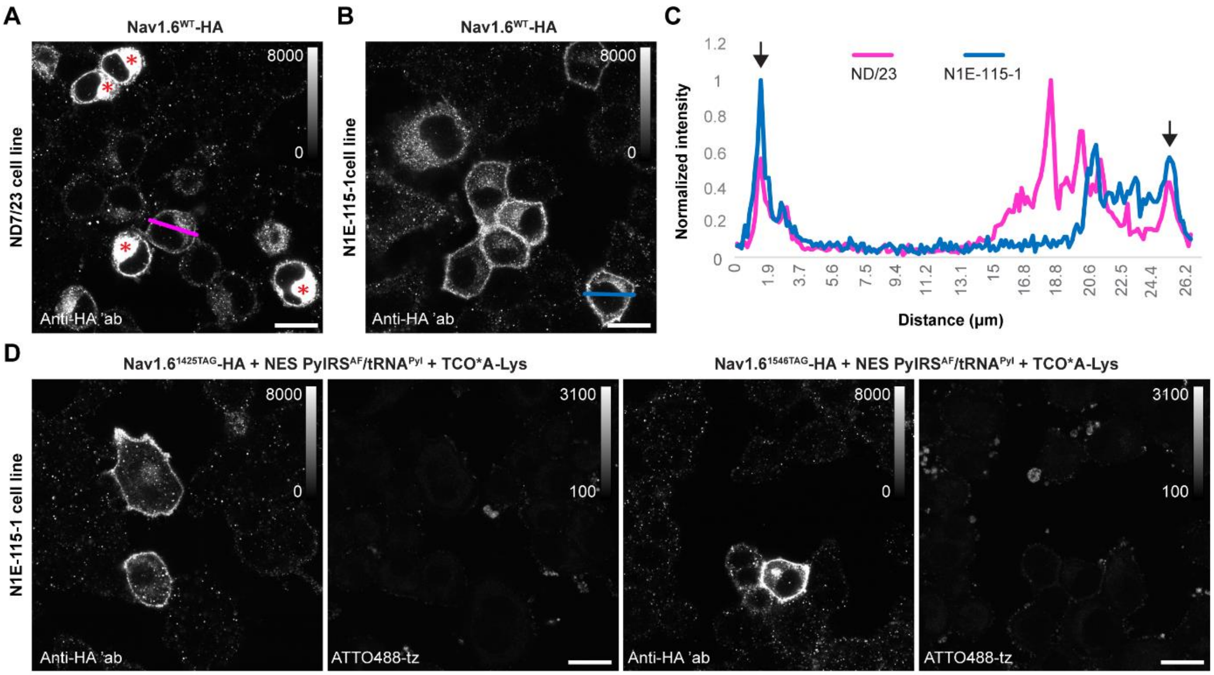

Fig. S5. Genetic code expansion and click labeling of Na$_V$1.6-HA in neuronal cells. Representative single-plane confocal images of (A) ND7/23 and (B) N1E-115-1 cells co-expressing NES PylRS^{AF}/tRNA^{Pyl}, mouse β1 and β2 subunits, and Na$_V$1.6^{WT}-HA. Two days after transfection, the cells were fixed and immunostained with anti-HA antibody. The red asterisks indicate ND7/23 cells with a strong HA signal in the cytoplasm and low signal on the cell membrane.

(C) Graph of normalized line profile fluorescence intensity measurements for the HA signal on the membrane and in the cytoplasm of the ND7/23 or N1E-115-1 cells in A and B (pink and blue lines, respectively). The arrows indicate signals at the cell membrane.

(D) N1E-115-1 cells co-expressing NES PylRS^{AF}/tRNA^{Pyl}, mouse β1 and β2 subunits, and the indicated Na$_v$1.6^{TAG}-HA plasmids in the presence of TCO*A-Lys. Two days after transfection, the cells were labeled with ATTO488-tz, fixed, and immunostained with anti-HA antibody.

The brightness and contrast of the panels were linearly adjusted as indicated by the LUT intensity scale bar. The LUT intensity scale bars show the minimum and maximum gray values. Scale bars: 20 µm. The experiment was repeated at least four times. ´ab, primary antibody.

Journal of Cell Science • Supplementary information

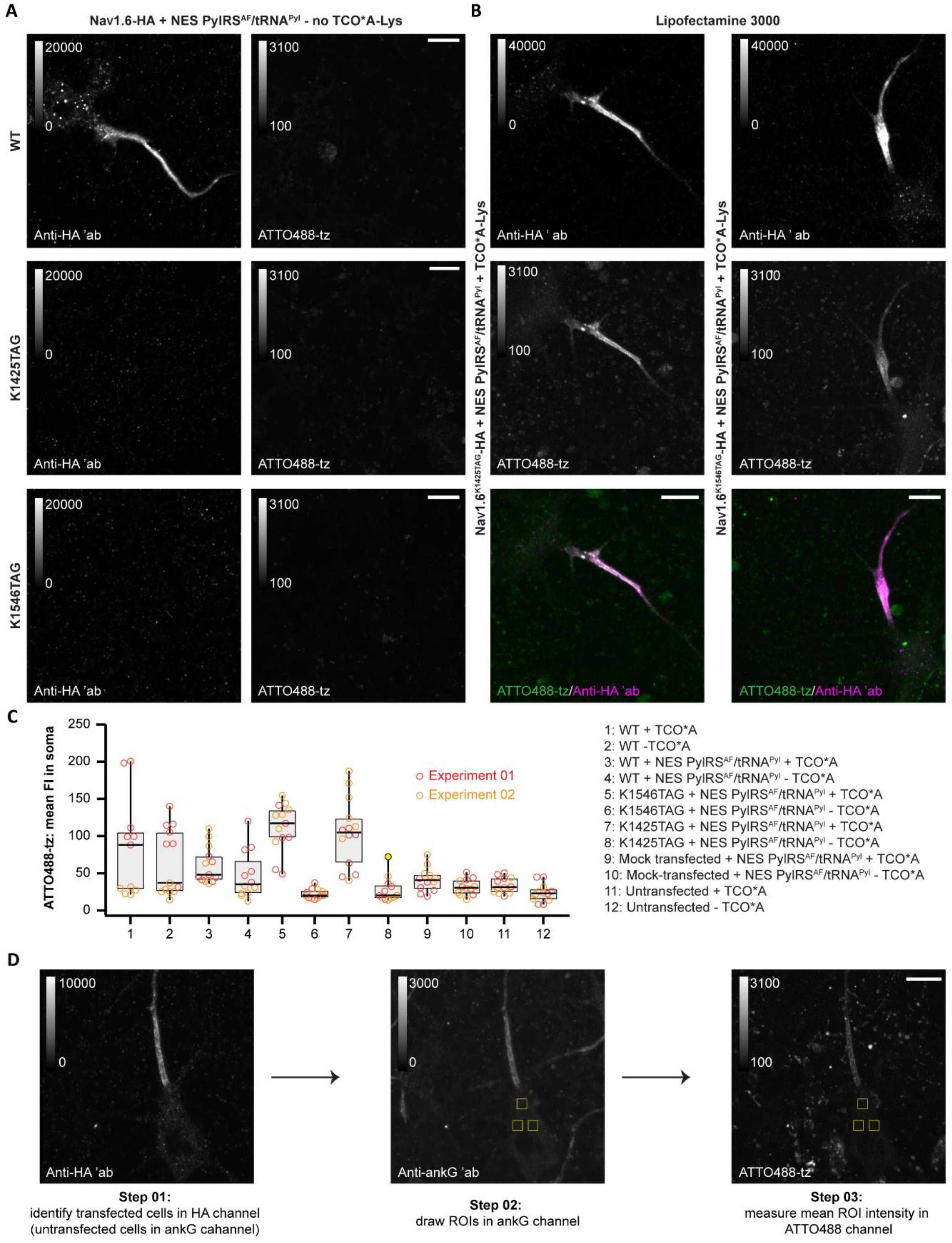

Fig. S6. Optimization of the expression and click labeling of Na$_V$1.6^{TAG}-HA in primary neurons. Primary rat cortical neurons were transfected at DIV 8 with Lipofectamine 2000 (A, D) or Lipofectamine 3000 (B) transfection reagent in the presence or absence of TCO*A-Lys. Four days later, neurons were labeled with

Journal of Cell Science • Supplementary information

ATTO488-tz, fixed, and immunostained with anti-HA antibody (A, B). In addition to the anti-HA, neurons were immunostained with anti-ankG antibody (D).

(A) Representative confocal images of rat neurons at DIV 12 co-expressing indicated constructs in the absence of TCO*A-Lys. The experiment was repeated at least three times.

(B) Representative confocal images of Lipofectamine 3000-transfected rat neurons at DIV 12 co-expressing indicated constructs in the presence of TCO*A-Lys. The experiment was repeated at least three times.

(C) A plot showing the distribution of the mean ATTO488-tz fluorescence intensities (FI) in neuronal soma. The mean FI was measured in confocal images of neurons expressing the indicated plasmids in the presence or absence of TCO*A-Lys, including untransfected controls. The box plot indicates the median (black lines inside the box), the 25th and 75th percentiles (box boundaries), single data points (dots), and outliers (yellow dots). Whisker lengths are defined by the minimum and maximum data points. The single data points represent individual neurons. Data were collected from two independent experiments (represented by different colors). Significant differences were detected between the following groups: condition 5 and conditions 6, 8, 10, 11, 12; condition 6 and conditions 1 and 3; condition 7 and conditions 6, 8, 10, 11, 12; conditions 12 and 3 (p<0.05; Kruskal–Wallis test followed by Dunn *post-hoc* analysis with Bonferroni correction for multiple comparisons; n_1=11, n_2=13, n_3=13, n_4=12, n_5=13, n_6=12, n_7=14, n_8=12, n_9=12, n_{10}=12, n_{11}=12, n_{12}=12 neurons). Details of the statistical analysis and all of the significant differences between groups are given in Table S25.

(D) Representative confocal image used for the quantitative analysis in C, including a brief description of the analysis steps. The yellow squares indicate regions of interest (ROIs) in which the mean ATTO488-tz FI was measured.

The *Z*-stack confocal images (A, B, and D) are shown as maximum intensity projections. The brightness and contrast of the panels were linearly adjusted as indicated by the LUT intensity scale bar. The LUT intensity scale bars show the minimum and maximum gray values. Scale bars: 10 µm. ´ab, primary antibody.

Journal of Cell Science • Supplementary information

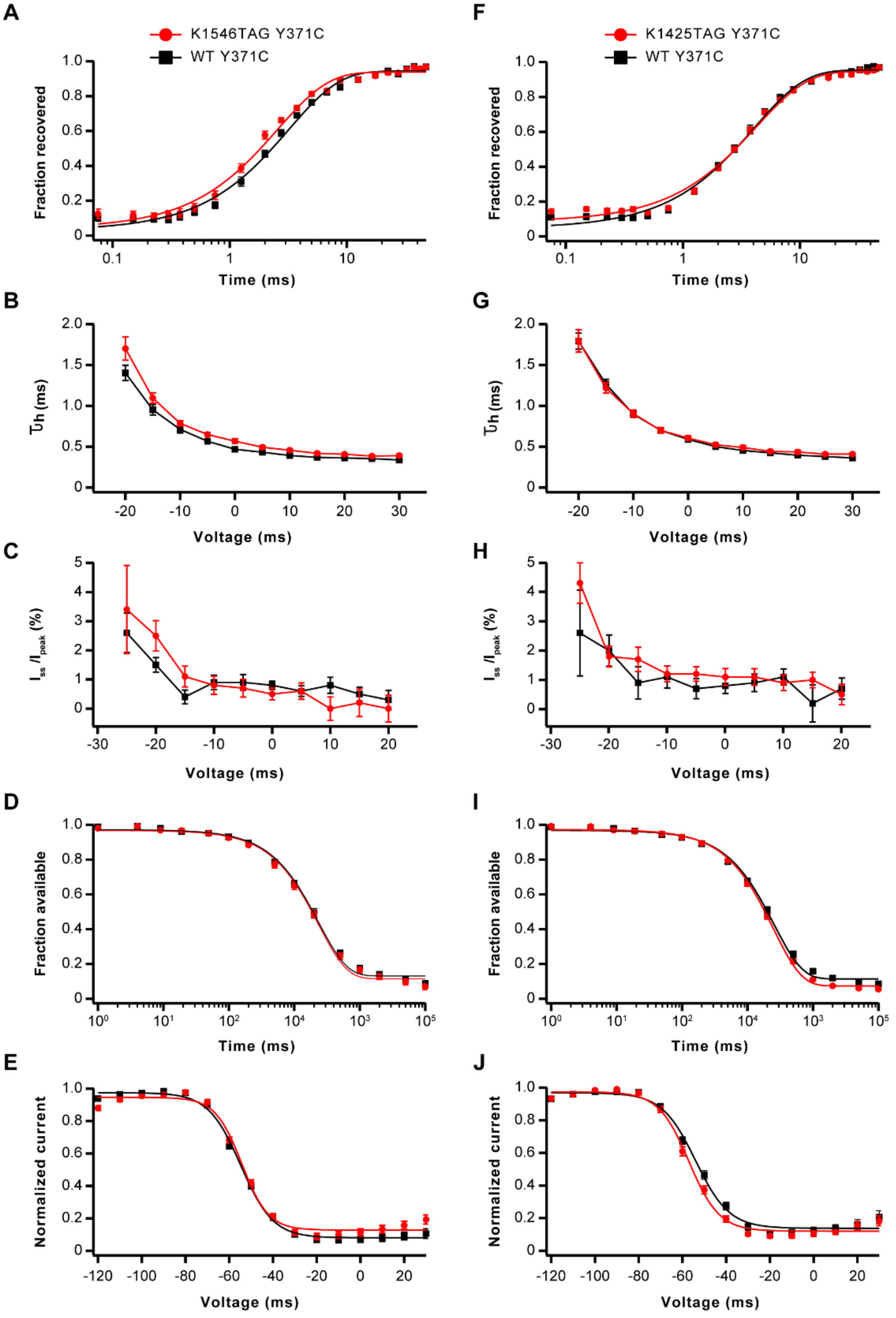

Fig. S7. Additional biophysical properties of Na$_V$1.6^{WT}-HA and Na$_V$1.6^{TAG}-HA in N1E-115-1 cells. (A–E) N1E-115-1 cells were co-transfected with NES PylRS^{AF}/tRNA^{Pyl}, multigene plasmid encoding mouse β1 and β2 subunits and GFP, and Na$_V$1.6$^{WT, Y371C}$-HA or Na$_V$1.6$^{K1546TAG, Y371C}$-HA. (F–J) N1E-115-1^{β1β2}-stable cells were co-transfected with NES PylRS^{AF}/tRNA^{Pyl}, Na$_V$1.6$^{WT, Y371C}$-P2A-eGFP, or Na$_V$1.6$^{K1425TAG, Y371C}$-P2A-eGFP. Na$^+$ currents were recorded two days after transfection in the presence of 500 nM tetrodotoxin (TTX), which

Journal of Cell Science • Supplementary information

blocks endogenous Na^+ currents. Data were collected from five (WT vs. K1425TAG) or six (WT vs. K1546TAG) independent experiments. Details of the statistical analysis, numbers of recorded cells, and all of the significant differences between groups are given in Table S15. Shown are means±s.e.m.

(A, F) Time course of recovery from fast inactivation at −100 mV. Compared to the WT channels, K1546TAG significantly (p<0.05) accelerated the recovery from fast inactivation.

(B, G) Voltage dependence of the major time constant of fast inactivation τ_h. Compared to the WT channels, K1546TAG significantly (p<0.05) delayed the transition from activation to fast inactivation.

(C, H) Voltage dependence of the persistent current [$I_{ss(steady\text{-}state)}/I_{peak}$].

(D, I) Entry into slow inactivation. The lines represent fits of a first-order exponential function to the data points.

(E, J) Steady-state slow inactivation. The lines represent Boltzmann functions fit to the data points.

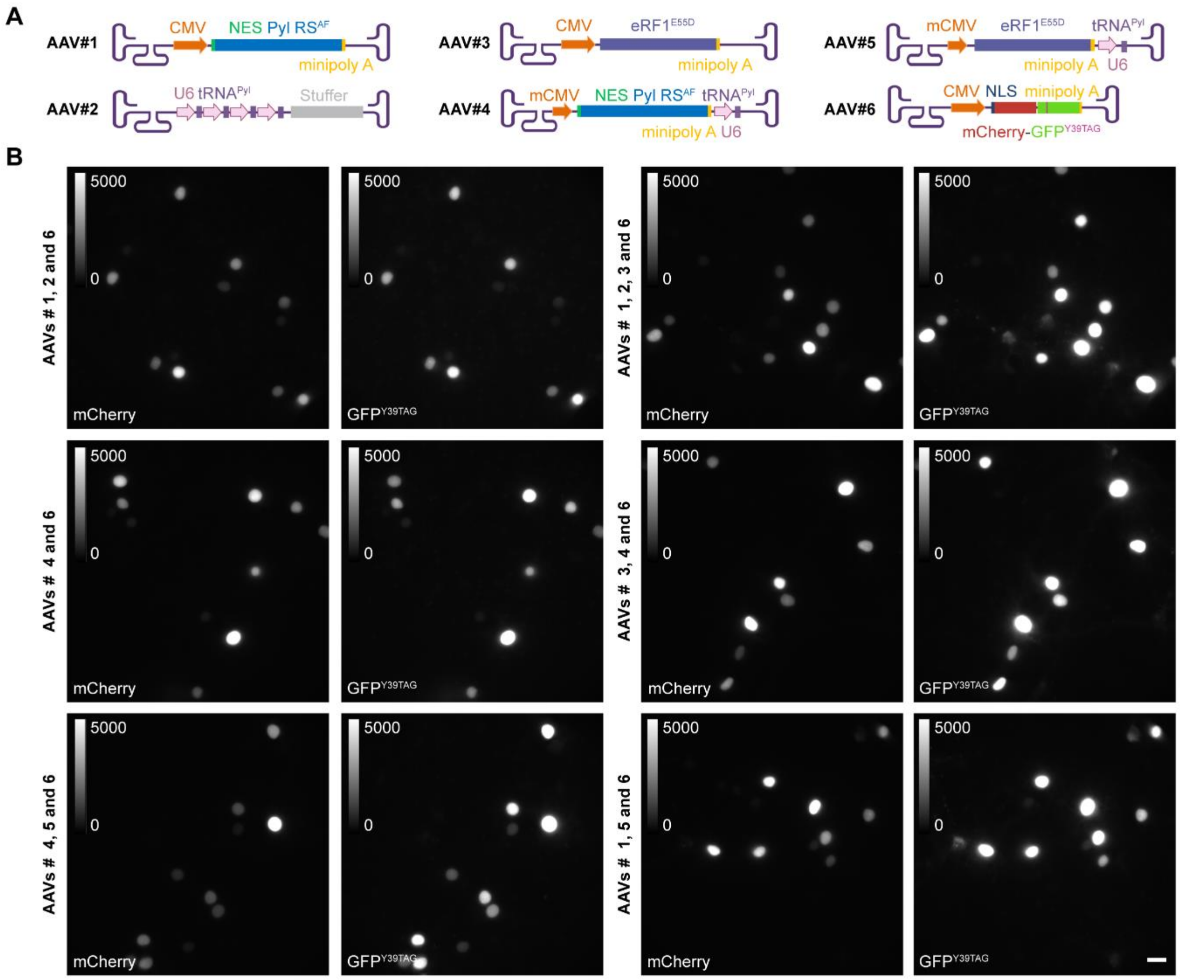

Fig. S8. AAV-based vectors for the genetic code expansion and click labeling in primary neurons.

(A) Schematic representation of AAV9A2 viral vectors used for the transduction-mediated genetic code expansion in primary rat neurons. In addition to AAV9A2 carrying a nuclear localization signal (NLS)-mCherry-GFP^{Y39TAG} fluorescent reporter cassette (AAV#6), we developed AAV9A2 vectors carrying various combinations of genes for genetic code expansion: AAV#1: NES PylRS^{AF} expressed from a CMV promoter; AAV#2: four copies of tRNA^{Pyl} expressed from a U6 promoter; AAV#3: eukaryotic release factor eRF1^{E55D} expressed from a CMV promoter; AAV#4: NES PylRS^{AF} expressed from a minimal (min) CMV promoter and one copy of tRNA^{Pyl} expressed from a U6 promoter; AAV#5: minCMV eRF1^{E55D} and one copy of U6 tRNA^{Pyl}. The scheme was created with BioRender.com.

(B) Representative widefield images of neurons at DIV 11 co-expressing the AAVs indicated. In addition to NLS-mCherry-GFP^{Y39TAG} (AAV#6), neurons were transduced with different combinations of orthogonal translational machinery components (AAV#1–5) in the presence of TCOA*-Lys. The data were collected from two independent experiments.

The brightness and contrast of the images were linearly adjusted as indicated by the LUT intensity scale bar. The LUT intensity scale bars show the minimum and maximum gray values. Scale bar: 20µm.

Journal of Cell Science • Supplementary information

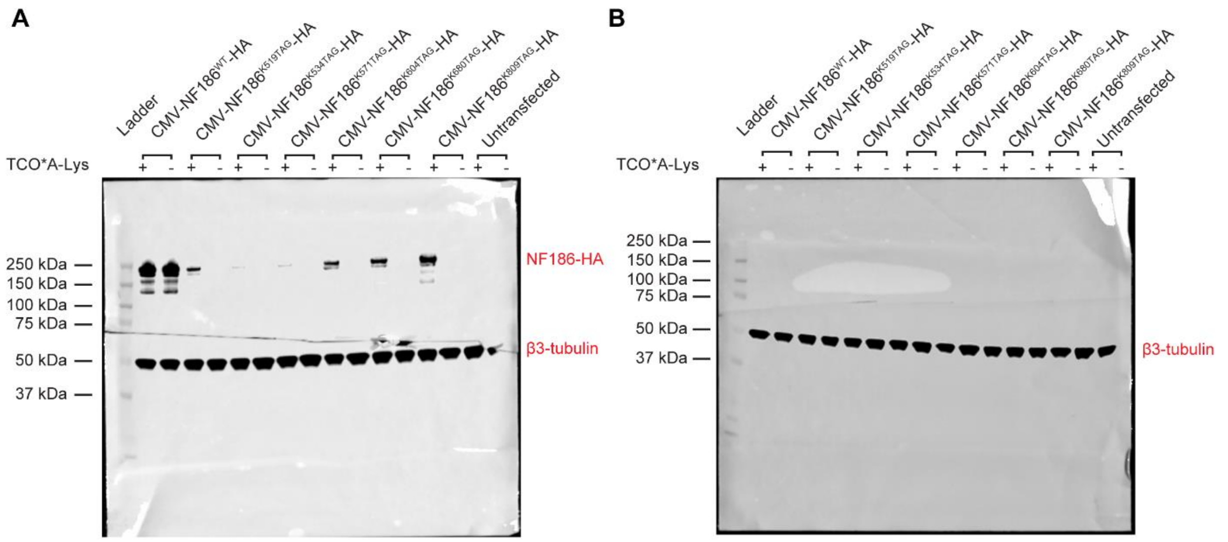

Fig. S9. Blot transparency (cropped blot is shown in Fig. 2B)**. Western blot expression analysis of the CMV-NF186-HA expression in ND7/23 cells.** ND7/23 cells were co-transfected with codon-optimized NES PylRS^{AF}/U6-tRNA^{Pyl} and CMV-NF186^{WT}-HA or with one of the CMV-NF186^{TAG}-HA amber mutants (K519TAG, K534TAG, K571TAG, K604TAG, K680TAG, K809TAG), in the presence or absence of TCO*A-Lys. One day after transfection, cell lysates were collected, and the NF186-HA expression was analyzed by western blotting. Recombinant CMV-NF186-HA was detected with an anti-HA antibody (A). No primary (anti-HA) antibody control is shown in B. The endogenous β3-tubulin, detected with an anti-β3-tubulin antibody, was used as a loading control (A, B). Experiment was repeated at least two times.

Journal of Cell Science • Supplementary information

Table S1. Pairwise comparisons of the AIS length (measured in the ankG channel; shown in Fig. 2E) between NF186-transfected (HA+) and surrounding untransfected (HA−) neurons using the independent-samples Kruskal–Wallis test.

Groups: 1. WT HA+; 2. WT HA−; 3. K519TAG HA+; 4. K519TAG HA−; 5. K604TAG HA+; 6. K604TAG HA−; 7. K680TAG HA+; and 8. K680TAG HA−. Pairwise comparisons of transfected and untransfected neurons for each of the constructs (1–2, 3–4, 5–6, and 7–8) are highlighted in light green.

Independent-Samples Kruskal-Wallis Test

Total N	155
Test Statistic	25.895[a]
Degree Of Freedom	7
Asymptotic Sig.(2-sided test)	<0.001

a. The test statistic is adjusted for ties.

Pairwise Comparisons

Comparison	Test statistic	Standard error	Standard test statistic	Significance	Adjusted Significance [a]
4.00-2.00	6.433	11.390	0.565	0.572	1.000
4.00-8.00	-9.382	12.969	-0.723	0.469	1.000
4.00-6.00	-23.138	17.448	-1.326	0.185	1.000
4.00-1.00	40.443	13.875	2.915	0.004	0.100
4.00-7.00	-41.832	13.875	-3.015	0.003	0.072
4.00-3.00	42.133	16.240	2.594	0.009	0.265
4.00-5.00	-52.360	16.795	-3.118	0.002	0.051
2.00-8.00	-2.949	11.693	-0.252	0.801	1.000
2.00-6.00	-16.705	16.523	-1.011	0.312	1.000
2.00-1.00	34.010	12.691	2.680	0.007	0.206
2.00-7.00	-35.399	12.691	-2.789	0.005	0.148
2.00-3.00	-35.700	15.241	-2.342	0.019	00.537
2.00-5.00	-45.927	15.831	-2.901	0.004	0.104
8.00-6.00	13.756	17.648	0.779	0.436	1.000
8.00-1.00	31.062	14.125	2.199	0.028	0.781
8.00-7.00	32.450	14.125	2.297	0.022	0.605
8.00-3.00	32.751	16.455	1.990	0.047	1.000
8.00-5.00	42.978	17.002	2.528	0.011	0.321
6.00-1.00	17.306	18.325	0.944	0.345	1.000
6.00-7.00	-18.694	18.325	-1.020	0.308	1.000

6.00-3.00	18.995	20.175	0.942	0.346	1.000
6.00-5.00	29.222	20.624	1.417	0.157	1.000
1.00-7.00	-1.389	14.962	-0.093	0.926	1.000
1.00-3.00	-1.689	17.178	-0.098	0.922	1.000
1.00-5.00	-11.917	17.703	-0.673	0.501	1.000
7.00-3.00	0.301	17.178	0.017	0.986	1.000
7.00-5.00	10.528	17.703	0.595	0.552	1.000
3.00-5.00	-10.227	19.612	-0.521	0.602	1.000

Notes. Each row tests the null hypothesis that the Sample 1 and Sample 2 distributions are the same. Asymptotic significances (2-sided tests) are displayed. The significance level is 0.050. a. Significance values have been adjusted by the Bonferroni correction for multiple tests.

Table S2. Comparison of the AIS mean fluorescence intensity (measured in the ATTO488 channel; shown in Fig. 2F) in neurons expressing NF186^{TAG}-HA using the independent-samples Kruskal–Wallis test.

Groups: 1. K519TAG; 2. K604TAG; 3. K680TAG.

Independent-Samples Kruskal-Wallis Test

Total N	45
Test Statistic	0.188[a,b]
Degree Of Freedom	2
Asymptotic Significance (2-sided test)	0.910

a. The test statistic is adjusted for ties.

b. Multiple comparisons are not performed because the overall test does not show significant differences across samples.

Journal of Cell Science • Supplementary information

Table S3. Comparison of the AIS mean fluorescence intensity (measured in the HA channel; shown in Fig. 2F) in neurons expressing NF186^{WT}-HA or NF186^{TAG}-HA using the independent-samples Kruskal–Wallis test.

Groups: 1. WT; 2. K519TAG; 3. K604TAG; 4. K680TAG.

Independent-Samples Kruskal-Wallis Test

Total N	55
Test Statistic	3.244[a,b]
Degree Of Freedom	3
Asymptotic Significance (2-sided test)	0.355

a. The test statistic is adjusted for ties.

b. Multiple comparisons are not performed because the overall test does not show significant differences across samples.

Table S4. Comparison of the anti-HA/ATTO488-tz ratio in neurons expressing NF186^{TAG}-HA (shown in Fig. 2F) using the independent-samples Kruskal–Wallis test.

Groups: 1. K519TAG; 2. K604TAG; 3. K680TAG.

Independent-Samples Kruskal-Wallis

Total N	45
Test Statistic	0.438[a,b]
Degree Of Freedom	2
Asymptotic Significance (2-sided	0.803

a. The test statistic is adjusted for

b. Multiple comparisons are not performed because the overall test does not show significant differences across samples.

Table S5. Comparison of the periodic spacing of NF186 (shown in Fig. 2K) measured in click-labeled neurons using the one-way ANOVA.

Groups: 1. panNF; 2. HA-AF647; 3. AF647-Pyr-tz.

ANOVA

	Sum of Squares	Degree of freedom	Mean Square	F	Significance
Between Groups	0.001	2	0.001	0.666	0.518[a]
Within Groups	0.057	59	0.001		
Total	0.058	61			

[a]*Although we did not observe an overall significant difference between groups which as such does not require post-hoc analysis, post-hoc Tukey's Honest Significant Difference (HSD) test was performed to provide further evidence that none of the pairwise comparisons showed a significant difference among groups.*

Tukey HSD

(I) Category	(J) Category	Mean Difference (I-J)	Standard Error	Significance	95% Confidence Interval	
					Lower Bound	Upper Bound
1	2	0.003935919	0.009684519	0.913	-0.01934805	0.02721989
	3	-0.006965367	0.009684519	0.753	-0.03024934	0.01631861
2	1	-0.003935919	0.009684519	0.913	-0.02721989	0.01934805
	3	-0.010901286	0.009565686	0.494	-0.03389955	0.01209698
3	1	0.006965367	0.009684519	0.753	-0.01631861	0.03024934
	2	0.010901286	0.009565686	0.494	-0.01209698	0.03389955

Table S6. Comparisons of the degree of periodicity (shown in Fig. 2L) of NF186 measured in click-labeled neurons using the independent-samples Kruskal–Wallis test.

Groups: 1. panNF; 2. HA-AF647; 3. AF647-Pyr-tz.

Independent-Samples Kruskal-Wallis Test

Total N	62
Test Statistic	0.036[a,b]
Degree Of Freedom	2
Asymptotic Significance (2-sided test)	0.982

a. The test statistic is adjusted for ties.

b. Multiple comparisons are not performed because the overall test does not show significant differences across samples.

Table S7. Comparisons of the AIS mean fluorescence intensity (measured in the HA channel; shown in Fig. 3D) in neurons expressing $Na_V1.6^{WT}$-HA or $Na_V1.6^{TAG}$-HA using the independent-samples Kruskal–Wallis test.

Groups: 1. WT; 2. K1425TAG; 3. K1546TAG.

Independent-Samples Kruskal-Wallis

Total N	51
Test Statistic	2.703[a,b]
Degree Of	2
Asymptotic Significance (2-sided	0.259

a. The est statistic is adjusted for

b. Multiple comparisons are not performed because the overall does not show significant differences across samples.

Journal of Cell Science • Supplementary information

Table S8. Comparison of the AIS mean fluorescence intensity (measured in the ATTO488 channel; shown in Fig. 3D) between neurons expressing $Na_V1.6^{K1425TAG}$-HA and $Na_V1.6^{K1546TAG}$-HA using the independent-samples Mann–Whitney *U* test.

Independent-Samples Mann-Whitney *U* Test

Total N	29
Mann-Whitney U	181.000
Wilcoxon W	334.000
Test Statistic	181.000
Standard Error	22.583
Standardized Test Statistic	3.498
Asymptotic Significance (2-sided test)	<0.001
Exact Significance (2-sided test)	0.000

Table S9. Comparison of the anti-HA/ATTO488-tz ratio of the AIS mean fluorescence intensity (shown in Fig. 3D) between neurons expressing $Na_V1.6^{K1425TAG}$-HA and $Na_V1.6^{K1546TAG}$-HA using the independent-samples Mann–Whitney *U* test.

Independent-Samples Mann-Whitney *U* Test

Total N	29
Mann-Whitney U	29.000
Wilcoxon W	182.000
Test Statistic	29.000
Standard Error	22.583
Standardized Test Statistic	-3.232
Asymptotic Significance (2-sided test)	0.001
Exact Significance (2-sided test)	0.001

Journal of Cell Science • Supplementary information

Table S10. Comparison of the AIS mean fluorescence intensity of $Na_V1.6^{K1425TAG}$-HA (measured in the ATTO488 channel, shown in Fig. 3E) between neurons transfected with Lipofectamine 2000 and Lipofectamine 3000 transfection reagents using the independent-samples Mann–Whitney *U* test.

Independent-Samples Mann-Whitney *U* Test

Total N	24
Mann-Whitney U	90.000
Wilcoxon W	145.000
Test Statistic	90.000
Standard Error	17.078
Standardized Test Statistic	1.171
Asymptotic Significance (2-sided test)	0.242
Exact Significance (2-sided test)	0.259

Table S11. Comparison of the AIS mean fluorescence intensity of $Na_V1.6^{K1425TAG}$-HA (measured in the HA channel; shown in Fig. 3E) between neurons transfected with Lipofectamine 2000 and Lipofectamine 3000 transfection reagents using the independent-samples Mann–Whitney *U* test.

Independent-Samples Mann-Whitney *U* Test

Total N	24
Mann-Whitney U	90.000
Wilcoxon W	145.000
Test Statistic	90.000
Standard Error	17.078
Standardized Test Statistic	1.171
Asymptotic Significance (2-sided test)	0.242
Exact Significance (2-sided test)	0.259

Journal of Cell Science • Supplementary information

Table S12. Comparison of the AIS mean fluorescence intensity of $Na_V1.6^{K1546TAG}$-HA (measured in the ATTO488-tz channel; shown in Fig. 3F) between neurons transfected with Lipofectamine 2000 and Lipofectamine 3000 transfection reagents using the independent-samples Mann–Whitney *U* test.

Independent-Samples Mann-Whitney *U* Test

Total N	19
Mann-Whitney U	48.000
Wilcoxon W	114.000
Test Statistic	48.000
Standard Error	12.111
Standardized Test Statistic	0.330
Asymptotic Significance (2-sided test)	0.741
Exact Significance (2-sided test)	0.778

Table S13. Comparison of the AIS mean fluorescence intensity of $Na_V1.6^{K1546TAG}$-HA (measured in the HA channel; shown in Fig. 3F) between neurons transfected with Lipofectamine 2000 and Lipofectamine 3000 transfection reagents using the independent-samples Mann–Whitney *U* test.

Independent-Samples Mann-Whitney *U* Test

Total N	19
Mann-Whitney U	63.000
Wilcoxon W	129.000
Test Statistic	63.000
Standard Error	12.111
Standardized Test Statistic	1.569
Asymptotic Significance (2-sided test)	0.117
Exact Significance (2-sided test)	0.129

Table S14. Comparison of the AIS length (measured in the ankG channel; shown in Fig. 4A) between recombinant $Na_V1.6^{WT}$-HA or $Na_V1.6^{TAG}$-HA transfected (HA+) and surrounding untransfected (HA−) neurons using the independent-samples Kruskal–Wallis test.

Groups: 1. WT HA+; 2. WT HA−; 3. K1425TAG HA+; 4. K1425TAG HA−; 5. K1546TAG HA+; 6. K1546TAG HA.

Independent-Samples Kruskal-Wallis Test

Total N	117
Test Statistic	9.538[a,b]
Degree Of Freedom	5
Asymptotic Significance (2-sided test)	0.089

a. The test statistic is adjusted for ties.

b. Multiple comparisons are not performed because the overall test does not show significant differences across samples.

Table S15. Biophysical properties of $Na_V1.6^{WT,\ Y371C}$-HA and $Na_V1.6^{K1546TAG,\ Y371C}$-HA recorded in neuronal N1E-115-1 cells, or $Na_V1.6^{WT,\ Y371C}$-P2A-eGFP and $Na_V1.6^{K1425TAG,\ Y371C}$-P2A-eGFP recorded in neuronal N1E-115-1$^{\beta1\beta2}$-stable cells (shown in Fig. 4C–F and Fig. S7).

	**Steady-state activation**			**Steady-state inactivation**			**τ_h at 0 mV (ms)**	***n***	**τ_{rec} at −100 mV (ms)**	***n***	**Current density (pA/pF)**	***n***
	**$V_{1/2}$ (mV)**	***k***	***n***	**$V_{1/2}$ (mV)**	***k***	***n***						
**$Na_V1.6^{WT}$**	−9.9±1.1	−7.3±0.3	18	−58.8±0.5	4.9±0.1	18	0.59±0.02	18	3.6±0.2	17	−124.6±15.5	18
**$Na_V1.6^{K1425TAG}$**	−11.4±0.6	−7.4±0.2	20	−60.2±0.5	4.8±0.1	20	0.60±0.03	20	3.6±0.2	20	−89.0±5.5* (*p*=0.0426)	20
**$Na_V1.6^{WT}$**	−11.8±1.0	−6.8±0.3	20	−58.6±0.5	4.8±0.1	20	0.47±0.02	20	2.8±0.1	20	−149.4±17.5	20
**$Na_V1.6^{K1546TAG}$**	−11.5±0.8	−6.6±0.3	18	−55.8±0.6** (*p*= 0.0025)	4.9±0.1	17	0.57±0.03** (*p*=0.0026)	18	2.4±0.2* (*p*=0.0173)	18	−105.5±12.5	18

Notes: Data are presented as means±s.e.m.; *n*, number of recorded cells; * $p < 0.05$, ** $p < 0.01$, Student's *t*-test or Mann–Whitney *U* test.

Table S16. Comparison of the AIS mean fluorescence intensity (measured in the ATTO488 channel; shown in Fig. 5D) in neurons expressing control ($Na_V1.6^{K1546TAG,\ Y371C}$-HA) or one of the loss-of-function variants ($Na_V1.6^{K1546TAG,\ Y371C,\ I1652N}$-HA and $Na_V1.6^{K1546TAG,\ Y371C,\ T1785P}$-HA) using the independent-samples Kruskal–Wallis test.

Independent-Samples Kruskal-Wallis Test

Total N	79
Test Statistic	2.244[a,b]
Degree Of Freedom	2
Asymptotic Significance (2-sided test)	0.326

a. The test statistic is adjusted for ties.

b. Multiple comparisons are not performed because the overall test does not show significant differences across samples.

Table S17. Comparison of the $Na_V1.6^{K1425TAG}$–HA AIS fluorescence intensity (measured in the ATTO488 channel; shown in Fig. 6C) between transfected neurons and neurons transduced with different combinations of AAVs using the independent-samples Mann–Whitney *U* test.

Groups: 1. Transfection-only; 2. Transfection + AAV#7 and AAV#2; 3. Transfection + AAV#1 and AAV#2 (lower MOI); 4. Transfection + AAV#1 and AAV#2 (higher MOI).

Ranks

	Category	N	Mean Rank	Sum of Ranks
CorrMean	1	30	24.77	743.00
	2	28	34.57	968.00
	Total	58		

Test Statistics[a]

	CorrMean
Mann-Whitney U	278.000
Wilcoxon W	743.000
Z	-2.210
Asymptotic Significance (2-tailed)	0.027

a. Grouping Variable: category

Ranks

	Category	N	Mean Rank	Sum of Ranks
CorrMean	1	30	28.53	856.00
	3	22	23.73	522.00
	Total	52		

Test Statistics[a]

	CorrMean
Mann-Whitney U	269.000
Wilcoxon W	522.000
Z	-1.130
Asymptotic Significance (2-tailed)	0.259

a. Grouping Variable: category

Ranks

	Category	N	Mean Rank	Sum of Ranks
CorrMean	1	30	27.27	818.00
	4	29	32.83	952.00
	Total	59		

Test Statistics[a]

	CorrMean
Mann-Whitney U	353.000
Wilcoxon W	818.000
Z	-1.243
Asymptotic Significance (2-tailed)	0.214

a. Grouping Variable: Category

Table S18. Comparison of the $Na_V1.6^{K1425TAG}$–HA AIS fluorescence intensity (measured in the HA channel; shown in Fig. 6C) between transfected neurons and neurons transduced with different combinations of AAVs using independent-samples Mann–Whitney *U* test.

Groups: 1. Transfection-only; 2. Transfection + AAV#7 and AAV#2; 3. Transfection + AAV#1 and AAV#2 (lower MOI); 4. Transfection + AAV#1 and AAV#2 (higher MOI).

Ranks

	Category	N	Mean Rank	Sum of Ranks
CorrMean	1	30	28.97	869.00
	2	28	30.07	842.00
	Total	58		

Test Statistics[a]

	CorrMean
Mann-Whitney U	404.000
Wilcoxon W	869.000
Z	-0.249
Asymptotic Significance	0.803

a. Grouping Variable: category

Ranks

	Category	N	Mean Rank	Sum of Ranks
CorrMean	1	30	30.63	919.00
	3	22	20.86	459.00
	Total	52		

Test Statistics[a]

	CorrMean
Mann-Whitney U	206.000
Wilcoxon W	459.000
Z	-2.297
Asymptotic Significance (2-tailed)	0.022

a. Grouping Variable: category

Ranks

	category	N	Mean Rank	Sum of Ranks
CorrMean	1	30	31.73	952.00
	4	29	28.21	818.00
	Total	59		

Test Statistics[a]

	CorrMean
Mann-Whitney U	383.000
Wilcoxon W	818.000
Z	-0.788
Asymptotic Significance (2-tailed)	0.430

a. Grouping Variable: category

Table S19. Comparison of the $Na_V1.6^{K1546TAG}$–HA AIS fluorescence intensity (measured in the ATTO488 channel; shown in Fig. 6C) between transfected neurons and neurons transduced with different combinations of AAVs using the independent-samples Mann–Whitney *U* test.

Groups: 1. Transfection-only; 2. Transfection + AAV#7 and AAV#2; 3. Transfection + AAV#1 and AAV#2 (lower MOI); 4. Transfection + AAV#1 and AAV#2 (higher MOI).

Ranks

	Category	N	Mean Rank	Sum of Ranks
CorrMean	1	35	30.03	1051.00
	2	38	43.42	1650.00
	Total	73		

Test Statistics[a]

	CorrMean
Mann-Whitney U	421.000
Wilcoxon W	1051.000
Z	-2.694
Asymptotic Significance (2-tailed)	0.007

a. Grouping Variable: category

Ranks

	category	N	Mean Rank	Sum of Ranks
CorrMean	1	35	35.71	1250.00
	3	28	27.36	766.00
	Total	63		

Test Statistics[a]

	CorrMean
Mann-Whitney U	360.000
Wilcoxon W	766.000
Z	-1.798
Asymptotic Significance (2-tailed)	0.072

a. Grouping Variable: category

Ranks

	Category	N	Mean Rank	Sum of Ranks
CorrMean	1	35	30.57	1070.00
	4	29	34.83	1010.00
	Total	64		

Test Statistics[a]

	CorrMean
Mann-Whitney U	440.000
Wilcoxon W	1070.000
Z	-0.910
Asymptotic Significance (2-tailed)	0.363

a. Grouping Variable: category

Table S20. Comparison of the $Na_V1.6^{K1546TAG}$–HA AIS fluorescence intensity (measured in the HA channel; shown in Fig. 6C) between transfected neurons and neurons transduced with different combinations of AAVs using independent-samples Mann–Whitney *U* test.

Groups: 1. Transfection-only; 2. Transfection + AAV#7 and AAV#2; 3. Transfection + AAV#1 and AAV#2 (lower MOI); 4. Transfection + AAV#1 and AAV#2 (higher MOI).

Ranks

	Category	N	Mean Rank	Sum of Ranks
CorrMean	1	35	29.34	1027.00
	2	38	44.05	1674.00
	Total	73		

Test Statistics[a]

	CorrMean
Mann-Whitney U	397.000
Wilcoxon W	1027.000
Z	-2.959
Asymptotic Significance (2-tailed)	0.003

a. Grouping Variable: category

Ranks

	Category	N	Mean Rank	Sum of Ranks
CorrMean	1	35	35.74	1251.00
	3	28	27.32	765.00
	Total	63		

Test Statistics[a]

	CorrMean
Mann-Whitney U	359.000
Wilcoxon W	765.000
Z	-1.812
Asymptotic Significance (2-tailed)	0.070

a. Grouping Variable: category

Journal of Cell Science • Supplementary information

Ranks

	Category	N	Mean Rank	Sum of Ranks
CorrMean	1	35	31.71	1110.00
	4	29	33.45	970.00
	Total	64		

Test Statistics[a]

	CorrMean
Mann-Whitney U	480.000
Wilcoxon W	1110.000
Z	-.371
Asymptotic Significance (2-tailed)	0.711

a. Grouping Variable: category

Table S21. Pairwise comparisons of the periodic spacing of $Na_V1.6$ (shown in Fig. 7D) measured in click-labeled neurons using the independent-samples Kruskal–Wallis test.

Groups: 1. Mock-transfected cells; 2. WT; 3. K1425TAG; 4. K1546TAG.

Independent-Samples Kruskal-Wallis Test

Total N	109
Test Statistic	8.873[a]
Degree Of Freedom	3
Asymptotic Significance (2-sided test)	0.031

a. The test statistic is adjusted for ties.

Pairwise Comparisons

Comparisons	Test Statistic	Standard Error	Standard Test Statistic	Significance	Adjusted Significance
3-1	13.109	8.095	1.619	0.105	0.632
3-4	-21.287	8.560	-2.487	0.013	0.077
3-2	22.541	8.761	2.573	0.010	0.061
1-4	-8.178	8.497	-0.962	0.336	1.000
1-2	-9.432	8.699	-1.084	0.278	1.000
4-2	1.254	9.133	0.137	0.891	1.000

Notes. Each row tests the null hypothesis that the Sample 1 and Sample 2 distributions are the same. Asymptotic significances (2-sided tests) are displayed. The significance level is 0.050. a. Significance values have been adjusted by the Bonferroni correction for multiple tests.

Journal of Cell Science • Supplementary information

Table S22. Comparison of the degree of periodicity of $Na_V1.6$ (shown in Fig. 7E) measured in click-labeled neurons using the one-way ANOVA.

Groups: 1. Mock-transfected cells; 2. WT; 3. K1425TAG; 4. K1546TAG.

ANOVA

	Sum of Squares	Degree of Freedom	Mean Square	F	Significance
Between Groups	0.183	3	0.061	0.901	0.444[a]
Within Groups	7.119	105	0.068		
Total	7.302	108			

[a]*Although we did not observe an overall significant difference between groups which as such does not require post-hoc analysis, post-hoc Tukey's Honest Significant Difference (HSD) test was performed to provide further evidence that none of the pairwise comparisons showed a significant difference among groups.*

Tukey HSD

(I) category	(J) category	Mean Difference (I-J)	Standard Error	Significa-nce	95% Confidence Interval	
					Lower Bound	Upper Bound
1	2	-0.050109755960730	0.071659704464087	0.897	-0.237188459396637	0.136968947475178
	3	0.055964298387097	0.066687792398905	0.836	-0.118134462280310	0.230063059054504
	4	-0.036233403612903	0.069994845620378	0.955	-0.218965736432101	0.146498929206294
2	1	0.050109755960730	0.071659704464087	0.897	-0.136968947475178	0.237188459396637
	3	0.106074054347826	0.072166607046829	0.459	-0.082327996409381	0.294476105105033
	4	0.013876352347826	0.075233210293277	0.998	-0.182531538840741	0.210284243536393
3	1	-0.055964298387097	0.066687792398905	0.836	-0.230063059054504	0.118134462280310
	2	-0.106074054347826	0.072166607046829	0.459	-0.294476105105033	0.082327996409381
	4	-0.092197702000000	0.070513717404429	0.560	-0.276284629585331	0.091889225585331
4	1	0.036233403612904	0.069994845620378	0.955	-0.146498929206294	0.218965736432101
	2	-0.013876352347826	0.075233210293277	0.998	-0.210284243536393	0.182531538840741
	3	0.092197702000000	0.070513717404429	0.560	-0.091889225585331	0.276284629585332

Journal of Cell Science • Supplementary information

Table S23. Comparison of the periodic spacing of $Na_V1.6$ (shown in Fig. 8D) measured in click-labeled neurons using the independent-samples Kruskal–Wallis test.

Groups: 1. $paNa_V$; 2. HA-AF647; 3. AF647-Pyr-tz.

Independent-Samples Kruskal-Wallis Test

Total N	68
Test Statistic	4.507[a,b]
Degree Of Freedom	2
Asymptotic Significance (2-sided test)	0.105

a. The test statistic is adjusted for ties.

b. Multiple comparisons are not performed because the overall test does not show significant differences across samples.

Table S24. Comparison of the degree of periodicity of $Na_V1.6$ (shown in Fig. 8E) measured in click-labeled neurons using the independent-samples Kruskal–Wallis test.

Groups: 1. $paNa_V$; 2. HA-AF647; 3. AF647-Pyr-tz.

Independent-Samples Kruskal-Wallis Test

Total N	68
Test Statistic	0.335[a,b]
Degree Of Freedom	2
Asymptotic Significance (2-sided test)	0.846

a. The est statistic is adjusted for ties.

b. ultiple comparisons are not performed because the overall test does not show significant differences across samples.

Journal of Cell Science • Supplementary information

Table S25. Pairwise comparisons of the somatic mean fluorescence intensity (measured in the ATTO488 channel; shown in Fig. S6C) using the independent-samples Kruskal–Wallis test.

Groups: 1. $Na_V1.6^{WT}$-HA + TCO*A-Lys; 2. $Na_V1.6^{WT}$-HA no TCO*A-Lys; 3. $Na_V1.6^{WT}$-HA + NES $PylRS^{AF}/tRNA^{Pyl}$ + TCO*A-Lys; 4. $Na_V1.6^{WT}$-HA + NES $PylRS^{AF}/tRNA^{Pyl}$ no TCO*A-Lys; 5. $Na_V1.6^{K1546TAG}$-HA + NES $PylRS^{AF}/tRNA^{Pyl}$ + TCO*A-Lys; 6. $Na_V1.6^{K1546TAG}$-HA + NES $PylRS^{AF}/tRNA^{Pyl}$ no TCO*A-Lys; 7. $Na_V1.6^{K1425TAG}$HA + NES $PylRS^{AF}/tRNA^{Pyl}$ + TCO*A-Lys; 8. $Na_V1.6^{K1425TAG}$-HA + NES $PylRS^{AF}/tRNA^{Pyl}$ no TCO*A-Lys; 9. pcDNA3.1/Zeo(+) + NES $PylRS^{AF}/tRNA^{Pyl}$ + TCO*A-Lys; 10. pcDNA3.1/Zeo(+) + NES $PylRS^{AF}/tRNA^{Pyl}$ no TCO*A-Lys; 11. untransfected cells + TCO*A-Lys; 12. untransfected cells no TCO*A-Lys.

Independent-Samples Kruskal-Wallis Test

Total N	148
Test Statistic	75.111[a]
Degree Of Freedom	11
Asymptotic Significance (2-sided test)	<0.001

[a]The test statistic is adjusted for ties.

Pairwise Comparisons

Comparisons	Test Statistic	Standard Error	Standard Test Statistic	Significance	Adjusted Significance[a]
6-12	-6.000	17.501	-0.343	0.732	1.000
6-8	-9.333	17.501	-0.533	0.594	1.000
6-10	-25.167	17.501	-1.438	0.150	1.000
6-11	-30.833	17.501	-1.762	0.078	1.000
6-4	39.500	17.501	2.257	0.024	1.000
6-9	-41.583	17.501	-2.376	0.017	1.000
6-2	51.827	17.161	3.020	0.003	0.167
6-1	60.932	17.894	3.405	<0.001	0.044
6-3	65.519	17.161	3.818	<0.001	0.009
6-7	-90.250	16.864	-5.352	<0.001	0.000
6-5	96.981	17.161	5.651	<0.001	0.000
12-8	3.333	17.501	0.190	0.849	1.000
12-10	19.167	17.501	1.095	0.273	1.000
12-11	24.833	17.501	1.419	0.156	1.000
12-4	33.500	17.501	1.914	0.056	1.000
12-9	35.583	17.501	2.033	0.042	1.000
12-2	45.827	17.161	2.670	0.008	0.500
12-1	54.932	17.894	3.070	0.002	0.141
12-3	59.519	17.161	3.468	<0.001	0.035
12-7	84.250	16.864	4.996	<0.001	0.000

Journal of Cell Science • Supplementary information

12-5	90.981	17.161	5.302	<0.001	0.000
8-10	-15.833	17.501	-0.905	0.366	1.000
8-11	-21.500	17.501	-1.229	0.219	1.000
8-4	30.167	17.501	1.724	0.085	1.000
8-9	-32.250	17.501	-1.843	0.065	1.000
8-2	42.494	17.161	2.476	0.013	0.876
8-1	51.598	17.894	2.884	0.004	0.260
8-3	56.186	17.161	3.274	0.001	0.070
8-7	80.917	16.864	4.798	<0.001	0.000
8-5	87.647	17.161	5.107	<0.001	0.000
10-11	-5.667	17.501	-0.324	0.746	1.000
10-4	14.333	17.501	0.819	0.413	1.000
10-9	16.417	17.501	0.938	0.348	1.000
10-2	26.660	17.161	1.554	0.120	1.000
10-1	35.765	17.894	1.999	0.046	1.000
10-3	40.353	17.161	2.351	0.019	1.000
10-7	65.083	16.864	3.859	<0.001	0.008
10-5	71.814	17.161	4.185	<0.001	0.002
11-4	8.667	17.501	0.495	0.620	1.000
11-9	10.750	17.501	0.614	0.539	1.000
11-2	20.994	17.161	1.223	0.221	1.000
11-1	30.098	17.894	1.682	0.093	1.000
11-3	34.686	17.161	2.021	0.043	1.000
11-7	59.417	16.864	3.523	<0.001	0.028
11-5	66.147	17.161	3.855	<0.001	0.008
4-9	-2.083	17.501	-0.119	0.905	1.000
4-2	12.327	17.161	0.718	0.473	1.000
4-1	21.432	17.894	1.198	0.231	1.000
4-3	26.019	17.161	1.516	0.129	1.000
4-7	-50.750	16.864	-3.009	0.003	0.173
4-5	-57.481	17.161	-3.350	<0.001	0.053
9-2	10.244	17.161	0.597	0.551	1.000
9-1	19.348	17.894	1.081	0.280	1.000
9-3	23.936	17.161	1.395	0.163	1.000
9-7	48.667	16.864	2.886	0.004	0.258
9-5	55.397	17.161	3.228	0.001	0.082
2-1	9.105	17.562	0.518	0.604	1.000
2-3	-13.692	16.814	-0.814	0.415	1.000

Journal of Cell Science • Supplementary information

2-7	-38.423	16.511	-2.327	0.020	1.000
2-5	-45.154	16.814	-2.685	0.007	0.478
1-3	-4.587	17.562	-0.261	0.794	1.000
1-7	-29.318	17.272	-1.697	0.090	1.000
1-5	-36.049	17.562	-2.053	0.040	1.000
3-7	-24.731	16.511	-1.498	0.134	1.000
3-5	-31.462	16.814	-1.871	0.061	1.000
7-5	6.731	16.511	0.408	0.684	1.000

Notes. Each row tests the null hypothesis that the Sample 1 and Sample 2 distributions are the same. Asymptotic significances (2-sided tests) are displayed. The significance level is 0.050. a. Significance values have been adjusted by the Bonferroni correction for multiple tests.

Table S26. Average photon counts and average localization precision for dSTORM imaging of click-labeled NF186, click-labeled Na_V1.6, and immunolabeled Na_V1.6.

**Fig. 2H: dSTORM imaging of click-labeled NF186-HA**				
	Average photon count		Average localization precision	
	Mean	Standard Error	Mean	Standard Error
panNF	5108.61	512.81	6.01	0.48
HA-AF647	5724.74	298.46	6.05	0.41
AF647-Pyr-tz	5577.317	263.44	5.81	0.24
**Fig. 7A: dSTORM imaging of immunostained Na_V1.6-HA**				
	Average photon count		Average localization precision	
	Mean	Standard Error	Mean	Standard Error
panNa_V	4506.87	418.94	5.90	0.46
WT	5529.43	490.75	5.13	0.37
K1425TAG	5003.46	233.40	4.91	0.21
K1546TAG	5252.21	504.48	5.41	0.43
**Fig. 8A: dSTORM imaging of click-labeled Na_V1.6-HA**				
	Average photon count		Average localization precision	
	Mean	Standard Error	Mean	Standard Error
panNa_V	1804.52	28.29	8.63	0.18
HA-AF647	2328.06	25.33	7.31	0.11
AF647-Pyr-tz	2346.00	63.54	8.86	0.30

Journal of Cell Science • Supplementary information

Table S27. Mutagenesis and cloning primers.

Purpose	Primer name	Primer sequence 5'→3'
Deletion of HA tag from the N terminus of NF186	HA(del)-NF186fw	GCCATTGAGATTCCGATGGATCCAAGCATTCAGAATGAG
	HA(del)-NF186rv	CTCATTCTGAATGCTTGGATCCATCGGAATCTCAATGGC
NF186^{TAG} mutagenesis	NF186^{K519TAG}fw	GAGGTCTAGGACCCCACCAGGATCTACAGGATG
	NF186^{K519TAG}rv	GGGGTCCTAGACCTCCAGGCGGACTTGATTTTCA
	NF186^{K534TAG}fw	GTGGCCTAGAGGGGCACCACAGTGCAG
	NF186^{K534TAG}rv	GCCCCTCTAGGCCACCTGGTCTTCAGGC
	NF186^{K571TAG}fw	AGGATGTAGAAGGAAGATGACTCCCTGACCATCTTCG
	NF186^{K571TAG}rv	TTCCTTCTACATCCTGTTTCCAATGTAGAGTGGCTC
	NF186^{K604TAG}fw	CTGGCATAGGCCTACCTCACTGTTCTAGCTGATCAG
	NF186^{K604TAG}rv	GTAGGCCTATGCCAGGTCCTGGTCCAG
	NF186^{K680TAG}fw	CACTCCTAGTTCCCAGGCAGTGTCAACTCAG
	NF186^{K680TAG}rv	TGGGAACTAGGAGTGGTCATGCCAGACTCCT
	NF186^{K809TAG}fw	TTTGGGTAGGGCCCGGAGCCTGAAAC
	NF186^{K809TAG}rv	CGGGCCCTACCCAAAGTCATTTTCAGCCTGGACTC
Addition of HA tag to the C terminus of NF186	NF186-HAfw	GGTGGTGGGCCCTGAAGACCCCAAAGAAG
	NF186-HArv	ACCACCGCGGCCGCTCAAGCGTAGTCTGGGACGTCGTATG GGTAGGCCAGGGAATAGATGGCATTGACTG
Cloning of hNSE into NF186 plasmid	hNSE(AseI)fw	GGTGGTATTAATTGTATGCAGCTGGACCTAGGAGAG AAG
	hNSE(BglII)rv	ACCACCAGATCTCGGTGGTAGTGGCGG
Cloning of *mScn1b* into pACEMam2 to make multigene plasmid	*mScn1b*(KpnI) rv	GGTGGTGCTAGCCACCATGGGGACGCTGCTGGCTCT
	mScn1b(NheI)fw	ACCACCGGTACCTTATTCAGCCACCTGGACGCCT
Cloning of *mScn2b* into pMDS to make multigene plasmid	*mScn2b*(NheI)fw	GGTGGTGCTAGCCACCATGGAAGTCACAGCGCCCACC A
	m*Scn2b*(KpnI)rv	ACCACCGGTACCTTACTTGGTGCCATCTTCCGCGTTG
Cloning of mGFP into pMDC to make multigene plasmid	mGFP(BamHI)fw	GGTGGTGGATCCCACCATGGTGAGCAAGGGCG
	mGFP(XbaII)rv	ACCACCTCTAGATTACTTGTACAGCTCGTCCATGCCGA
Cloning of *mScn1b* and *mScn2b* for generation of N1E-115-1$^{\beta1\beta2}$	*mScn1b*(NheI)fw	GGTGGTGCTAGCCACCATGGGGACGCTGCTGGCTCT
	mScn1b(NotI)rv	CCGTCCGCGGCCGCCTATTCAGCCACCTGGACGCC
	mScn2b(NheI)fw	GGTGGTGCTAGCCACCATGCACAGGGATGCCTGGCTACC
	mScn2b(NotI)rv	CCGTCCGCGGCCGCTTACTTGGTGCCATCTTCCGCGT
Amplification of *mScn1b* and *mScn2b*	*mScn1b*rv	CTATTCAGCCACCTGGACGCCTG
	*mScn2b*rv	TTACTTGGTGCCATCTTCCGCGTTG
	PiggyBACfw	ATGTAATTACGTCCCTCCCCCGCTAG

Journal of Cell Science • Supplementary information

from N1E-115-1$^{\beta1\beta2}$ gDNA		
Loss-of-function *Scn8a* mutagenesis	*mScn8a*^{I652N}rv	CAGCAGGCCGTTATTGAACAGGGCTGGCAG
	mScn8a^{I652N}fw	CTGCCAGCCCTGTTCAATAACGGCCTGCTG
	mScn8a^{T1785P}rv	CCAGATCTCATAGAAGGGCTCGAAGTCATCCTCAG
	mScn8a^{T1785P}fw	CTGAGGATGACTTCGAGCCCTTCTATGAGATCTGG
Introduction of TTXr mutation into *mScn8a*	*mScn8a*^{Y371C}fw	GTACAGGTTCTCCCAGCAGTCCTGGGTCATCAG
	mScn8aA^{Y371C}rv	CTGATGACCCAGGACTGCTGGGAGAACCTGTAC
Addition of HA tag on the C terminus of mouse $Na_V1.6$	*mScn8a*-HAfw	(Phos)CTAGATACCCATACGACGTCCCAGACTACGCTTAAGGGCC
	mScn8a-HArv	(Phos)CTTAAGCGTAGTCTGGGACGTCGTATGGGTAT
Measurement of the AAV titer via ddPCR	GFPfw	ATCTTCTTCAAGGACGACG
	GFPrv	TCCTCCTTGAAGTCGATGC
	Probe	FAM-ACGACGGCAACTACA-BHQ1

Journal of Cell Science • Supplementary information
