# Supplementary material for: Direct fluorescent labeling of NF186 and NaV1.6 in living primary neurons using bioorthogonal click chemistry
Source: J Cell Sci. 2023 Jun 28;136(12):jcs260600. doi: 10.1242/jcs.260600 (PMC10323244; doi:10.1242/jcs.260600)
Supplement: Supplementary file 1 [file joces-136-260600_review_history.pdf]

Direct fluorescent labeling of NF186 and $Na_V1.6$ in living primary neurons using bioorthogonal click chemistry

Nevena Stajković, Yuanyuan Liu, Aleksandra Arsić, Ning Meng, Hang Lyu, Nan Zhang, Dirk Grimm, Holger Lerche and Ivana Nikić-Spiegel

DOI: 10.1242/jcs.260600

Editor: Giampietro Schiavo

Review timeline

Submission to Review Commons:	4th March 2022
Submission to Journal of Cell Science:	1 September 2022
Editorial decision:	8 September 2022
First revision received:	22 May 2023
Accepted:	26 May 2023

Reviewer 1

Evidence, reproducibility and clarity

AIS organization is highly complex and unique and AIS labeling in living cells has been problematic. In this study, authors comprehensively searched and optimized live cell markers for AIS.The main advantage of the labeling approach is that the small fluorescent dye is directly attached to the proteins of interest. Therefore, the protein of interest is modified in a minimally invasive way.

Significance

I was first wondering whether tool development is enough important to be published as such but here, the development and testing of the constructs, whether they affect cell functionality, is very comprehensive. This all makes this tool very useful for other researchers. I can take the method directly to use and I don't need to do all testing by myself. I feel that this tool development takes field forward.

Own expertise: I have personally gone through the AIS live cell labeling problems and therefore I can easily appreciate the work done here.

Reviewer 2

Evidence, reproducibility and clarity

Summary:

This study proposes a novel tool for AIS live and fixed labelling based on biorthogonal click chemistry. Stajkovic and colleagues used this method to specifically label the AIS proteins NF186 and Nav1.6 of mouse and rat neurons, and did a thorough process of optimization to get convincing results. The authors considered different promoters, transfection strategies and the use of AAVs to get the most efficient labelling strategy for both proteins. They have also gone through a strong validation process based on transfection efficiency, quality of staining, potential effects on AIS length and nanostructure, and electrophysiological properties. Finally, Stajkovic

© 2023. Published by The Company of Biologists under the terms of the Creative Commons Attribution License (https://creativecommons.org/licenses/by/4.0/).

and colleagues used this tool to study how two epilepsy-causing Nav1.6 mutant variants affect AIS function, providing interesting data to the understanding of this pathology. In summary, this method convincingly overcomes some well-described issues associated with pre-existing AIS live cell labelling tools by being minimally "invasive" to the proteins of interest.
Besides the scientific content, another strong point of this article is the clarity of the manuscript and the figures: the presence of schematics (i.e. Fig. 1) and the detailed description of experiments and results will help non-specialist readers to follow the study.

I strongly recommend this article for journal publication.

Major comments:

I have no major comments

Minor comments:

I have some minor comments:

- On lines 107 and 108, the sentence "The C-terminal HA-tag allowed us to detect the full- length NF-186 protein by immunostaining it with an anti-HA antibody" would have a better place just after lines 104-105 " [...] we modified the previously described plasmid (Zhang et al., 1998) by moving the hemagglutinin (HA) tag from the N terminus to the C terminus".
- Fig.2b: the AnkG staining looks substantially longer than that showed in c. However, the results on AIS length show no significant changes in between the groups. This is visually misleading, the authors should choose a picture for the WT construct that is representative of the data.
- Line 238: what is the rationale behind choosing these cells? For example, have they been used in other studies for similar purposes? If so, please provide the reference.
- Figure 3c, the authors omitted the comparison with the WT construct this time, as opposed to the neurofascin experiments. What is the reason?
- Fig. 4: why did the authors chose these cells for electrophysiology experiments and not neurons? Explain the rationale in the text or, alternatively, cite similar studies using the same tool.
- Fig.4, biophysical properties: did the authors find differences in passive properties? Measures of resting potential, membrane resistance and cell capacitance should be reported.
- Fig 4, STORM images. The periodic distribution of the dots should be enhanced with some sort of arrows or lines, for the non-specialist audience.
- Line 374: rat or mouse primary neurons?

Referees cross-commenting

I fully agree with the following remarks from Reviewers #3, #4 and #5. This is a point that I have raised in my report too. The authors need better images to show the periodicity visualization, and a quantification would be of great benefit to support the claim with numbers (and how these compare to similar studies in the literature):

R3: 2. For the dSTORM analysis of the tagged Nav1.6 protein, I also cannot tell there is periodic organization from the image directly. Some analysis is needed there.
R4: 2."As there was no obvious difference in the nanoscale organization of the NaV1.6WT 317 -HA or NaV1.6TAG 318 -HA channels (Fig. 4. e-g), these experiments confirmed that the NaV1.6 overexpression, TCO*A319 Lys incorporation, and click labeling did not affect the nanoscale periodic organization of the sodium channels in the AIS." It is clearly noticeable that for WT, the spot density is more compared to the other two mutants. Why is that so? Using cluster analysis, one can quantify spot density and discuss nanoscale organization quantitatively. The author should quantify the periodicity and compare it among different variants and with previous reports.
R5: 3. The authors claim that there was no obvious difference in the nanoscale organization of the NaV1.6WT 317 -HA or NaV1.6TAG 318 -HA channels (Fig. 4. e-g), but it is hard to conclude this without any quantification and statistical analysis. Sodium channels have been shown to be associated with the membrane-associated periodic skeleton structures in neurons and average

© 2023. Published by The Company of Biologists under the terms of the Creative Commons Attribution License (https://creativecommons.org/licenses/by/4.0/).

autocorrelation analysis has been developed to quantify the degree of periodicity of such structural organizations (Han et al. PNAS 114(32)E6678-E6685, 2017). The authors should use this approach to quantify and compare the average autocorrelation amplitudes.

I also agree with these comments from Reviewers #3 and #5:

R3: 4. It is unclear, for all the presented data, whether all the cells are collected from a single biological replicate or from multiple replicates. At least 2-3 replicates are needed to see the reproducibility in terms of labeling efficiency, and other related conclusions.
R5: 1. The authors should indicate how many replicates were performed and how many cells were analyzed for each experiment.

Significance

The proposed tool in this article represents a big step forward in the field of AIS live cell imaging. As stated by the authors in the introduction, previous studies have described methods based on tagging fluorescent proteins to the protein of interest or labelling the extracellular part of proteins with antibodies. The same studies reported several issues: the interference with important domains of the protein due to the size and the position of the tag in the case of fluorescent proteins (Dumitrescu et al., 2016, PMID: 27932952; Dzhashiashvili et al., 2007, PMID: 17548513), or the failure to report plasticity changes in the AIS in the case of antibodies (Dumitrescu et al., 2016, PMID: 27932952).

This tool can be useful for research teams aiming to understand, for example, the live development of the AIS or understanding the trafficking of its proteins. The authors have applied this method to two transmembrane proteins (NF186 and Nav1.6), but as they state in their discussion, it will be useful to tag other candidates, including cytoplasmic proteins. One of the main problems of immunocytochemistry is to find the right antibody to detect your protein. Sometimes, absence of proof is not proof of absence: just because the protein is not detected via immunostaining does not mean that the protein is not expressed there. This tool offers an alternative to these challenging scenarios.

My expertise keywords: axon initial segment, neuronal polarity, axon biology, super resolution microscopy.

Reviewer 3

Evidence, reproducibility and clarity

The manuscript by Stajkovic et al the describes step-wise generation and validation of the fluorescent labeling of NF186 and Nav1.6 in primary neurons by non-natural amino acid and click chemistry. For each protein of interest, the authors started by generating constructs carrying amber codon at different positions, and then selected for the best construct(s) by judging (1) the labeling efficiency, (2) whether the particular labeling position affect the function of the protein, and (3) whether the labeled protein shows any mislocalization. During the trouble shooting process, the authors also introduced adeno-associated viral (AAV) vectors for more efficiently delivering constructs into the cells. The method described in the manuscript could become a reference for researchers who aim to label similar neuronal proteins.

Specific comments:

1. There is some patch-like background from the 488 channel from the click reaction, some of which have very as strong signal as the staining on the neurons. What is the potential cause for this? With immunostaining on HA, the background doesn't affect too much on the image data interpretation. However, the major goal of this method development is to use it in live-cell without immunostaining. Without another reference, the high background might cause issues in data interpretation. Can the author also suggest way to avoid or lower this in the discussion?
2. For the dSTORM analysis of the tagged Nav1.6 protein, I also cannot tell there is periodic

© 2023. Published by The Company of Biologists under the terms of the Creative Commons Attribution License (https://creativecommons.org/licenses/by/4.0/).

organization from the image directly. Some analysis is needed there.
3. The authors use the AIS length as a parameter to evaluate the function of the clickable mutant of NF186, and using patch clamp for functional validation of the clickable mutant of Nav1.6. In both cases, the comparison is done between the mutant and the WT construct, but both in transfected cell and exogenously expressed. It's also worth comparing with untransfected cells as the true native situation.
4. It is unclear, for all the presented data, whether all the cells are collected from a single biological replicate or from multiple replicates. At least 2-3 replicates are needed to see the reproducibility in terms of labeling efficiency, and other related conclusions.
5. One application presented in this manuscript is to evaluate the effect of epilepsy-causing mutations of Nav1.6. By comparing the intensity of ATTO488, the result suggests that there is no significant impact of these mutations on membrane tracking. I am wondering if the author should study the membrane tracking by also looking at the diffusion in live-cell with the labeling method. The comparison of the intensity only can be achieved by just immunostaining. It doesn't really demonstrate the benefit of live-cell labeling and imaging with the presented method.

Significance

The data itself is mostly convincing, however, I do not see much novelty from this manuscript. Both the labeling method using non-natural amino acid and click chemistry and AAV delivery are established. However, I can see that for research groups who specifically interested in studying these two proteins or proteins closed related, the results from this manuscript could be of direct help.

Reviewer 4

Evidence, reproducibility and clarity

The manuscript demonstrates a novel method of labeling two large components of the initial axon segment, neurofascin (NF186) and Nav1.6 using unnatural amino acids and click chemistry in live cells. They have applied their method for epilepsy causing two Nav1.6 variants without affecting their functionality. Since these proteins are larger in size, selecting the labeling sites and transfection efficiency become critical factors. They have targeted different lysine sites and shown the best performing labeling site. Also, they have developed a viral vector to improve transfection efficiency.

The experiments are well designed, and the manuscript is nicely written. In my opinion, the manuscript can be accepted, but the author should address the following comments.

Major comments

1. "Confocal microscopy revealed that the hNSE promoter lowered the WT and clickable NF186-HA expression levels and consequently improved the localization of these proteins." Is the lower expression level a measure of localization improvement? How does the author conclude that the localization has improved?
2. "As there was no obvious difference in the nanoscale organization of the NaV1.6WT 317 - HA or NaV1.6TAG 318 -HA channels (Fig. 4. e-g), these experiments confirmed that the NaV1.6 overexpression, TCO*A319 Lys incorporation, and click labeling did not affect the nanoscale periodic organization of the sodium channels in the AIS." It is clearly noticeable that for WT, the spot density is more compared to the other two mutants. Why is that so? Using cluster analysis, one can quantify spot density and discuss nanoscale organization quantitatively. The author should quantify the periodicity and compare it among different variants and with previous reports.

Minor comments

1. "Although NF186K809TAG 158 -HA (Supplementary Fig. 4) showed bright click labeling, we excluded it from the analysis due to its frequent ectopic expression along the distal axon." How

© 2023. Published by The Company of Biologists under the terms of the Creative Commons Attribution License (https://creativecommons.org/licenses/by/4.0/).

frequently is this bright click labeling observed for this mutation? Is it not observed for other mutations at all? The authors should state this point clearly with some statistics.
2. "Immunostaining with anti-HA antibody revealed that the expression of NaV1.6WT 239 - HA on the membrane of the N1E-115-1 cells was higher than on the ND7/23 cells (Supplementary Fig. 5a-c). However, click labeling of both NaV1.6K1425TAG 240 -HA and NaV1.6K1546TAG 241 -HA with ATTO488-tz was not successful (Supplementary fig. 5d) indicating insufficient expression of the clickable constructs." Is this due to insufficient expression level or accessibility? The author should make this statement clear.
3. Authors should clearly state the drift correction procedure of 3D STORM data. What are the localization precision and photon count for 3D STORM experiments?
4. "Click labeling of NaV1.6 channels in living primary neurons" What kind of primary neurons have been used for click labeling of NaV1.6 channels? Is there any specific reason why authors have chosen cortical neurons for labeling NF186? Does this labeling strategy depend on primary neuron type?

Significance

Although the use of unnatural amino acids and click chemistry for labelling has been shown before from the same group, labelling large proteins, especially ion channels, without affecting their function is always challenging because of the accessibility of the labelling site as well as poor transfection efficiency. Here, they have selected two such large essential proteins: NF186 and Nav1.6, which are associated with epilepsy, and developed a method for fluorophore labelling with minimal perturbation. Other approaches namely using fluorescent proteins, biotin-streptavidin chemistry and halo-tag have been reported before to label these proteins, but these have a strong impact on their mislocalisation and perturbing their functionality. Therefore, this method will be of great importance in the field of studying these proteins.

Expertise: Live-cell confocal and multi-photon microscopy imaging, Super-resolution microscopy imaging, Live-cell labelling, and Amyloid aggregations

Reviewer 5

Evidence, reproducibility and clarity

Summary:

In this manuscript, Nevena Stajković et al. present a method for live labeling of the proteins localized at the axon initial segment (AIS) of cultured neurons using unnatural amino acids (UAAs) carrying strained alkenes and click chemistry. Using this method, the authors showed the successful labeling of two AIS-localized proteins, the 186 kDa isoform of neurofascin (NF186) and the 260 kDa voltage-gated sodium channel (NaV1.6). The authors also showed the transduction of neurons using adeno-associated viruses (AAVs) had higher efficiency than transfection by lipofectamine in delivering the vectors expressing required components for the click labeling.

Major comments:

1. Throughout the manuscript, only one representative image containing one AIG is shown for each condition without statistics and quantifications, so the conclusions are not sufficiently convincing. For example, in Fig. 1b, c, e; Fig. 2b,c,d,e; Fig. 3b,c,d,e ; Fig. 5c; and Supplementary Fig.1-6, the authors should quantify the average fluorescence intensities both for HA immunostaining and ATTO488-tz labeling in different conditions, as well as the labeling ratios (fluorescence intensity ratios between ATTO488 and AF647/AF555) . Without statistics and quantifications, it is unclear whether there is any significant difference between the constructs with different TAG positions, or between different transfection methods (e.g., lipofectamine 2000 vs 3000).

2. The only quantification done was for the average AIS length, but the statistical tests should be preformed between different conditions and the corresponding P values should be provided. It

© 2023. Published by The Company of Biologists under the terms of the Creative Commons Attribution License (https://creativecommons.org/licenses/by/4.0/).

seems that the transfected neurons generally have a longer AIS length than the transfected neurons (Fig. 2d and 3f). Could the authors provide an explanation for this?

3. The authors claim that there was no obvious difference in the nanoscale organization of the NaV1.6WT 317 -HA or NaV1.6TAG 318 -HA channels (Fig. 4. e-g), but it is hard to conclude this without any quantification and statistical analysis. Sodium channels have been shown to be associated with the membrane-associated periodic skeleton structures in neurons and average autocorrelation analysis has been developed to quantify the degree of periodicity of such structural organizations (Han et al. PNAS 114(32)E6678-E6685, 2017). The authors should use this approach to quantify and compare the average autocorrelation amplitudes.

4. The authors should also obtain dSTORM images for the click labeled neurons to demonstrate if the click labeling method would provide sufficient labeling efficiency for dSTORM, compared to immunostaining (HA and Ankyrin G immunostaining).

5. It seems that the click labeling has a off-target/background labeling in the soma of the neuron (see Fig. 3c,d. Could the authors quantify and determine the sources of such off-target labeling?

Minor comments:

1. The authors should indicate how many replicates were performed and how many cells were analyzed for each experiment.
2. The display range (i.e., intensity scale bar) was indicated only for a small portion of the fluorescence images. It is better to be consistent and show the display range for all images presented.

Significance

Unnatural amino acid (UAA)-based minimal tags for live-cell protein labeling in mammalian cells were invented about ten years ago (Lang et al., 2012b, Lang et al., 2012a, Nikic et al., 2014, Plass et al., 2012, Uttamapinant et al., 2015), and these authors recently introduced this labeling method to label live cultured neurons (Arsić et al., 2022). Therefore, it is unclear whether the method present in this manuscript has any significant advance compared to the Arsić et al. paper, given that the major difference between the two papers is that in the current manuscript, AIS localized proteins were labeled, whereas in the Arsić et al. paper, neurofilaments were labeled in the neurons. Therefore, the method presented in the current manuscript does not provide much novelty or technical advance compared to what has been described in the Arsić et al. paper.

My expertis is super-resolution flurescence imaging, cell labeling methods, and neurobiology.

Author response to reviewers' comments

Manuscript number: RC-2022-01341

Corresponding author(s): Ivana, Nikić-Spiegel

1. General Statements

We would like to thank the reviewers for their insightful and useful comments about our manuscript. Based on these comments and as outlined in our revision plan, we plan to strengthen our findings by performing new experiments and quantitative analyses. This particularly applies to our nanoscale (dSTORM) imaging dataset which was discussed by multiple reviewers.

© 2023. Published by The Company of Biologists under the terms of the Creative Commons Attribution License (https://creativecommons.org/licenses/by/4.0/).

We also appreciate the reviewers' overall positive evaluation of the significance of our labeling method for the axon initial segment studies. With regards to this, we would like to highlight that this manuscript particularly addresses the labeling of "difficult-to-label" neuronal proteins, such as large ion channels and transmembrane proteins. Although we and another group have recently reported click labeling of neurofilament light chain (PMID: 35031604) and AMPAR regulatory proteins (PMID: 34795271) in primary neurons, both of these proteins have a small size between ~30-68 kDa and compared to larger ion channels/transmembrane proteins are "easier" to express in primary neurons. The novelty in the current manuscript is that we successfully applied this method for the labeling of large and spatially restricted AIS components, such as NF186 and Nav1.6 (186 and 260 kDa, respectively). As some of the reviewers also pointed out, the size and complexity of these proteins makes labeling of the AIS rather challenging. We also used our approach to study the localization of epilepsy-causing Nav1.6 variants and could exclude the retention in the cytoplasm as a possible cause of their loss of function. Finally, we improved the efficiency of genetic code expansion in primary neurons by developing AAV-based viral vectors. Although AAVs are routinely used for gene delivery to neurons, AAVs for click-based labeling need to encode multiple components of the orthogonal translational machinery for genetic code expansion. By trying different promoters and gene combinations, we developed several variants that enable high efficiency of the genetic code expansion in neurons. On their own, these findings will facilitate further genetic code expansion and click chemistry studies, beyond the labeling of the axon initial segment.

2. Description of the planned revisions

Reviewer #2

- On lines 107 and 108, the sentence "The C-terminal HA-tag allowed us to detect the full-length NF-186 protein by immunostaining it with an anti-HA antibody" would have a better place just after lines 104-105 " [...] we modified the previously described plasmid (Zhang et al., 1998) by moving the hemagglutinin (HA) tag from the N terminus to the C terminus".

OUR RESPONSE: We will modify the text as the reviewer suggested.

- Fig.2b: the AnkG staining looks substantially longer than that showed in c. However, the results on AIS length show no significant changes in between the groups. This is visually misleading, the authors should choose a picture for the WT construct that is representative of the data.

OUR RESPONSE: We thank the reviewer for bringing this up. We will replace the panel in Fig.2b with a more representative image of NF186 WT construct in the revised version of the manuscript.

- Line 238: what is the rationale behind choosing these cells? For example, have they been used in other studies for similar purposes? If so, please provide the reference.

OUR RESPONSE: We initially probed neuroblastoma ND7/23 which are commonly used for the electrophysiological recordings of recombinant Nav1.6 (PMID: 30615093, 22623668, 25874799, 27375106). Although we were able to record Na^+ currents in those cells, only a small portion of channels was detected on the cell surface by microscopy (Suppl. Fig. 5a). As we discuss in the manuscript (lines 237-240), we then switched to N1E-115-1 cells in which we obtained a higher level of expression of the recombinant $Na_V1.6$ channels on the cell surface (Suppl. Fig. 5b). These cells have also been previously used for the electrophysiological studies of voltage-gated sodium channels, including Nav1.6 (PMID: 8822380, 24077057). We will modify the text and include these references in the revised manuscript.

- Figure 3c, the authors omitted the comparison with the WT construct this time, as opposed to the neurofascin experiments. What is the reason?

OUR RESPONSE: As shown by others (PMID: 31900387) and us in this manuscript, one of the main issues with the expression of the recombinant NF186 in neurons was that overexpression led to mislocalization of NF186 in neuronal soma and processes. This was particularly true for WT construct and certain amber mutants (e.g. K809TAG). Based on previous reports (PMID: 31900387), we then tested a weak human neuron-specific enolase promoter. This reduced expression level and

© 2023. Published by The Company of Biologists under the terms of the Creative Commons Attribution License (https://creativecommons.org/licenses/by/4.0/).

improved localization of NF186. However, since we still observed some neurons with mislocalized NF186 WT even with the enolase promoter, we found it important to quantitively compare the AIS length of WT construct and amber mutants to surrounding untransfected cells. On the other hand, since we did not have overexpression and mislocalization problem with Nav1.6 WT construct (all observed neurons have signal localizing in the AIS), we measured only the AIS length of the amber mutants. However, to avoid any confusion, we will also measure the AIS size of the neurons expressing Nav1.6 WT construct and compare it to surrounding cells and amber mutants. For this, we will need to perform new experiments and acquire new images. We will include the data in the revised manuscript.

- Fig. 4: why did the authors chose these cells for electrophysiology experiments and not neurons? Explain the rationale in the text or, alternatively, cite similar studies using the same tool.

OUR RESPONSE: Due to the branched neuronal processes which cause the space clamp problem in voltage clamp experiments with neurons, round and none-branching cells are frequently used to examine the biophysical properties of ion channels, including Nav1.6. By far, most of studies investigating the biophysical properties of $Na_V1.6$ channels were performed in neuroblastoma cells e.g. ND7/23 and N1E-115-1 cells (PMID: 25874799; 25242737). We tested these two types of cells and found that N1E-115-1 cells supported higher expression level of the recombinant $Na_V1.6$ channels on the cell surface than the ND7/23 cells (Suppl. Fig 5). Hence, N1E-115-1 were more suitable to get robust and reliable recordings (as we also discuss above in the response to reviewer's comment). We will clarify this in the revised manuscript.

- Fig.4, biophysical properties: did the authors find differences in passive properties? Measures of resting potential, membrane resistance and cell capacitance should be reported.

OUR RESPONSE: Passive properties such as resting membrane potential and membrane resistance are important functional features in neurons measured in current clamp experiments, but not applicable for ND7/23 and N1E-115-1 cells used in our voltage clamp experiments. To measure the Na^+ current mediated by WT or mutant $Na_V1.6$ channels expressed in N1E-115-1 cells, the endogenous Na^+ channels were blocked by tetrodotoxin and the endogenous K^+ channels were blocked by tetraethylammonium chloride, CsCl and CsF in extracellular and intracellular solutions. Under these conditions, resting potential and membrane resistance are not relevant for experiments. Cell capacitance reflects the size of the cell surface area, which can affect the number of channels expressed on the cell surface. To eliminate the effect of different cell sizes, Na^+ current densities normalized by cell capacitances were used in our experiments. We will report on these values in the revised manuscript.

- Fig 4, STORM images. The periodic distribution of the dots should be enhanced with some sort of arrows or lines, for the non-specialist audience.

OUR RESPONSE: Based on the comments from multiple reviewers, we plan to obtain additional dSTORM images of the neurons expressing recombinant Nav1.6 WT or amber mutants. We also intend to improve the visualization of these results by updating/modifying existing figures and including quantitative data.

- Line 374: rat or mouse primary neurons?

OUR RESPONSE: We are here referring to both, rat and mouse neurons. The images shown in Fig. 06 and Suppl. Fig. 08 were obtained from rat cortical neurons expressing Nav1.6 or fluorescent reporter. However, we were also able to successfully transduce mouse neurons with AAV92A carrying orthogonal translational machinery (data not shown). We will clarify this in the revised manuscript.

Referees cross-commenting

I fully agree with the following remarks from Reviewers #3, #4 and #5. This is a point that I have raised in my report too. The authors need better images to show the periodicity we visualization, and a quantification would be of great benefit to support the claim with numbers (and how these compare to similar studies in the literature):

© 2023. Published by The Company of Biologists under the terms of the Creative Commons Attribution License (https://creativecommons.org/licenses/by/4.0/).

R3: 2. For the dSTORM analysis of the tagged Nav1.6 protein, I also cannot tell there is periodic organization from the image directly. Some analysis is needed there.
R4: 2."As there was no obvious difference in the nanoscale organization of the NaV1.6WT 317 -HA or NaV1.6TAG 318 -HA channels (Fig. 4. e-g), these experiments confirmed that the NaV1.6 overexpression, TCO*A319 Lys incorporation, and click labeling did not affect the nanoscale periodic organization of the sodium channels in the AIS." It is clearly noticeable that for WT, the spot density is more compared to the other two mutants. Why is that so? Using cluster analysis, one can quantify spot density and discuss nanoscale organization quantitatively. The author should quantify the periodicity and compare it among different variants and with previous reports.
R5: 3. The authors claim that there was no obvious difference in the nanoscale organization of the NaV1.6WT 317 -HA or NaV1.6TAG 318 -HA channels (Fig. 4. e-g), but it is hard to conclude this without any quantification and statistical analysis. Sodium channels have been shown to be associated with the membrane-associated periodic skeleton structures in neurons and average autocorrelation analysis has been developed to quantify the degree of periodicity of such structural organizations (Han et al. PNAS 114(32)E6678-E6685, 2017). The authors should use this approach to quantify and compare the average autocorrelation amplitudes.

OUR RESPONSE: As we outlined in our responses to the individual reviewers' comments below, we will address these questions by performing new experiments and quantifications.

I also agree with these comments from Reviewers #3 and #5:

R3: 4. It is unclear, for all the presented data, whether all the cells are collected from a single biological replicate or from multiple replicates. At least 2-3 replicates are needed to see the reproducibility in terms of labeling efficiency, and other related conclusions.
R5: 1. The authors should indicate how many replicates were performed and how many cells were analyzed for each experiment.

OUR RESPONSE: We thank the reviewers for bringing this up. By mistake, we omitted this important information. We will include it in the revised manuscript, but we would like to highlight here that each experiment was repeated at least 3 times.

Reviewer #3

1. There is some patch-like background from the 488 channel from the click reaction, some of which have very as strong signal as the staining on the neurons. What is the potential cause for this? With immunostaining on HA, the background doesn't affect too much on the image data interpretation. However, the major goal of this method development is to use it in live-cell without immunostaining. Without another reference, the high background might cause issues in data interpretation. Can the author also suggest way to avoid or lower this in the discussion?

OUR RESPONSE: We thank the reviewer for bringing this up. We have occasionally observed patch-like background in what appears to be the cell debris. Such dead cells do not have an intact cell membrane and therefore can absorb cell-impermeable ATTO488-tetrazine dye during click labeling. This kind of background is also present in the control neurons transfected with the WT Nav1.6, which suggests that it originates from the UAA and tetrazine-dye accumulations. Additionally, since these patches are not visible with the immunostaining, they do not contain our protein of interest, which further confirms that they contain only dye and UAA accumulations. Depending on the neuron prep/quality before and after transfections, the presence of these patches is more or less obvious. However, despite the background we did not have problems identifying AIS during live cell imaging. Especially when overall neuronal health is optimal after transfections, AIS can easily be distinguished from patches that are positioned outside of labeled neurons. We will investigate this further and discuss it in the revised manuscript.

2. For the dSTORM analysis of the tagged Nav1.6 protein, I also cannot tell there is periodic organization from the image directly. Some analysis is needed there.

OUR RESPONSE: We will address this in the revised manuscript by performing additional experiments and quantifications. We also wrote a detailed answer below, in the response to the

© 2023. Published by The Company of Biologists under the terms of the Creative Commons Attribution License (https://creativecommons.org/licenses/by/4.0/).

other reviewers.

3. The authors use the AIS length as a parameter to evaluate the function of the clickable mutant of NF186, and using patch clamp for functional validation of the clickable mutant of Nav1.6. In both cases, the comparison is done between the mutant and the WT construct, but both in transfected cell and exogenously expressed. It's also worth comparing with untransfected cells as the true native situation.

OUR RESPONSE: We agree with the reviewer that it is important to compare transfected cells with untransfected cells. As the reviewer points out, we have already performed some of these comparisons. When it comes to the NF186, we used the AIS length as a parameter to estimate if the expression of clickable mutant affected the AIS structure. As we show in the Fig. 02, we co-immunostained neurons transfected with NF186-HA WT or TAG constructs. We used HA antibody to detect neurons expressing NF186, while the ankG was used as a marker of the AIS length. To check if the AIS length of transfected cells is affected, we compared the length of transfected cells (expressing NF186, HA+) to surrounding untransfected cells (HA-). When it comes to the Nav1.6, we also compared the AIS length of cells expressing Nav1.6 (HA+) to surrounding untransfected cells (HA-). Similarly to the experiments with NF186, this allowed us to check if the expression of the recombinant Nav1.6 affect the AIS structure. What is missing is the comparison with untransfected conditions (i.e. neurons that are simply stained with ankG). We assume that is what the reviewer is referring to? We will also include these data in the revised manuscript. Furthermore, since we introduced a labeling modification in NaV1.6, we wanted to check if such modification would affect its function. To do so, as routinely done in the field (PMID: 25874799), we rendered the WT and TAG channels TTX-resistant and recorded only recombinant Na+ currents in neuroblastoma cells in the presence of TTX. Perhaps we misunderstand the reviewer's comment, but in this regard measurements of untransfected cells are not relevant since they would not allow us to compare WT and TAG mutants.

4. It is unclear, for all the presented data, whether all the cells are collected from a single biological replicate or from multiple replicates. At least 2-3 replicates are needed to see the reproducibility in terms of labeling efficiency, and other related conclusions.

OUR RESPONSE: We thank the reviewer for the observation. By mistake, we omitted this important information. We will include in the revised version of the manuscript. We would like to highlight here that each experiment was repeated at least 3 times.

Reviewer #4

1."Confocal microscopy revealed that the hNSE promoter lowered the WT and clickable NF186-HA expression levels and consequently improved the localization of these proteins." Is the lower expression level a measure of localization improvement? How does the author conclude that the localization has improved?

OUR RESPONSE: Previous report (PMID: 31900387) suggested that the overexpression of the recombinant WT NF186 can affect its trafficking, leading to the NF186 mislocalization. We observed the same in our experiments with CMV NF186 (in particular for NF186 WT). Hence, based on the PMID: 31900387 we probed weak neuron specific enolase promoter. Since the WT was the most problematic in terms of the ectopic expression, we checked if AIS localization was improved with enolase promoter for this construct. To this aim, we counted number of neurons that with mislocalized signal or with the signal in the AIS for both, CMV and enolase promoter. We could observe that number of neurons with mislocalized signal was lower for enolase promoter. Since there were more neurons with the AIS-specific signal when NF186 was expressed from enolase promoter compared to CMV, we concluded that enolase promoter lowered expression and improved localization of the NF186. Therefore, we used enolase promoter for click labeling of NF186 amber mutants. We will include the results of this analysis in the revised version of the manuscript.

2."As there was no obvious difference in the nanoscale organization of the NaV1.6WT 317 -HA or NaV1.6TAG 318 -HA channels (Fig. 4. e-g), these experiments confirmed that the NaV1.6 overexpression, TCO*A319 Lys incorporation, and click labeling did not affect the nanoscale

© 2023. Published by The Company of Biologists under the terms of the Creative Commons Attribution License (https://creativecommons.org/licenses/by/4.0/).

periodic organization of the sodium channels in the AIS." It is clearly noticeable that for WT, the spot density is more compared to the other two mutants. Why is that so? Using cluster analysis, one can quantify spot density and discuss nanoscale organization quantitatively. The author should quantify the periodicity and compare it among different variants and with previous reports.

OUR RESPONSE: We thank the reviewer for these suggestions. We will address these remarks by performing additional new experiments and quantifications. The difference in the level of the expression of the recombinant Nav1.6 might explain differences in the spot density for WT vs. TAG clickable mutants. However, as the reviewer suggested quantitative analysis is needed to address these concerns. We also intend to quantify the periodicity and compare it among different variants and with previous reports. It is just important to note that in the current version of the manuscript we looked at the nanoscale organization of the subset of Nav1.6 channels. The reason being that we used anti-HA antibody which will only detect our recombinant protein which got incorporated into the AIS and not the endogenous Nav1.6.

Minor comments

1."Although NF186K809TAG 158 -HA (Supplementary Fig. 4) showed bright click labeling, we excluded it from the analysis due to its frequent ectopic expression along the distal axon." How frequently is this bright click labeling observed for this mutation? Is it not observed for other mutations at all? The authors should state this point clearly with some statistics.

OUR RESPONSE: We are not sure what is the exact question from the reviewer. If we understand it correctly, the reviewer is asking us to quantify how frequent was the ectopic expression of this amber mutant compared to other mutants? And not the click labeling (as written in their original comment), since click labeling was observed for all the mutants independently of their ectopic expression?

2."Immunostaining with anti-HA antibody revealed that the expression of NaV1.6WT 239 -HA on the membrane of the N1E-115-1 cells was higher than on the ND7/23 cells (Supplementary Fig. 5a-c). However, click labeling of both NaV1.6K1425TAG 240 -HA and NaV1.6K1546TAG 241 -HA with ATTO488-tz was not successful (Supplementary fig. 5d) indicating insufficient expression of the clickable constructs." Is this due to insufficient expression level or accessibility? The author should make this statement clear.

OUR RESPONSE: We thank the reviewer for bringing this up. We will clarify this in the revised version of the manuscript. We believe that the click labeling of the K1546TAG mutant in N1E-115-1 cells is absent due to the insufficient expression of the channels on the membrane, since this mutant was successfully labeled in the primary neurons that represent more native environment and where Nav1.6 form high-density clusters. K1425TAG mutant is not labeled due to the insufficient expression on the membrane in N1E-115-1 cells as well. However, since this mutant is also poorly labeled in primary neurons, we can speculate that K1425TAG position might be less accessible for the tetrazine-dye compared to K1546TAG. To further support our claim that due to the insufficient expression click labeling is low/absent in neuronal cells, we can use NF186 as an additional example. When NF186 was expressed from strong CMV promoter, we observed click labeling for all the mutants in ND7/23 cells (Suppl. Fig.01). However, when CMV was replaced with neuron specific enolase promoter, the expression was of NF186 was substantially lower in ND7/23 cells and click labeling was absent (data not shown). We will clarify this in the revised manuscript.

3. Authors should clearly state the drift correction procedure of 3D STORM data. What are the localization precision and photon count for 3D STORM experiments?

OUR RESPONSE: We processed 3D dSTORM data in NIS-elements AR software. We used the automatic drift correction from the NIS-elements software that is based on the autocorrelation. We will provide further and updated information in the revised manuscript, including the localization precision and photon count for the new dSTORM images.

4. "Click labeling of NaV1.6 channels in living primary neurons" What kind of primary neurons have been used for click labeling of NaV1.6 channels? Is there any specific reason why authors have

© 2023. Published by The Company of Biologists under the terms of the Creative Commons Attribution License (https://creativecommons.org/licenses/by/4.0/).

chosen cortical neurons for labeling NF186? Does this labeling strategy depend on primary neuron type?

OUR RESPONSE: For the establishment and click labeling of Nav1.6 we used primary rat cortical neurons (Fig. 03, Fig. 06). The same neuronal type has been used for click labeling of NF186 (Fig. 02). We established labeling of the AIS components in cortical neurons because we use those routinely in the laboratory. However, this labeling strategy does not depend on the neuronal type. As we show in Fig. 05, to study localization of the loss-of-function pathogenic Nav1.6 variants we used mouse hippocampal neurons. The reason for this is that in previous study the same neuronal type was used to characterize these two mutations (lines 361-362). This demonstrates nicely that method can be easily transferred to any neuronal type. Furthermore, we were also able to label Nav1.6 and NF186 in mouse cortical neurons (data are not shown in the manuscript). We will clarify this in the revised manuscript.

Reviewer #5

1.Throughout the manuscript, only one representative image containing one AIG is shown for each condition without statistics and quantifications, so the conclusions are not sufficiently convincing. For example, in Fig. 1b, c, e; Fig. 2b,c,d,e; Fig. 3b,c,d,e ; Fig. 5c; and Supplementary Fig.1-6, the authors should quantify the average fluorescence intensities both for HA immunostaining and ATTO488-tz labeling in different conditions, as well as the labeling ratios (fluorescence intensity ratios between ATTO488 and AF647/AF555) . Without statistics and quantifications, it is unclear whether there is any significant difference between the constructs with different TAG positions, or between different transfection methods (e.g., lipofectamine 2000 vs 3000).

OUR RESPONSE: We agree with the reviewer that the quantitative analysis is important and we will provide more quantitative data in the revised manuscript. At the same time, we are a bit confused by this comment which seems to refer to missing quantifications in one of the schemes (Fig. 1) and overlooks existing quantifications (e.g. quantitative analysis of the data set from Fig. 5c is shown in Fig. 5d). However, as suggested by the reviewer and to strengthen our data, in addition to the quantifications already provided in the manuscript (e.g. Fig. 2d: AIS length of NF186TAG constructs; Fig. 3f: AIS length of Nav1.6 TAG constructs; Fig. 5d: click-labeling intensity of LOF mutants), we intend to quantify the differences between labeling ratios of different mutants and transfection methods. When it comes to the different transfection methods, some data is already provided in the manuscript (e.g. we counted number of transfected versus transduced neurons) but we intend to clarify and expand on this in the revised manuscript.

2. The only quantification done was for the average AIS length, but the statistical tests should be performed between different conditions and the corresponding P values should be provided. It seems that the transfected neurons generally have a longer AIS length than the transfected neurons (Fig. 2d and 3f). Could the authors provide an explanation for this?

OUR RESPONSE: We are a bit confused by the first part of this comment. We measured the AIS lengths of NF186 WT or NF186 TAG as well as Nav1.6 TAG and compared it to the AIS lengths of surrounding untransfected cells (Fig. 2d and Fig.03f). In addition, we compared the AIS lengths of the NF186 WT and TAG to each other, and Nav1.6 TAG to each other. To analyze the differences, we performed statistical tests and provided the corresponding p values in the figure legends (Fig. 02 and 03). Further details on the statistical analysis are provided in supplementary tables (Suppl. table 01 and 02). Regarding the 2nd question, we have also noticed that the AIS lengths of transfected neurons appear longer than those of untransfected cells. This seems to be more pronounced in the case of NF186 which is expressed at the higher level compared to the Nav1.6. The appearance of slightly longer AIS is most likely the consequence of the fact that recombinant constructs are overexpressed in the neurons that express endogenous NF186 and Nav1.6. However, this difference in the AIS length is not significant to the controls. We will discuss this further in the revised manuscript.

3. The authors claim that there was no obvious difference in the nanoscale organization of the NaV1.6WT 317 -HA or NaV1.6TAG 318 -HA channels (Fig. 4. e-g), but it is hard to conclude this without any quantification and statistical analysis. Sodium channels have been shown to be associated with the membrane-associated periodic skeleton structures in neurons and average

© 2023. Published by The Company of Biologists under the terms of the Creative Commons Attribution License (https://creativecommons.org/licenses/by/4.0/).

autocorrelation analysis has been developed to quantify the degree of periodicity of such structural organizations (Han et al. PNAS 114(32)E6678-E6685, 2017). The authors should use this approach to quantify and compare the average autocorrelation amplitudes.

OUR RESPONSE: We are thankful to the reviewer for suggestions on how to quantify the periodicity of recombinant sodium channels and how to more accurately compare WT and TAG mutants at the nanoscale level. We will perform additional experiments and analysis in order to address the concerns of this and other reviewers.

4. The authors should also obtain dSTORM images for the click labeled neurons to demonstrate if the click labeling method would provide sufficient labeling efficiency for dSTORM, compared to immunostaining (HA and Ankyrin G immunostaining).

OUR RESPONSE: We would like to thank the reviewer for this suggestion. We have already shown in our previous work that STED can be performed with click labeled neurons (PMID: 35031604). When it comes to this manuscript and AIS labeling, we have already obtained preliminary dSTORM images of click-labeled NF186. Since the expression of Nav1.6 is lower compared to NF186, the labeling is also less bright and dSTORM is a bit more challenging. To try to overcome this issue, in addition to dSTORM of click-labeled Nav1.6, we are planning to try click-PAINT (PMID: 27804198). Click-PAINT has been used for super-resolution imaging of less abundant targets in cells and could possibly allow super-resolution imaging of Nav1.6. We will report on these new experiments in the revised version of the manuscript.

5. It seems that the click labeling has a off-target/background labeling in the soma of the neuron (see Fig. 3c,d. Could the authors quantify and determine the sources of such off-target labeling?

OUR RESPONSE: We thank the reviewer for pointing this out. We will clarify this in the revised manuscript, but by looking at the other examples from our dataset it appears to us that this background is present in WT constructs as well. In the current version of the manuscript, this is not clear since the WT image that is shown in the Fig. 03b is a single plane confocal image. Therefore, we will replace it in the revised manuscript with a z-stack in which the presence of the background is more obvious (due to the maximum intensity projection). In addition, we will conduct additional control experiments to clarify this.

Minor comments:

1. The authors should indicate how many replicates were performed and how many cells were analyzed for each experiment.

OUR RESPONSE: We thank the reviewer for bringing this up. By mistake, we omitted this important information. We will include this information in the revised manuscript, but we would like to highlight here that each experiment was repeated at least 3 times.

2. The display range (i.e., intensity scale bar) was indicated only for a small portion of the fluorescence images. It is better to be consistent and show the display range for all images presented.

OUR RESPONSE: We will include intensity scale bars in all the images in the revised version of the manuscript.

3. Description of the revisions that have already been incorporated in the transferred manuscript

Not applicable.

4. Description of analyses that authors prefer not to carry out
Reviewer #3, comment #5. One application presented in this manuscript is to evaluate the effect of epilepsy-causing mutations of Nav1.6. By comparing the intensity of ATTO488, the result suggests that there is no significant impact of these mutations on membrane tracking. I am wondering if the author should study the membrane tracking by also looking at the diffusion in live-cell with the

© 2023. Published by The Company of Biologists under the terms of the Creative Commons Attribution License (https://creativecommons.org/licenses/by/4.0/).

labeling method. The comparison of the intensity only can be achieved by just immunostaining. It doesn't really demonstrate the benefit of live-cell labeling and imaging with the presented method.

OUR RESPONSE: Generally speaking, one of the advantages of click labeling is its compatibility with live cell labeling. As the reviewer also points out, this is especially useful for live-cell imaging but is not limited to it. In addition, click labeling allows selective labeling of membrane population of Nav1.6 in living neurons. We took advantage of this and used cell-impermeable dyes to label unnatural amino acids incorporated into extracellular part of Nav1.6 (Scheme 03a). On the contrary, HA tag that allows immunodetection of recombinant Nav1.6 is added to the intracellular C terminus. Hence, by anti-HA immunostaining total (intra- and extracellular) epilepsy-causing Nav1.6 channel population will be detected. That is why in this case live-cell click labeling was advantageous compared to the conventional immunostaining. We will clarify this in the revised manuscript. In addition, we would like to note that when we started the experiments with the epilepsy-causing mutations, we wanted to a) check if they are present on the membrane and b) depending on the outcome of those experiments follow the trafficking of these LOF Nav1.6 mutants. Since patch clamp recordings of pathogenic Nav1.6 showed loss of Na+ currents, we at first assumed that they are not properly expressed on the membrane. However, our click labeling showed that the pathogenic channels were detected at the AIS membrane despite the loss of Na+ currents. This was also somewhat surprising to us and we would love to investigate this further. We also appreciate the reviewer's suggestion in this regard and we hope to be able to use all the advantages of our labeling approach in our follow-up studies. However, keeping in mind time and resources limitations, live-cell trafficking study might be beyond the scope of this revision.

Original submission

<u>First decision letter</u>

MS ID#: JOCES/2022/260600

MS TITLE: Direct fluorescent labeling of NF186 and NaV1.6 in living primary neurons using bioorthogonal click chemistry

AUTHORS: Nevena Stajkovic, Yuanyuan Liu, Aleksandra Arsic, Ning Meng, Hang Lyu, Nan Zhang, Dirk Grimm, Holger Lerche, and Ivana Nikic-Spiegel
ARTICLE TYPE: Research Article

We have now reached a decision on the above manuscript based on the Review Commons reports and your extensive list of proposed changes. II would be pleased to see a revised manuscript which contains the proposed amendments, together with a point-by-point rebuttal letter in which you confirm each and every proposed changes as the file included in the present submission. If you do not agree with any of their criticisms or suggestions please explain clearly why this is so. Please also highlight major changes in the revision plan.

Please ensure that you clearly highlight all changes made in the revised manuscript. Please avoid using 'Tracked changes' in Word files as these are lost in PDF conversion.

First revision

<u>Author response to reviewers' comments</u>

Manuscript number: JOCES/2022/260600
(review commons manuscript number RC-2022-01341)

© 2023. Published by The Company of Biologists under the terms of the Creative Commons Attribution License (https://creativecommons.org/licenses/by/4.0/).

1. General Statements

We would like to thank the reviewers for their insightful comments and their overall positive evaluation of the significance of our results. Based on the reviewers' comments and our initial revision plan, we performed new experiments and new quantitative analyses. These are outlined in detail below in our point-by-point response. As the revision produced a significant amount of new data, we had to rewrite and shorten some parts of the manuscript and rearrange the figures. We also modified our manuscript to meet the formatting requirements. Except for smaller edits for grammar or style, changes to the manuscript are highlighted in yellow in the main text and supplementary information files.

2. Description of the performed revisions

Reviewer #2

- On lines 107 and 108, the sentence "The C-terminal HA-tag allowed us to detect the full-length NF-186 protein by immunostaining it with an anti-HA antibody" would have a better place just after lines 104-105 " [...] we modified the previously described plasmid (Zhang et al., 1998) by moving the hemagglutinin (HA) tag from the N terminus to the C terminus".

Our response: As the reviewer suggested, we changed the position of that sentence. However, as part of this revision, we had to shorten the main text and the respective description was moved to the Methods section.

- Fig.2b: the AnkG staining looks substantially longer than that showed in c. However, the results on AIS length show no significant changes in between the groups. This is visually misleading, the authors should choose a picture for the WT construct that is representative of the data.

Our response: We thank the reviewer for bringing this up. After doing additional quantitative analyses involving the fluorescence intensity measurements of the NF186 in the AIS (Fig. 2F), we found out that the previously shown NF186TAG example was not the most representative in terms of its intensity in the ATTO488 channel. Thus, we decided to keep the WT example and replace the amber mutant example. These examples are also more similar in terms of their lengths.

- Line 238: what is the rationale behind choosing these cells? For example, have they been used in other studies for similar purposes? If so, please provide the reference.

Our response: In our initial experiments, we probed neuroblastoma ND7/23 cells which are commonly used for the electrophysiological recordings of recombinant Nav1.6 (PMID: 30615093, 22623668, 25874799, 7375106). Although we were able to record Na^+ currents in those cells, only a small portion of channels was detected on the cell surface by microscopy (Fig. S5A). As we discuss in the manuscript, we then switched to N1E-115-1 cells in which we obtained a higher level of expression of the recombinant NaV1.6 channels on the cell surface (Fig. S5B). These cells have also been previously used for electrophysiological studies of voltage-gated sodium channels, including Nav1.6 (PMID: 8822380, 24077057). We have included this explanation and references in the revised manuscript.

- Figure 3c, the authors omitted the comparison with the WT construct this time, as opposed to the neurofascin experiments. What is the reason?

Our response: As previously reported (PMID: 31900387) and also observed in our study, overexpression of recombinant NF186 in neurons can lead to its mislocalization. In our hands, this was evident for highly expressed WT and certain amber mutants (i.e., K809TAG). To address this issue, based on the previous report (PMID: 31900387), we tested a weaker human neuron- specific enolase promoter. The enolase promoter reduced expression levels and improved localization of NF186. However, we still observed some neurons with mislocalized NF186 WT. To quantify this effect, we compared the AIS length of the WT construct and amber mutants to surrounding untransfected cells. Since we did not have overexpression and mislocalization problems with the

© 2023. Published by The Company of Biologists under the terms of the Creative Commons Attribution License (https://creativecommons.org/licenses/by/4.0/).

Nav1.6 WT construct (all observed neurons had Nav1.6 localizing in the AIS), we measured only the AIS length of the amber mutants. However, to strengthen our findings, as part of the revision, we performed new experiments including the WT construct, acquired new images, and performed a new quantitative analysis. This analysis did not reveal any significant differences between the WT and TAG expressing neurons. The results are shown in Fig. 4A-B.

- Fig. 4: why did the authors chose these cells for electrophysiology experiments and not neurons? Explain the rationale in the text or, alternatively, cite similar studies using the same tool.

Our response: Due to the branched neuronal processes which cause the space clamp problem in voltage clamp experiments with neurons, round and none-branching cells are frequently used to examine the biophysical properties of ion channels, including Nav1.6. Most of the previous studies investigating the biophysical properties of NaV1.6 channels were performed in neuroblastoma cells, such as ND7/23 and N1E-115-1 cells (PMID: 25874799; 25242737). We tested these two types of cells and found that N1E-115-1 cells supported a higher expression level of the recombinant NaV1.6 channels on the cell surface than the ND7/23 cells (Fig S5). Hence, N1E-115-1 cells were more suitable to get robust and reliable recordings (as we also discussed above in response to the reviewer's comment).

- Fig.4, biophysical properties: did the authors find differences in passive properties? Measures of resting potential, membrane resistance and cell capacitance should be reported.

Our response: Passive properties such as resting membrane potential and membrane resistance are important functional features in neurons measured in current clamp experiments but are not applicable for ND7/23 and N1E-115-1 cells used in our voltage clamp experiments. To measure the Na^+ current mediated by WT or mutant NaV1.6 channels expressed in N1E-115-1 cells, the endogenous Na^+ channels were blocked by tetrodotoxin and the endogenous K^+ channels were blocked by tetraethylammonium chloride, CsCl and CsF in extracellular and intracellular solutions. Under these conditions, resting potential and membrane resistance are not relevant for experiments. Cell capacitance reflects the size of the cell surface area, which can affect the number of channels expressed on the cell surface. To eliminate the effect of different cell sizes, Na^+ current densities normalized by cell capacitances were used in our experiments. These values are reported in the manuscript (Table S15).

- Fig 4, STORM images. The periodic distribution of the dots should be enhanced with some sort of arrows or lines, for the non-specialist audience.

Our response: Based on the comments from multiple reviewers, we carried out quantitative analyses of the nanoscale organization of immunostained neurons expressing recombinant WT and TAG Nav1.6 (Fig. 7). To improve the suitability of our dataset for quantitative analyses, we performed new experiments in which we also included panNav-immunostained controls. We analyzed periodicity and spacing and report these values (Fig. 7).

- Line 374: rat or mouse primary neurons?

Our response: In the manuscript, we were referring to both rat and mouse neurons. The images are shown in Fig. 5 and Suppl. Fig. 8 were obtained from rat cortical neurons expressing Nav1.6 or fluorescent reporter. However, we were also able to successfully transduce mouse neurons with AAV92A carrying orthogonal translational machinery (data not shown). We clarify this in the revised manuscript.

Referees cross-commenting

I fully agree with the following remarks from Reviewers #3, #4 and #5. This is a point that I have raised in my report too. The authors need better images to show the periodicity we visualization, and a quantification would be of great benefit to support the claim with numbers (and how these compare to similar studies in the literature):

© 2023. Published by The Company of Biologists under the terms of the Creative Commons Attribution License (https://creativecommons.org/licenses/by/4.0/).

R3: 2. For the dSTORM analysis of the tagged Nav1.6 protein, I also cannot tell there is periodic organization from the image directly. Some analysis is needed there. R4: 2."As there was no obvious difference in the nanoscale organization of the NaV1.6WT 317 - HA or NaV1.6TAG 318 -HA channels (Fig. 4. e-g), these experiments confirmed that the NaV1.6 overexpression, TCO*A319 Lys incorporation, and click labeling did not affect the nanoscale periodic organization of the sodium channels in the AIS." It is clearly noticeable that for WT, the spot density is more compared to the other two mutants. Why is that so? Using cluster analysis, one can quantify spot density and discuss nanoscale organization quantitatively. The author should quantify the periodicity and compare it among different variants and with previous reports. R5: 3. The authors claim that there was no obvious difference in the nanoscale organization of the NaV1.6WT 317 -HA or NaV1.6TAG 318 -HA channels (Fig. 4. e-g), but it is hard to conclude this without any quantification and statistical analysis. Sodium channels have been shown to be associated with the membrane-associated periodic skeleton structures in neurons and average autocorrelation analysis has been developed to quantify the degree of periodicity of such structural organizations (Han et al. PNAS 114(32)E6678-E6685, 2017). The authors should use this approach to quantify and compare the average autocorrelation amplitudes.

Our response: As we also outline in our response to the individual reviewers' comments below, we addressed these questions by performing new experiments and quantitative analyses.

I also agree with these comments from Reviewers #3 and #5:
R3: 4. It is unclear, for all the presented data, whether all the cells are collected from a single biological replicate or from multiple replicates. At least 2-3 replicates are needed to see the reproducibility in terms of labeling efficiency, and other related conclusions. R5: 1. The authors should indicate how many replicates were performed and how many cells were analyzed for each experiment.

Our response: We thank the reviewers for bringing this up. This information is included in the revised manuscript, in the figure legends and the Materials and Method section.

Reviewer #3

1. There is some patch-like background from the 488 channel from the click reaction, some of which have very as strong signal as the staining on the neurons. What is the potential cause for this? With immunostaining on HA, the background doesn't affect too much on the image data interpretation. However, the major goal of this method development is to use it in live-cell without immunostaining. Without another reference, the high background might cause issues in data interpretation. Can the author also suggest way to avoid or lower this in the discussion?

Our response: We thank the reviewer for bringing this to our attention. We have observed patch-like signal occasionally, which we believe to be cell debris. We have included this in the revised manuscript. These dead cells lack an intact cell membrane and, therefore can absorb ATTO488-tetrazine dye during click labeling. This kind of background is also observed in the control neurons transfected with the WT Nav1.6, mock-transfected neurons and even untransfected neurons, which suggests that it originates from the dye accumulations. Furthermore, the absence of these patches in the immunostaining confirms that they contain only dye and not our protein of interest. Depending on the neuron prep/quality before and after transfections, the visibility of these patches is more or less obvious. Despite this, we did not have problems identifying AIS during live cell imaging. Especially when overall neuronal health is optimal after transfections, AIS can easily be distinguished from patches that are positioned outside of labeled neurons.

2. For the dSTORM analysis of the tagged Nav1.6 protein, I also cannot tell there is periodic organization from the image directly. Some analysis is needed there.

Our response: In response to this request and that of the other reviewers, we carried out quantitative analyses of the nanoscale organization of immunostained neurons expressing recombinant WT and TAG Nav1.6 (Fig. 7). To improve the suitability of our dataset for quantitative analyses, we performed new experiments in which we also included panNav-immunostained controls. We analyzed periodicity and spacing and report these values (Fig. 7).

© 2023. Published by The Company of Biologists under the terms of the Creative Commons Attribution License (https://creativecommons.org/licenses/by/4.0/).

3. The authors use the AIS length as a parameter to evaluate the function of the clickable mutant of NF186, and using patch clamp for functional validation of the clickable mutant of Nav1.6. In both cases, the comparison is done between the mutant and the WT construct, but both in transfected cell and exogenously expressed. It's also worth comparing with untransfected cells as the true native situation.

Our response: We agree with the reviewer that it is important to compare transfected cells with untransfected cells. As the reviewer pointed out, we have already performed some of these comparisons in the original version of the manuscript.

With regards to NF186, we used the AIS length as a parameter to estimate if the expression of clickable mutant affected the AIS structure. As we show in Fig. 2E, we co-immunostained neurons transfected with NF186-HA WT or TAG constructs. We used an HA antibody to detect neurons expressing NF186 and ankG as a marker of the AIS length. To check if the AIS length of transfected cells is affected, we compared the AIS length (Fig. 2E, Fig. 4A) of transfected cells (expressing recombinant NF186, HA+) to surrounding (neighbouring) untransfected cells (HA−). In addition, we now also compared the length of mock-transfected neurons (cells were transfected with an “empty” plasmid to exclude the effect of the transfection on the AIS length) to all the above-mentioned conditions (neurons expressing NF186 and neighbouring untransfected neurons). There was no significant difference observed between these groups (Fig.2E and figure below).

NOTE: We have removed unpublished data that had been provided for the referees in confidence.

With regards to Nav1.6, we also compared the AIS length of neurons expressing Nav1.6 (HA+) to surrounding untransfected cells (HA−). Similarly to the experiments with NF186, this allowed us to check if the expression of the recombinant Nav1.6 affected the AIS length. As part of this revision, we performed new experiments in order to include WT Nav1.6 constructs and mock- transfected controls. No significant difference were observed between the groups (Fig. 4A and figure below).

NOTE: We have removed unpublished data that had been provided for the referees in confidence.

As we thought that neighbouring untransfected neurons were more relevant for comparison and we saw no difference compared to mock-transfected neurons, we did not include the latter in the manuscript. However, we attach the results here, and if the reviewer thinks they are required, we can also include the values for mock-transfected neurons in the final version of the manuscript.
With regards to electrophysiology, since we introduced a labeling modification in NaV1.6, we wanted to check if such a modification would affect its function. To do so, as routinely done in the field (PMID: 25874799), we rendered the WT and TAG channels TTX-resistant and recorded only recombinant Na+ currents in neuroblastoma cells in the presence of TTX. Perhaps we misunderstand the reviewer’s comment, but in this regard, measurements of untransfected cells are not relevant since they would not allow us to compare WT and TAG mutants.

4. It is unclear, for all the presented data, whether all the cells are collected from a single biological replicate or from multiple replicates. At least 2-3 replicates are needed to see the reproducibility in terms of labeling efficiency, and other related conclusions.

Our response: We thank the reviewers for bringing this up. This information is included in the revised manuscript, in the figure legends and the Materials and Method section.

Reviewer #4

1."Confocal microscopy revealed that the hNSE promoter lowered the WT and clickable NF186- HA expression levels and consequently improved the localization of these proteins." Is the lower expression level a measure of localization improvement? How does the author conclude that the localization has improved?

Our response: A previous report (PMID: 31900387) suggested that the overexpression of the recombinant WT NF186 can lead to its mislocalization. We observed similar results in our experiments with CMV-NF186 (in particular for NF186 WT). Hence, based on the PMID:

© 2023. Published by The Company of Biologists under the terms of the Creative Commons Attribution License (https://creativecommons.org/licenses/by/4.0/).

31900387, we probed a weaker neuron-specific enolase promoter. Since the WT was the worst in terms of ectopic expression, we tested if using the enolase promoter for the expression of WT NF186 could improve its AIS localization. To this aim, for both CMV and enolase promoters, we counted the number of neurons expressing WT NF186 with mislocalized signal (allong dendrites and axons) or with the signal in the AIS. Our analysis indicated that the number of neurons with mislocalized signals was lower when the enolase promoter was used. Since there were more neurons with the AIS-specific signal when NF186 was expressed from the enolase promoter compared to CMV, we concluded that the enolase promoter lowered expression and improved localization of the NF186. Therefore, we used the enolase promoter for click labeling of NF186 amber mutants. We included the results of this analysis in the revised manuscript.

2."As there was no obvious difference in the nanoscale organization of the NaV1.6WT 317 -HA or NaV1.6TAG 318 -HA channels (Fig. 4. e-g), these experiments confirmed that the NaV1.6 overexpression, TCO*A319 Lys incorporation, and click labeling did not affect the nanoscale periodic organization of the sodium channels in the AIS." It is clearly noticeable that for WT, the spot density is more compared to the other two mutants. Why is that so? Using cluster analysis, one can quantify spot density and discuss nanoscale organization quantitatively. The author should quantify the periodicity and compare it among different variants and with previous reports.

Our response: In response to this comment and those of the other reviewers, we carried out quantitative analyses of the nanoscale organization of immunostained neurons expressing recombinant WT and TAG Nav1.6 (Fig. 7). To improve the suitability of our dataset for quantitative analyses, we performed new experiments in which we also included panNav-immunostained controls. We analyzed periodicity and spacing and report these values (Fig. 7). We discuss this in detail in the manuscript, but summarized brielfy we did not notice significant dfferences between the groups.

Minor comments

1."Although NF186K809TAG 158 -HA (Supplementary Fig. 4) showed bright click labeling, we excluded it from the analysis due to its frequent ectopic expression along the distal axon." How frequently is this bright click labeling observed for this mutation? Is it not observed for other mutations at all? The authors should state this point clearly with some statistics.

Our response: We appreciate the comment of the reviewer, but we were not entirely sure what was the exact question. If we understand it correctly, the reviewer asked us to quantify the frequency of the K809TAG NF186 ectopic expression compared to other mutants? Or how frequent was ectopic click labeling (as seems to be written in their original comment)? We would like to clarify that the click labeling was observed for all the mutants independently of their expression pattern. The click labeling itself was not ectopic, but it was co-localizing with the HA signal. The HA signal appeared ectopic (NF186-HA present along distal axons), and for K809TAG was observed in the majority of neurons. We tried to clarify this in the revised text. Because of its expression pattern, we did not acquire enough images of K809TAG during initial experiments. To quantitatively address this, we would have needed to perform a new set of experiments with all of the mutants. While we performed such experiments for other analyses during this revision, we thought it was not relevant since we determined that three other mutants (i.e. K519TAG, K604TAG, K680TAG) show proper localization and labeling. As these are the main criteria for amber mutant selection for click labeling, the quantification of K809TAG ectopic expression would not have added relevant new information.

2."Immunostaining with anti-HA antibody revealed that the expression of NaV1.6WT 239 -HA on the membrane of the N1E-115-1 cells was higher than on the ND7/23 cells (Supplementary Fig. 5a-c). However, click labeling of both NaV1.6K1425TAG 240 -HA and NaV1.6K1546TAG 241 - HA with ATTO488-tz was not successful (Supplementary fig. 5d) indicating insufficient expression of the clickable constructs." Is this due to insufficient expression level or accessibility? The author should make this statement clear.

Our response: We thank the reviewer for bringing this to our attention. Keeping in mind our results with primary neurons, we believe that the absence of noticable click labeling of the K1546TAG

© 2023. Published by The Company of Biologists under the terms of the Creative Commons Attribution License (https://creativecommons.org/licenses/by/4.0/).

mutant in N1E-115-1 cells is due to the insufficient expression of the channels on the membrane. Namely, in subsequent experiments this mutant was successfully labeled in the primary neurons that provide more native environment for expression of sodium channels and where Nav1.6 form high-density clusters at the AIS. Similarly, we could also speculate that the K1425TAG mutant is not labeled due to the insufficient expression on the membrane in N1E-115-1 cells. However, the quantifications performed during this revision suggest that the K1425TAG position is less accessible for the tetrazine-dye compared to K1546TAG. This is also supported by AlphaFold predicted 3D structures, as we also discuss in the revised manuscript. We clarify this in the revised manuscript by changing the original sentence to "However, click labeling with ATTO488-tz was not successful on either NaV1.6^{K1425TAG}-HA nor NaV1.6^{K1546}TAG-HA (Fig. S5D), most likely due to insufficient expression of these constructs". We discuss the dye accessibility later in the text in the context of results obtained with primary neurons (Fig. 3D).

To further support our claim that click labeling can be low/absent in neuroblastoma cells due to insufficient expression, we can use NF186 as an additional example. When NF186 was expressed from a strong CMV promoter, we observed click labeling for all the mutants in ND7/23 cells (Fig. S1). However, when the CMV was replaced with neuron-specific enolase promoter, the expression of NF186 was substantially lower and click labeling was absent (data not shown).

3. Authors should clearly state the drift correction procedure of 3D STORM data. What are the localization precision and photon count for 3D STORM experiments?

Our response: As we processed the images using the NIS Elements STORM module, we used the automatic drift correction (based on autocorrelation) from the NIS elements software. We describe this, including the information on localization precision and photon counts in the revised methods section and supplementary information (Table S26).

4. "Click labeling of NaV1.6 channels in living primary neurons" What kind of primary neurons have been used for click labeling of NaV1.6 channels? Is there any specific reason why authors have chosen cortical neurons for labeling NF186? Does this labeling strategy depend on primary neuron type?

Our response: For the establishment and click labeling of Nav1.6, we used primary rat cortical neurons (Fig. 3, Fig. 4, Fig. 6, Fig.7, Fig. 8). The same neuronal type has been used for click labeling of NF186 (Fig. 2). We established labeling of the AIS components in cortical neurons because we use those routinely in the laboratory. However, this labeling strategy is not limited to this specific neuronal type. As we show in Fig. 5, to study the localization of the loss-of-function pathogenic Nav1.6 variants, we used mouse hippocampal neurons. The reason for this is that in a previous study, the same neuronal type was used to characterize these two mutations. This demonstrates that this method can be easily transferred to any neuronal type. In other work, we were also able to label Nav1.6 and NF186 in mouse cortical neurons.

Reviewer #5

1. Throughout the manuscript, only one representative image containing one AIG is shown for each condition without statistics and quantifications, so the conclusions are not sufficiently convincing. For example, in Fig. 1b, c, e; Fig. 2b,c,d,e; Fig. 3b,c,d,e ; Fig. 5c; and Supplementary Fig.1-6, the authors should quantify the average fluorescence intensities both for HA immunostaining and ATTO488-tz labeling in different conditions, as well as the labeling ratios (fluorescence intensity ratios between ATTO488 and AF647/AF555) . Without statistics and quantifications, it is unclear whether there is any significant difference between the constructs with different TAG positions, or between different transfection methods (e.g., lipofectamine 2000 vs 3000).

Our response: To strengthen our findings, we have provided more quantitative data in the revised manuscript. In addition to the quantifications from the previous version of the manuscript (i.e., old Fig. 2d: AIS length of neurons expressing NF186TAG constructs; old Fig. 3f: AIS length of neurons expressing Nav1.6 TAG constructs; old Fig. 5d: click labeling intensity of LOF mutants), we performed new quantitative analyses on existing and new datasets. As the reviewer suggested, we

© 2023. Published by The Company of Biologists under the terms of the Creative Commons Attribution License (https://creativecommons.org/licenses/by/4.0/).

quantified the average fluorescence intensities for HA immunostaining and ATTO488-tz labeling, as well as the labeling ratios for different constructs (Fig. 2F for NF186; Fig. 3D for Nav1.6); we quantified cytosolic ATTO488-tz signal (Fig. S6C); we quantitatively compared different transfection methods (Fig.3E-F), including AAVs (Fig. 6C that was obtained with a new dataset); and we performed quantitative dSTORM analyses (Fig. 2J-L, Fig. 7, Fig.8). Moreover, we performed new analysis of the Nav1.6 AIS length to include WT Nav1.6 (old Fig.3f, new Fig. 4A that was obtained with a new dataset) and we updated the LOF analysis (old Fig. 5d, new Fig. 5D) to include the analysis of an additional experiment (that was completed after the submission of the original version).

2. The only quantification done was for the average AIS length, but the statistical tests should be performed between different conditions and the corresponding P values should be provided. It seems that the transfected neurons generally have a longer AIS length than the transfected neurons (Fig. 2d and 3f). Could the authors provide an explanation for this?

Our response: We are a bit confused by the first part of this comment. In the first version of the manuscript, we measured the AIS lengths of NF186 WT or NF186 TAG as well as Nav1.6 TAG and compared it to the AIS lengths of surrounding untransfected cells (old Fig. 2d and Fig.03f). In addition, we compared the AIS lengths of NF186 WT and TAG to each other, and Nav1.6 TAG to each other. To analyze the differences, we performed statistical tests for multiple comparisons and provided the corresponding *p* values in the figure legends (old Fig. 02 and 03). Further details on the statistical analysis were provided in supplementary tables (old Suppl. table 01 and 02). These data are also included in the revised manuscript (Fig. 2E, Fig. 4A, Table S1, Table S15).

Regarding the second question, we have also noticed that the AIS lengths of transfected neurons appear somewhat longer than those of untransfected cells. This applied to both WT and TAG constructs and, because of that, is not the consequence of our labeling approach. Independence of our labeling approach was the most important criterion for us when establishing this method. Otherwise, we can only speculate about this. The observed difference in the length is most likely the consequence of the fact that recombinant constructs are overexpressed in the neurons that contain endogenous NF186 and Nav1.6. In line with this, this was more pronounced in the case of NF186, which is expressed at a higher level compared to Nav1.6. However, measured AIS lengths were not significantly different between different conditions in our experiments involving both NF186 and Nav1.6. Furthermore, in our new dataset that we obtained for the quantification of the Nav1.6 AIS length including WT Nav1.6 (new Fig. 4A), we measured more similar AIS lengths. For these experiments we used a different ankG antibody that provided stronger immunostaining signal, which might have contributed to more precise measurements.

3. The authors claim that there was no obvious difference in the nanoscale organization of the NaV1.6WT 317 -HA or NaV1.6TAG 318 -HA channels (Fig. 4. e-g), but it is hard to conclude this without any quantification and statistical analysis. Sodium channels have been shown to be associated with the membrane-associated periodic skeleton structures in neurons and average autocorrelation analysis has been developed to quantify the degree of periodicity of such structural organizations (Han et al. PNAS 114(32)E6678-E6685, 2017). The authors should use this approach to quantify and compare the average autocorrelation amplitudes.

Our response: We thank the reviewer for suggestions on how to quantify the periodicity of recombinant Nav and how to more accurately compare WT and TAG mutants at the nanoscale level. Based on this and comments from other reviewers, we performed new experiments and quantitatively analyzed the nanoscale organization of immunostained neurons expressing recombinant Nav1.6 (Fig. 7). In these experiments, we compared HA-immunostaining of recombinant Nav1.6 to panNav immunostaining, which was previously used to study the nanoscale organization of sodium channels in the AIS. Importantly, our analysis did not reveal any significant difference between the panNav and our recombinant constructs in terms of their spacing and degree of periodicity (autocorrelation amplitudes). The values for K1425TAG were lower than those of K1546TAG, but this is not surprising consider its lower labeling intensity. It is also important to note that in line with what was published for Nav channels, and as we also discuss in the revised manuscript, the periodicity was not uniform. There are many explanations to this, some of which we discuss in the revised manuscript. In this regard, the most important for us was to include panNav controls in our experiments since the quality of dSTORM images and measuered quantitative

© 2023. Published by The Company of Biologists under the terms of the Creative Commons Attribution License (https://creativecommons.org/licenses/by/4.0/).

parameters strongly depend on the imaging and cell preparation settings, such as fixation and immunostaining.

4. The authors should also obtain dSTORM images for the click labeled neurons to demonstrate if the click labeling method would provide sufficient labeling efficiency for dSTORM, compared to immunostaining (HA and Ankyrin G immunostaining).

Our response: We thank the reviewer for this suggestion. We performed new experiments with dSTORM-compatible AlexaFluor647-tetrazine, which allowed us to acquire dSTORM images of click-labeled NF186 and Nav1.6. We quantified the periodicity of click-labeled channels and compared it to HA immunostaining and immunostaining with panNav antibody that had been used previously for dSTORM imaging of Nav in primary neurons. The representative dSTORM images, along with the quantitative analysis of the nanoscale organization of click-labeled AIS components, are presented in Fig. 2H-J and Fig. 8 and discussed in the text.

5. It seems that the click labeling has a off-target/background labeling in the soma of the neuron (see Fig. 3c,d. Could the authors quantify and determine the sources of such off-target labeling?

Our response: We thank the reviewer for pointing this out. By checking other images from our dataset, we observed that this background labeling is not only present in amber mutants but also in WT. We have now modified Fig. 3 to include such a WT example. Furthermore, we conducted new experiments to investigate the origin of this background signal and we carried out quantitative analyses (Fig. S6C-D). Our first asumption was that this could be off-target labeling due to nonspecific amber codon suppression. To address this, neurons were transfected with different combinations of plasmids in the absence and presence of TCO*A-Lys (Fig. S6C). Quantitative analysis of the mean fluorescence intensity did not allow us to identify a single source of the cytosolic signal. We could exclude nonspecific amber codon suppression (off-target labeling) as the source because the cytosols of neurons transfected only in the presence of the genetic code expansion machinery were not stained. Furthermore, we noticed that the signal was the highest in neurons that expressed the full-length NaV1.6 whether it was WT or one of our clickable variants. Although cytosolic ATTO488 fluorescence seemed to vary between different experiments, some of the transfected neurons are permeable to fluorescent dye, even in the absence of the genetic code expansion machinery and UAA. Thus, the cytosolic signal seems to be a combination of nonspecific dye labeling and click-labeled ion channels. Although an important consideration, this has no bearing on our findings: 1) it does not depend on amber codon suppression, which we consider the most relevant in the context of our method; 2) in the few neurons that were affected, the intensity of cytosolic ATTO488 was lower than the specific AIS signal. We discuss this in the revised manuscript.

Minor comments:

1. The authors should indicate how many replicates were performed and how many cells were analyzed for each experiment.

Our response: We thank the reviewer for bringing this up. This information is included in the revised manuscript, in the figure legends and the Materials and Method section.

2. The display range (i.e., intensity scale bar) was indicated only for a small portion of the fluorescence images. It is better to be consistent and show the display range for all images presented.

Our response: We included LUT intensity scale bars in all images in the revised version of the manuscript.

3. Description of analyses that authors prefer not to carry out

Reviewer #3, comment #5. One application presented in this manuscript is to evaluate the effect of epilepsy-causing mutations of Nav1.6. By comparing the intensity of ATTO488, the result suggests that there is no significant impact of these mutations on membrane tracking. I am wondering if the

© 2023. Published by The Company of Biologists under the terms of the Creative Commons Attribution License (https://creativecommons.org/licenses/by/4.0/).

author should study the membrane tracking by also looking at the diffusion in live-cell with the labeling method. The comparison of the intensity only can be achieved by just immunostaining. It doesn't really demonstrate the benefit of live-cell labeling and imaging with the presented method.

Our response: In general, the advantage of click labeling is its compatibility with live cell labeling. As the reviewer also points out, this feature makes click labeling especially useful for live-cell imaging, among the other applications. The unique advantage of click labeling is that it can be used for selective labeling of the membrane population of Nav1.6 in the AIS of living neurons. We took advantage of this and used cell-impermeable dyes to label unnatural amino acids incorporated into the extracellular part of Nav1.6 (Figure 03A). In contrast, the HA tag that allows immunodetection of recombinant Nav1.6 is added to the intracellular C terminus. Hence, by anti-HA immunostaining, total (intra- and extracellular) epilepsy-causing Nav1.6 channel population would be detected. That is why in this case live-cell click labeling was advantageous compared to conventional immunostaining. We clarify this in the revised manuscript. In addition, when we began the experiments with the epilepsy-causing mutations, our goal was to a) determine if the LOF Nav1.6 channels were at all present on the membrane and b), depending on the outcome of those experiments, follow the trafficking of these LOF Nav1.6 mutants. Since patch clamp recordings of pathogenic Nav1.6 showed a loss of Na+ currents, we initially assumed that they were not properly expressed on the membrane. However, our click labeling showed that the pathogenic channels were detected at the AIS membrane despite the loss of Na+ currents. This finding was somewhat surprising and we would love to investigate this further. We also appreciate the reviewer’s suggestion in this regard and plan to use our labeling approach in our follow-up studies also involving other pathogenic mutations. However, as our current manuscript is focused on various methodological aspects related to establishment of this new tool, and is now strengthened with considerable amount of new data, we would not have the capacity to properly perform and discuss results of such live-cell trafficking studies.

Second decision letter

MS ID#: JOCES/2022/260600

MS TITLE: Direct fluorescent labeling of NF186 and NaV1.6 in living primary neurons using bioorthogonal click chemistry

AUTHORS: Nevena Stajkovic, Yuanyuan Liu, Aleksandra Arsic, Ning Meng, Hang Lyu, Nan Zhang, Dirk Grimm, Holger Lerche, and Ivana Nikic-Spiegel
ARTICLE TYPE: Research Article

Thank you for sending your manuscript to Journal of Cell Science through Review Commons.

I am happy to tell you that your manuscript has been accepted for publication in Journal of Cell Science, pending standard ethics checks.

© 2023. Published by The Company of Biologists under the terms of the Creative Commons Attribution License (https://creativecommons.org/licenses/by/4.0/).
